# Design, synthesis, and time-gated cell imaging of carbon-bridged triangulenium dyes with long fluorescence lifetime and red emission†
†Electronic supplementary information (ESI) available: Experimental details and synthetic procedures. Copies of NMR spectra. Crystal structures for compounds **6**, **7**, **8**, and DAOTA and the associated crystal structure data in CIF format. Spectroscopic details, fluorescence excitation and emission anisotropy spectra for **6**, **7**, and **8**. Details and additional data associated with the time-gated measurements. Computational details, calculated Cartesian coordinates and energies from DFT optimized structures, and TDDFT calculated excitation energies and Kohn-Sham orbital plots. CCDC 1557677–1557680. For ESI and crystallographic data in CIF or other electronic format see DOI: 10.1039/c8sc00089a


**DOI:** 10.1039/c8sc00089a

**Published:** 2018-02-23

**Authors:** M. Rosenberg, K. R. Rostgaard, Z. Liao, A. Ø. Madsen, K. L. Martinez, T. Vosch, B. W. Laursen

**Affiliations:** a Nano-Science Center & Department of Chemistry , University of Copenhagen , Universitetsparken 5, DK-2100 , Copenhagen Ø , Denmark . Email: mrtnrosenberg@gmail.com ; Email: bwl@nano.ku.dk; b Department of Pharmacy , University of Copenhagen , Universitetsparken 2, DK-2100 , Copenhagen Ø , Denmark

## Abstract

Time-resolved fluorescence offers many advantages over normal steady-state detection and becomes increasingly important in bioimaging. However, only very few fluorophores with emission in the visible range and fluorescence lifetimes above 5 ns are available. In this work, we prepare a series of new aza/oxa-triangulenium dyes where one of the usual oxa or aza bridges is replaced by an isopropyl bridge. This leads to a significant redshift of fluorescence with only moderate reductions of quantum yields and a unique long fluorescence lifetime. The fluorescence of the isopropyl bridged diazatriangulenium derivative CDATA^+^ is red-shifted by 50 nm (1400 cm^–1^) as compared to the oxygen-bridged DAOTA^+^ chromophore and has intense emission in the red region (600–700 nm) with a quantum yield of 61%, and a fluorescence lifetime of 15.8 ns in apolar solution. When the CDATA^+^ dye is used as cell stain, high photostability and efficient time-gated cell imaging is demonstrated.

## Abstract 2

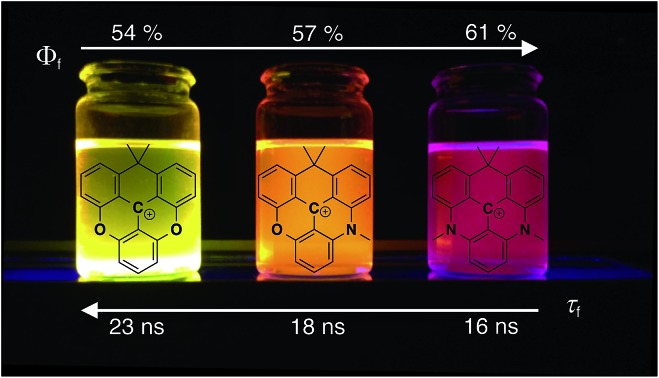
Introduction of an isopropyl bridge in the triangulenium skeleton leads to a new series of redshifted triangulenium dyes with high fluorescence quantum yields and remarkable long fluorescence lifetime allowing for time-gated cell imaging.

## Introduction

Organic dyes emitting in the red and near-infrared part of the visible spectrum are particularly desired as probes in fluorescence microscopic techniques because the high penetration depth of light in the 650–1000 nm range in tissue allows for non-invasive monitoring of the fluorescent signal in biological material.^1^–^3^ Moreover, a fluorescent signal in this spectral region can be measured with reduced auto-fluorescence from the biological media.^4^–^6^ Several types of fluorescent dyes that effectively emit light in the red and the near-infrared regions have been developed with rhodamine, BODIPY, rylene, and cyanine motifs being the most popular designs.^7^–^10^ In these cases, the fluorophores possess high molar absorptions and fluorescence quantum yields. Several of these fluorophores are commercially available and have found broad application in life-sciences.^11^ Recently, particular interest has been aimed at improving the optical properties and the performance of rhodamine type dyes for applications as probes in for example super-resolution microscopy.^12^–^21^ These dyes possess high brightness and photostability, and have red to near-infrared fluorescence. However, a common feature of these dyes are also short fluorescence lifetimes in the range of 1 to 5 ns. Fluorophores with longer fluorescence lifetimes have advantages in important applications such as; fluorescence lifetime bioassays and imaging (FLIM),^22^,^23^ suppression of auto-fluorescence by time-gated detection,^24^ polarization assays,^25^ and time-gated stimulated emission depletion microscopy (g-STED).^26^–^28^ Despite the obvious applications for fluorescent dyes covering a broad range of fluorescence lifetimes and emission wavelengths only very few dyes with lifetimes above 5 ns are available in the visible range.^22^,^25^,^29^ The lack of such fluorescent dyes may be due to the large focus on high brightness, which eventually leads to short lifetimes,‡
‡The brightness of a fluorophore is given by the product of the molar absorption coefficient at the excitation wavelength and the quantum yield of fluorescence (*ε* × *Φ*). For dyes not undergoing excited state reactions or large structural changes the rate of fluorescence (*k*_rad_) is expected to be proportional to the oscillator strength of the S_0_ → S_1_ transition and thus to *ε*. From this follows that large *ε* values for S_0_ → S_1_ will not only favor high brightness but also fast radiative deactivation and thus short lifetimes see ref. 73 (S. J. Strickler, R. A. Berg, *J. Chem. Phys.*, 1962, **37**, 814–822). as well as the fundamental problem of increasing rates of non-radiative deactivation associated with redshifted fluorescence, as predicted by the energy gap law.^30^

**Chart 1:**
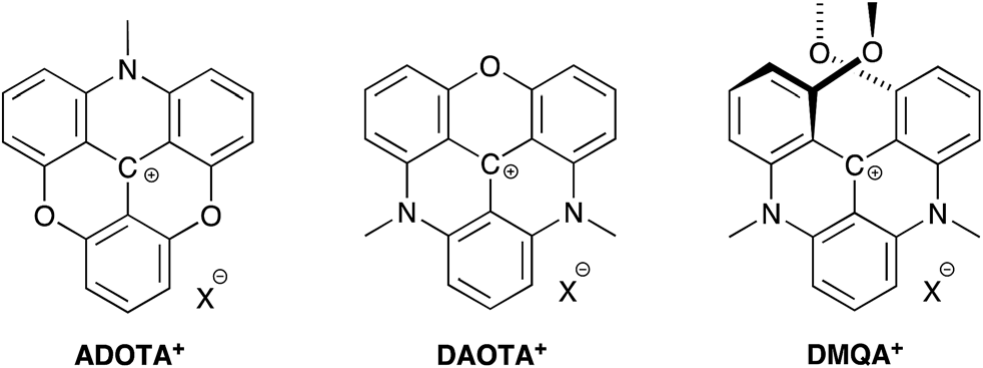
Azadioxatriangulenium (ADOTA^+^), diazaoxatriangulenium (DAOTA^+^) and dimethoxyquinacridinium (DMQA^+^). X^–^ refers to an arbitrary anion.

We have recently shown that the aza/oxa-triangulenium dyes; ADOTA^+^ and DAOTA^+^ (Chart 1) have remarkable low rates of non-radiative deactivation and low susceptibility to quenching by oxygen,^31^ which explains how these organic dyes with fluorescence peaks in the 550 nm–600 nm range, can display unusually long fluorescence lifetimes, close to 20 ns, and high quantum yields (*Φ*_f_ ≈ 0.7–0.8).

The aza/oxa-triangulenium dyes are highly stabilized carbenium ions, with a rigid and planar heterocyclic framework.^32^,^33^ In comparison to rhodamine and cyanine fluorophores the aza/oxa-triangulenium dyes are less bright, as they possess moderate molecular absorption coefficients (*ε*) in the range of 10 000–16 000 M^–1^ cm^–1^.^34^ However, the combination of long fluorescence lifetime, high quantum yields, and chemical stability makes them unique probes in FLIM and time-gated fluorescence imaging,^23^,^24^,^35^,^36^ and for fluorescence lifetime and polarization assays.^36^–^40^ A redshifted congener of DAOTA^+^ is the dimethoxyquinacridinium (DMQA^+^) system (Chart 1). This [4]-helicenium precursor of DAOTA^+^,^32^,^41^ displays absorption and emission in the attractive range from 600 nm–700 nm.^42^,^43^ Lacour and co-workers have intensively functionalized and extended this system demonstrating spectral engineering, circular polarized luminescence (CPL), pH switching, and lipophilic staining of mitochondria.^44^–^51^ While DMQA^+^ displays long fluorescence lifetime in non-polar solvents it display faster non-radiative deactivation than the planer aza/oxa-triangulenium dyes and is practically non-fluorescent in aqueous solutions.^31^,^42^

In the search for new organic fluorophores with long fluorescence lifetimes and emission beyond 600 nm, we turn our attention to modification of the aza/oxa triangulenium dyes, with the expectation that the planarity and rigidity of these systems will keep the rate of non-radiative deactivation low and thus allow long fluorescence lifetime and high quantum yields. In this regard, the DAOTA^+^ dye (Chart 1) is particularly interesting as it display higher chemical stability^32^ and less susceptibility to quenching^40^,^52^ compared to ADOTA^+^. In the present work, structural variations are introduced in the DAOTA^+^ chromophore motif, which redshift the absorption and fluorescence spectra as compared to those of DAOTA^+^ and with only moderate increase in the rate of non-radiative deactivation, thus conserving the desired long fluorescence lifetime.

## Results and discussion

### Chromophore analysis and design

**Chart 2:**
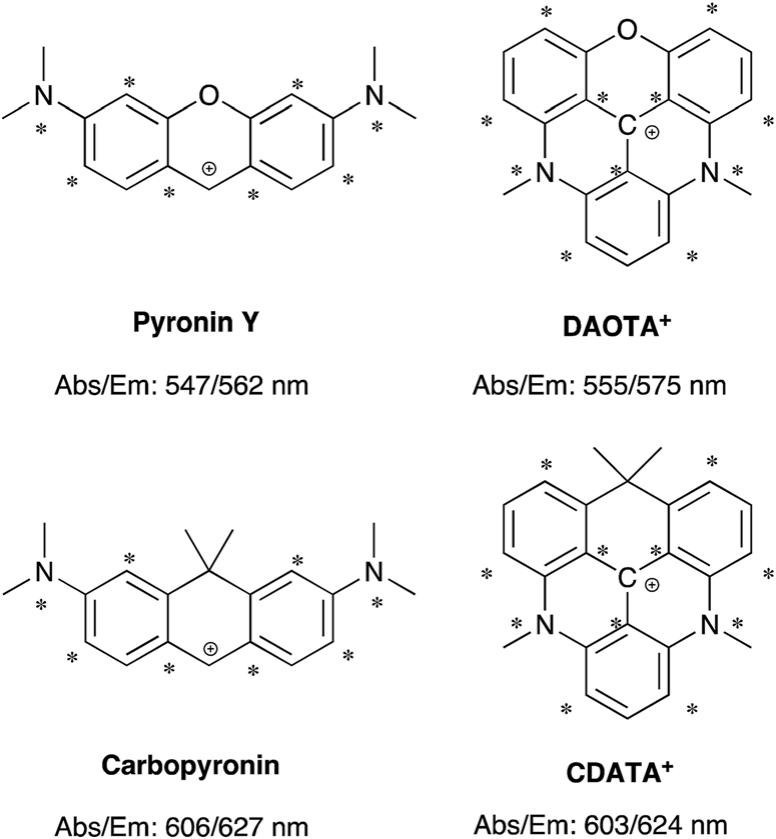
Chromophore analysis of Pyronin Y, carbopyronin, DAOTA^+^, and CDATA^+^. The values of the lowest energy absorption maximum (Abs) and emission maximum (Em) are from this work and ref. 7.

**Scheme 1:**
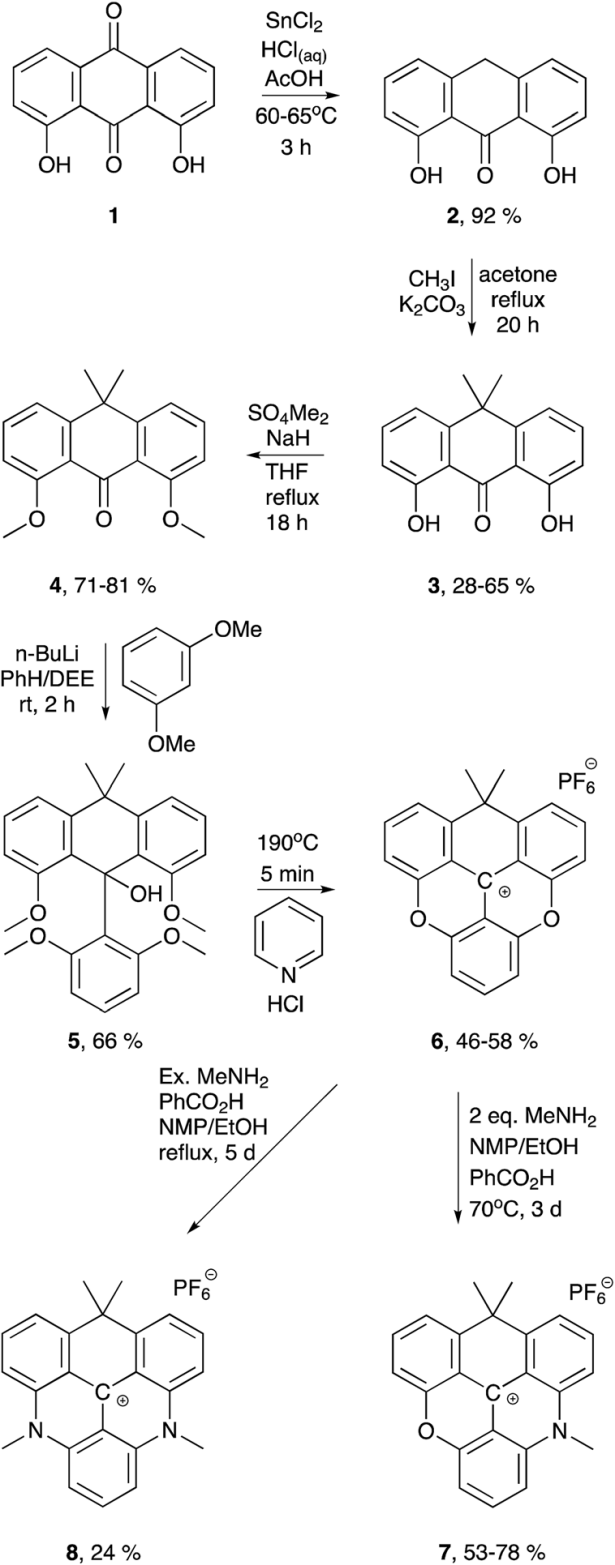
Synthesis of the carbon-bridged dioxa-, azaoxa-, and diazatriangulenium salts (**6**, **7**, and **8**, respectively).

The structural modification of the DAOTA^+^ chromophore is chosen on the basis of Dewar's perturbational molecular orbital (PMO) approach, which provides simple, yet powerful, predictions of the change in energy of optical transitions as function of chromophore modifications.^53^–^56^ Firstly, the DAOTA^+^ chromophore is recognized as belonging to the group of odd alternant π-systems, *i.e*. a conjugated system that is, or can be described as, consisting of an odd number of sp^2^ hybridized carbon atoms. This is illustrated for DAOTA^+^ and the rhodamine analogue Pyronin Y in Chart 2. All other triangulenium and helicenium dyes, including ADOTA^+^ and DMQA^+^ also belong to this group. For such chromophores, the HOMO is distributed on the starred positions and vanishing small at the non-starred positions. Contrary, the distribution of the LUMO is most pronounced at the non-starred positions.§
§For a description of the PMO approach, including starred and non-starred positions see ref. 54 and 55 (Griffiths, J., Colour and Constitution of Organic Molecules, Academic Press, 1976. M. J. S. Dewar and R. C. Dougherty, The PMO Theory of Organic Chemistry, PlenumPress, New York, 1st edn, 1975). For the DAOTA^+^, CDOTA^+^ (**6**), CAOTA^+^ (**7**), and CDATA^+^ (**8**) chromophores this simple qualitative LCAO prediction was confirmed by the Kohn–Sham orbital plots calculated by DFT, see ESI Fig. S29–S32. Since the oxygen bridge in Pyronin Y is connected to non-starred positions it is, to a first approximation, only affecting the LUMO energy. From this, it can be predicted that replacement of the oxygen bridge by less π-electron donating groups, such as the isopropyl bridge, should lower the LUMO energy and thus lead to a redshift in carbopyronin as compared to Pyronin Y,^53^ which has indeed proven to be the case (Chart 2).^7^,^16^,^57^–^59^ In similarity, PMO theory predicts that replacement of the oxygen bridge in DAOTA^+^ by an isopropyl group should lead to the desired redshift. This was further confirmed by TDDFT calculations (see below) and by the spectroscopic data obtained after synthesis of this new carbon-bridged triangulenium dye CDATA^+^ (**8**) and its oxygen containing congeners; CDOTA^+^ (**6**) and CAOTA^+^ (**7**) (see Chart 2 and Scheme 1). The helical DMQA chromophore is fundamentally very similar to the planar DAOTA^+^, but in this case its redshift can be assigned to the twisting of the π-system, which also causes lower radiative rates.^54^ It is noted that PMO analysis also can rationalize the observed effects of both electron donating and withdrawing groups in various positions of DMQA^+^ and related [6]-helicenium systems recently reported by Lacour and co-workers.^45^,^50^

### Synthesis

While the aza and oxa bridges in triangulenium dyes efficiently are introduced by aromatic nucleophilic substitution on methoxysubstituted triarylmethane precursors,^33^,^43^,^60^,^61^ the isopropyl bridge has to be introduced by a stepwise buildup of the triangulenium ring system. For this, we follow a synthetic strategy similar to that reported in the synthesis of sulfur-bridged triangulenium structures.^62^ The syntheses of the carbon-bridged triangulenium salts **6**, **7**, and **8** are outlined in Scheme 1.

The sp^3^-hybridized carbon bridge was introduced into the triangulenium skeleton using tetramethylated dithranol (**4**) as building block. Dithranol was prepared in multigram scale by reduction of 1,8-dihydroxyanthraquinone (**1**) using the procedure reported by Prinz *et al.*^63^ The four methyl groups were introduced sequentially in a two-step procedure with easy purifications. Using conditions of similar reactions,^64^–^66^ **2** was dimethylated in the 10-position giving **3** in moderate yields using two equivalents of methyl iodide and K_2_CO_3_ in refluxing acetone. The hydroxy groups of **3** were easily methylated in a reaction with slight excess of dimethyl sulfate using NaH as base in refluxing THF solution giving **4** in 71–81% yield. The third aromatic unit was introduced by nucleophilic addition of 2,6-dimethoxyphenyllitium using a procedure similar to that reported for related reactions,^62^,^67^ giving **5** in 66% yield. Conversion of **5** into the corresponding carbenium ion would potentially open up a versatile synthetic route for introduction of various substituted aza-bridges into the new triangulenium system in analogy to that described for known triangulenium systems.^43^ However, this carbenium ion proved to be highly unstable and attempts to isolate the carbenium salt were unsuccessful. Thus, a different approach was followed in order to obtain the carbon-bridged triangulenium salts.

The carbon-bridged dioxatriangulenium (CDOTA^+^) hexafluorophosphate salt (**6**) was obtained directly from **5**, by reaction in melted pyridine hydrochloride at 190–200 °C, which effectively converted **5** into **6** in five minutes reaction time. With this stabilized carbenium salt in hand the corresponding azaoxatriangulenium (CAOTA^+^) and diazatriangulenium (CDATA^+^) derivatives (**7** and **8**, respectively) were prepared by aromatic nucleophilic substitution with primary amines following a procedure similar to that reported for synthesis of aza/oxa-triangulenium salts.^32^ The CAOTA^+^ salt **7** was synthesized by reaction of **6** and two equivalents of methylamine and benzoic acid in ethanol/NMP solution. The optimal conditions for preparation of **7** were found when the reaction was carried out in a sealed tube at 70 °C for three days. The presence of benzoic acid elevates the reflux temperature of the reaction mixture,^43^ and it protonates and eases the dissociation of the leuco adduct between methylamine and **6**.^32^ Separation of the traces of **6** and **8** from **7** was found to be very challenging using column chromatography. However, compounds **6** and **8** could be separated from **7** taking advantage of the different reactivity of **6**, **7**, and **8** towards nucleophilic attacks on the center carbon atom. Addition of triethylamine decolorizes a solution of **6**, indicating that this compound forms the leuco adduct with triethylamine. This was found to be useful in effectively separating the traces of **6** from the crude reaction mixture. Addition of excess triethylamine to the reaction mixture followed by addition of diethyl ether precipitated **7** and the trace amounts of **8**, while the leuco adduct between **6** and triethylamine remained in solution. Compound **7** was separated from traces of **8** using a similar strategy followed by recrystallization to give **7** in 53–78% yields. The dimethyl CDATA^+^ dye **8** was obtained in 24% yield by reacting **6** with excess methylamine and benzoic acid in refluxing ethanol/NMP mixture for five days in a closed system.

**Scheme 2:**
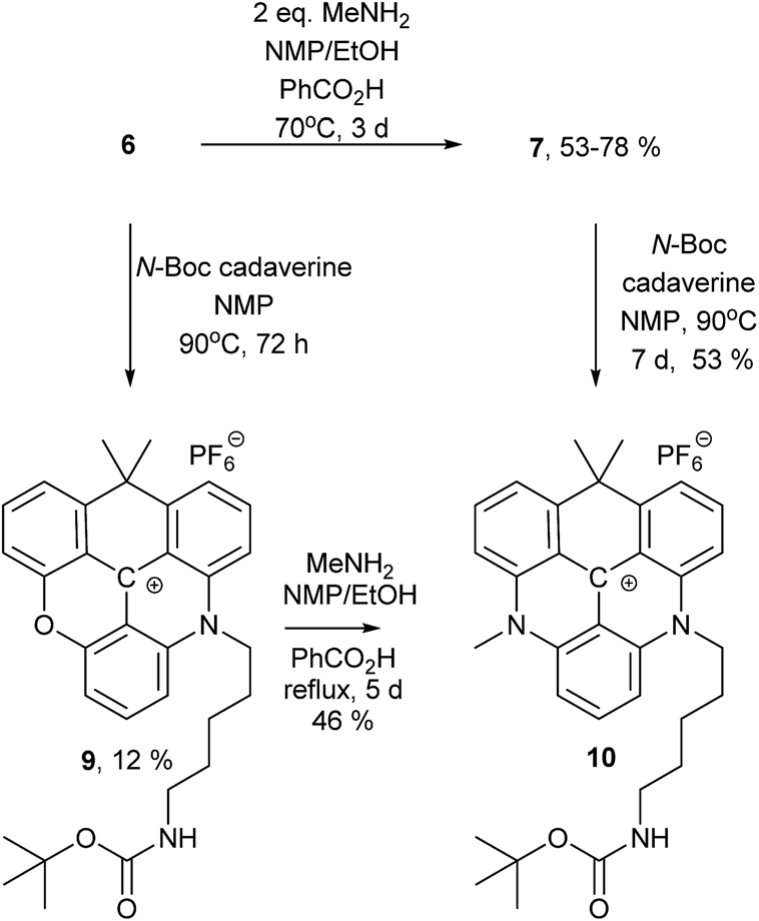
Synthesis of an asymmetric substituted CDATA^+^ dye.

To demonstrate that different amines can be introduced sequentially to yield asymmetric substituted CDATA^+^ dyes containing functional groups, *N*-Boc-cadaverine was reacted with compounds **6** and **7** to give compound **10** (Scheme 2). The results of this investigation showed, that **10** was most effectively obtained *via***7**, with an overall yield up to 42% as compared to 6%, when going *via* compound **9**. A more detailed investigation of the stepwise introduction of groups to yield asymmetric versions of **8** containing functional groups is currently in progress. Functional derivatives **9** and **10** can easily be converted into maleimides and thus allow for efficient bioconjugation of the CAOTA^+^ and CDATA^+^ dyes to cystines.^40^ Reacting **6** with an amino acid, instead of a diamine, would allow for synthesis of activated ester (*e.g.* NHS) derivatives of CAOTA (**7**) and CDATA (**8**) targeting free amines, as previously reported for aza/oxa-triangulenium dyes.^24^,^68^

### Structural effects the carbon bridge in triangulenium ions

The molecular structures of DAOTA^+^, **6**, **7**, and **8** were determined by single crystal X-ray diffraction and by computational methods (see ESI† for details). Selected bond lengths and angles are given in Table S8† and show very good agreement between the calculated structures and those found in the crystal structures. The most interesting observation is the effect of the introduction of the sp^3^-hybridized isopropyl bridge where the C–C bond lengths are found to be significant longer (∼150 pm) than both the C–N and C–O bonds, which are of similar length as the sp^2^ C–C bonds in the triangulenium system (∼140 pm). While longer bonds easily are accommodated in linear ring systems like carbopyronin, 2-dimensional fused ring systems like triangulene are much more constrained. Thus, triangulenes with a central phosphorus atom adopts a non-planar cone shape,^69^–^71^ and attempts to synthesize a triangulenium system with three sulfur bridges failed due to ring tension.^72^

**Fig. 1:**
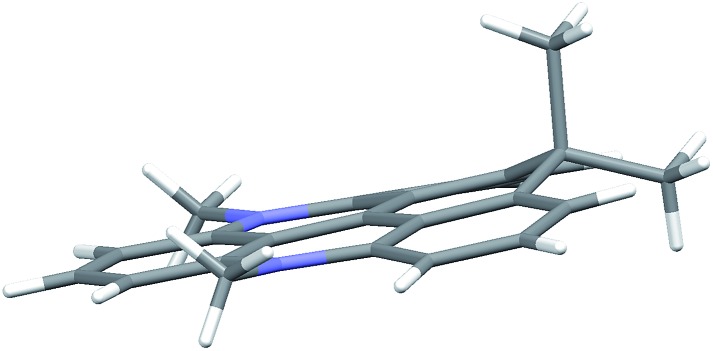
Side view of the non-planer structure found for cationic part of CDATA^+^ (**8**) in the crystal structure of the PF_6_^–^ salt.

The crystal structures of **6** and **7** are found to be planar, yet with expansion of the angle between the rings linked by the isopropyl bridge (125°) (Fig. S38 and Table S8†). For **8**, this tension leads to a non-planar structure with the isopropyl bridge extending ∼50 pm out of plane in the crystal structure (Fig. 1). In the calculated structure, strain is released by bending both nitrogen bridges out of plane (Fig. S39†). Such non-planar conformations could lead to enhanced non-radiative deactivation and thus reduce the fluorescence lifetime and quantum yield.

### Photophysical properties

**Fig. 2:**
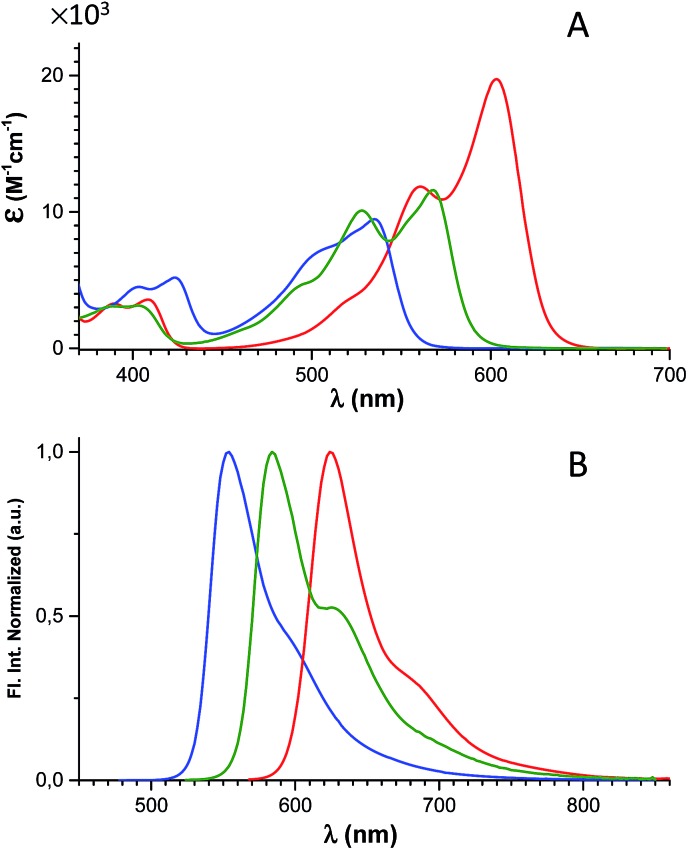
Absorption (A) and normalized fluorescence spectra (B) of **6** (blue), **7** (green), and **8** (red) recorded in CH_2_Cl_2_ solution.

The spectral properties of **6**, **7**, and **8** are shown in Fig. 2 and Table 1 (where data for DAOTA^+^ and DMQA^+^ are included for reference).

**Table 1:** Photophysical properties of **6**, **7**, **8**, DAOTA^+^ and DMQA^+^ in CH_2_Cl_2_ solution^*a*^

Compound	*λ*_abs_, nm	*ε*_max_, M^–1^ cm^–1^	*λ*_f_, nm	*Φ*_f_	*τ*_f_, ns	*k*_rad_, ×10^6^ s^–1^	*k*_nr_, ×10^6^ s^–1^
**6** CDOTA^+^	535	9470	554	0.54	23.1	23	20
**7** CAOTA^+^	568	11 600	584	0.57	17.9	32	24
**8** CDATA^+^	603	19 700	624	0.61	15.8	39	25
DAOTA^+^^*b*^	555	14 700	575	0.76	22.2	34	11
DMQA^+^^*c*^	616	18 100	662	0.17	11.3	15	70

^*a*^Columns list: *λ*_abs_: wavelength at the maximum intensity of the S_0_ → S_1_ transition. *ε*_max_: molar absorption coefficient at *λ*_abs_. *λ*_f_: wavelength at the maximum fluorescence intensity. *Φ*_f_: fluorescence quantum yield. *τ*_f_: fluorescence lifetime. *k*_rad_ = *Φ*_f_/*τ*_f_. *k*_nr_ = (1/*τ*_f_) – *k*_rad_. See ESI experimental details.

^*b*^Data from ref. 31. In an earlier study,^34^ an underestimated quantum yield of 0.44 was reported for DAOTA^+^.

^*c*^Data from ref. 42.

The absorption spectra display a continuous redshift and increase in the molar absorption coefficient (*ε*) as the number of nitrogen bridges is increased going from **6** to **7** and **8**. The fluorescence spectra follow the same trend with a small Stokes shift of ∼500 cm^–1^. Clear vibrational bands are seen on the blue side of the lowest energy peaks in the absorption spectra and are mirrored by vibrational shoulders in the fluorescence spectra. This indicates that all of the absorption spectra above the dip at ∼450 nm can be assigned to the S_0_ → S_1_ transitions and the weaker bands around 400 nm to the S_0_ → S_2_ transitions. This assignment is supported by the results of TDDFT calculations of the optical transitions (see ESI† for details), and by steady-state excitation- and fluorescence anisotropy spectra of **6**, **7**, and **8** measured in glycerol at 0 °C (Fig. S24†). The results of the calculations show that the S_0_ → S_1_ transitions for DAOTA^+^, **6**^+^, **7**^+^, and **8**^+^ involve spatial similar orbitals (Tables S3–S6 and Fig. S29–S32†) and that the orientation of the transition dipole moments in the triangulenium framework are dictated by the relative donor strength of the heteroatom bridges as also found for ADOTA^+^ and DAOTA^+^.^34^ Thus, for CDATA^+^ (**8**), similar to DAOTA^+^, the S_0_ → S_1_ transition is polarized along the axis connecting the two nitrogen bridges, while the S_0_ → S_2_ transition is orthogonal to this, in direction of the isopropyl or oxygen bridge (see Fig. S33†). When comparing CDATA^+^ (**8**) to the reference compound DAOTA^+^, it is found that replacement of the oxygen bridge with an isopropyl bridge conserves the fundamental electronic structure of the chromophore yet leads to a significant redshift of approximately 50 nm in both the absorption and the fluorescence spectra, confirming our design principle guided by Dewar's PMO approach.

Fluorescence quantum yields (*Φ*_f_) and fluorescence lifetimes (*τ*_f_) measured by time-correlated single photon counting (TCSPC) are listed in Table 1. Firstly, it is observed that the new family of carbon-bridged triangulenium dyes all display good quantum yields in the range of 0.5–0.6, which however are lower than those of the aza/oxa-triangulenium dyes ADOTA^+^ and DAOTA^+^ (*Φ*_f_ ≈ 0.7–0.8) under similar conditions.^31^ Most importantly, the desired long fluorescence lifetimes are indeed found for the carbon-bridged triangulenium dyes, 23 ns for **6,** 18 ns for **7** and 15 ns for **8**, respectively. For a more detailed analysis and comparison to DAOTA^+^, the rate constants for fluorescence (*k*_rad_) and non-radiative deactivation (*k*_nr_) were calculated (Table 1). It is found that *k*_rad_ show moderate variations that, as expected,^73^ follows the molar absorption coefficients (*ε*_max_) and the calculated oscillator strengths (Tables S3–S6†). The shorter fluorescence lifetime of CDATA^+^ (**8**) relative to DAOTA^+^ (16 ns *vs.* 22 ns) is mainly a result of a twofold increase of the rate of non-radiative deactivation (*k*_nr_). Within the series of **6**, **7**, and **8,***k*_nr_ only show a small increase with decreasing transition energy (Table 1). This suggests that the increased *k*_nr_ compared to DAOTA^+^, and thus reduced quantum yield and fluorescence lifetime, is related to the isopropyl bridge and not to the redshift (energy gap law). We tentatively assign this increased *k*_nr_ to out-of-plane structural distortions driven by the ring tension in the isopropyl-bridged triangulenes, as discussed above. In this regard it is noted that the non-planar helical DMQA^+^ display significantly higher rates of non-radiative deactivation (Table 1). Despite the increase in non-radiative deactivation, CDATA^+^ (**8**) to a large extend fulfils our objectives: with a quantum yield of 0.6 and a fluorescence lifetime of 16 ns.

### Applications in time-gated fluorescence imaging

**Fig. 3:**
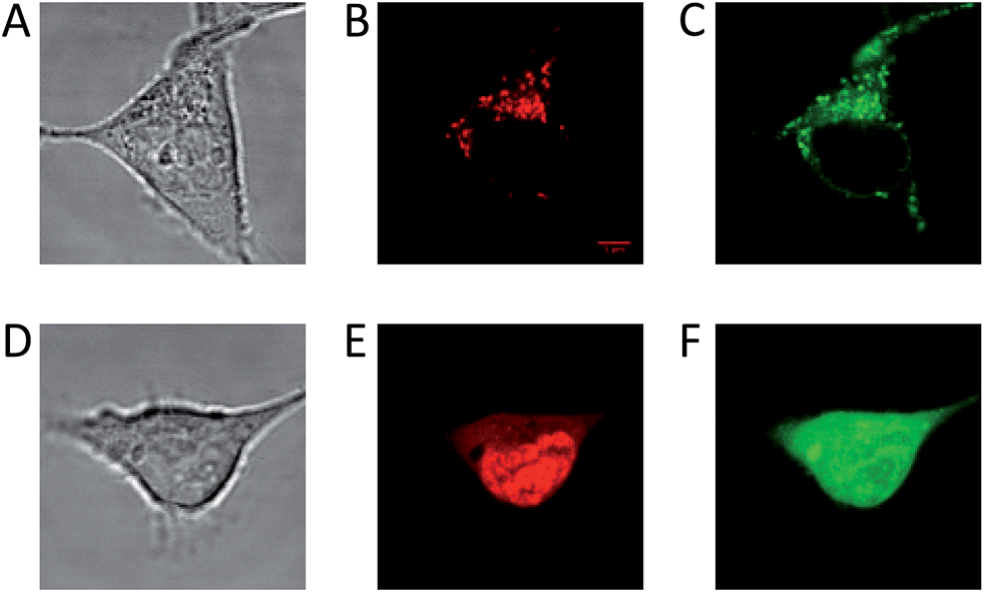
Confocal microscope imaging of stained HEK293 cells (A–C) cell stained with **7** and MitoTracker Green. (A) Transmission image. (B) Fluorescence signal from **7**. (C) Fluorescence signal from MitoTracker Green. (D–F) Cell stained with **8** and Calcein AM. (D) Transmission image. (E) Fluorescence signal from **8**. (F) Fluorescence signal from Calcein AM.

The long fluorescence lifetimes of **6**, **7**, and **8** make these dyes excellent candidates for use in *e.g.* time-gated fluorescence detection.^74^ Initially, Human Embryonic Kidney (HEK293) cells were stained with each of the three fluorophores and imaged by confocal microscopy. The fluorescence signal of **6** was found to be completely quenched in the cellular environment, while the fluorescence signal of **7** and **8** could be monitored (Fig. 3). Compound **7** tends to stain specific parts of the cell (Fig. 3A–C). The fluorescence signal of **7** is detected from some of the same cellular compartments as the fluorescence signal from cells co-stained with MitoTracker Green (for overlap see: Fig. S26†), and thus **7** appears to be partly located in the mitochondria. The fluorescence intensity of **7** was however found to decrease significantly within the recording of few pictures (Fig. S27†), indicating that **7** undergoes relatively fast photobleaching in the cellular environment, and is therefore less suited as probe or stain in microscopy experiments.

**Fig. 4:**
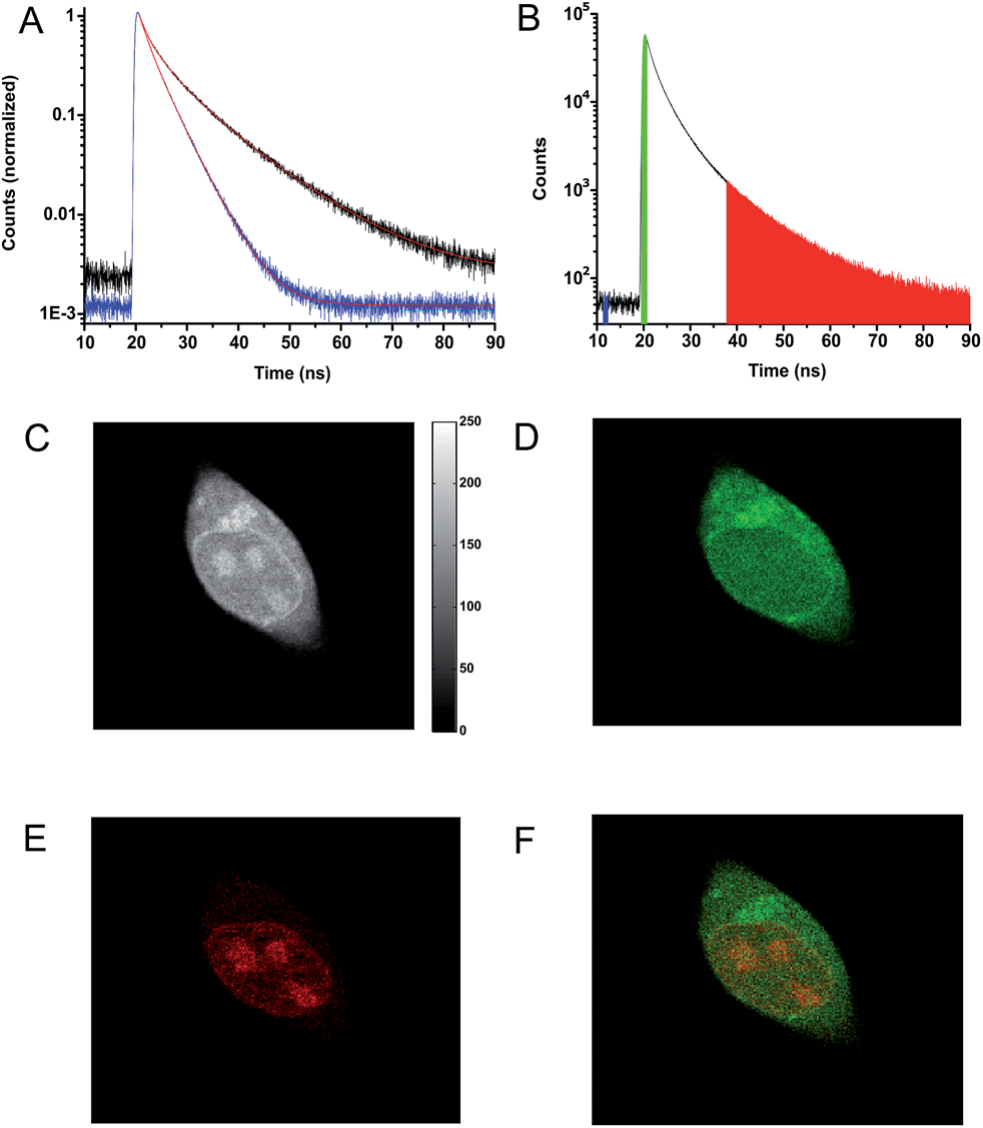
(A) Fluorescence decays for HEK293 cells stained with **8** (black), Calcein Red-Orange (blue). (B) Fluorescence decay for a cell co-stained with **8** and Calcein Red-Orange. The colored areas under the decay curve show photon arrival times used for the construction of the time-resolved images (C–F). (C) Fluorescence intensity image created from all detected photons (19–90 ns). (D) Time-resolved image based on photons from the green range (19–21.5 ns) in the decay shown in (B). (E) Time-resolved image based on the photons from the red range (37.5–90 ns) in the decay shown in (B). (F) Photon arrival time imaging (PArTI) image based on the photons from the green (19–21.5 ns) and red (37.5–90 ns) range in the decay shown in (B).

Fig. 3D–F show a cell stained with CDATA^+^ (**8**), which appears to preferentially stain the nucleus of the cell (Fig. 3E). The cells appeared to be healthy based on their morphology and the co-staining of the cells cytosol with Calcein AM (Fig. 3F) – a commonly fluorescence indicator of cell viability. Moreover, **8** was found to be very photostable (*vide infra*) and the applicability of **8** for time-gated fluorescence microscopy was thus investigated in HEK293 cells. The increased photostability going from **6***via***7** to **8** follows the electron donor strength of the heteroatoms, which from other triangulenium dyes is known to be correlated to cation stability and redox potentials.^32^,^52^ TCSPC experiments on cells stained with **8** showed that the fluorescence decay (Fig. 4A), collected from scanning over a whole cell, was best fitted with three exponential decay components of 1.4 ns, 4.6 ns, and 11.6 ns (Table S2†). The dominating and longest fluorescence lifetime component of 11.6 ns contributes more than half of the overall fluorescence intensity. This fluorescence lifetime component is shorter than the fluorescence lifetime measured in apolar CH_2_Cl_2_ solution (15.8 ns, Table 1), but much longer than the fluorescence lifetime measured in polar DMSO and aqueous solutions (4.9 ns and 3.4 ns, respectively, Table S1†). Thus, the longest fluorescence lifetime component does most likely originate from **8** located in the more lipophilic parts of the cell, while the 4.6 ns component may originate from **8** in more polar environments. This long decay component of **8** in specific cellular environments makes this fluorophore suitable as a probe for time-gated fluorescence microscopy applications. To demonstrate the power of the long fluorescence lifetime of **8**, cells were co-stained with **8** and Calcein Red-Orange. Calcein Red-Orange is a bright BODIPY derived cell-tracker stain,^11^ and was chosen as a co-stain for several reasons. It reports on the cells viability and its fluorescence spectrum overlaps with the fluorescence spectrum of **8** (Fig. S25†). As a result of the latter, the fluorescence signals of the two dyes are hard to resolve by fluorescence microscopy using standard filter combinations, which makes difficult the investigation of fluorescently labeled molecules in the cytosol in the presence of this indicator of cell viability. Calcein Red-Orange displayed biexponential fluorescence decay in the HEK293 cells, with fluorescence lifetime components of 1.8 ns and 4.2 ns (Fig. 4A and Table S2†). The dominant 4.2 ns fluorescence lifetime component, which contributes 78% to the overall fluorescence intensity fits well with the fluorescence lifetime of Calcein Red-Orange measured in DMSO solution (3.7 ns, Table S1†).

**Fig. 5:**
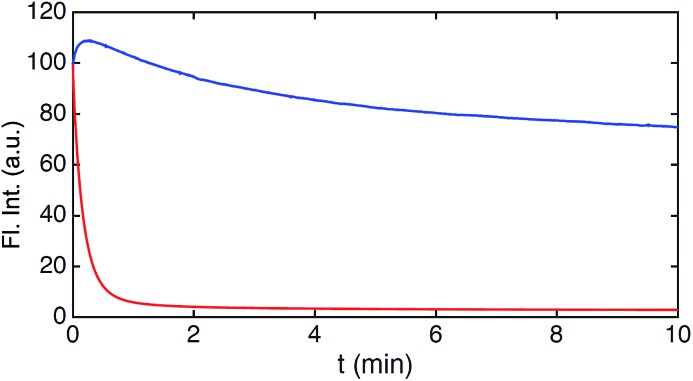
Photobleaching of **8** and Calcein Red-Orange measured in cells. Integrated fluorescence intensity decay upon continuous irradiation (545 nm) of **8** (blue) and Calcein Red-Orange (red).

Fig. 4C–E show the fluorescence signal collected in different time intervals: 19–90 ns, 19–21.5 ns, and 37.5–90 ns (excitation pulse arrives at approximately 19 ns in the measurement window). The photon arrival time imaging (PArTI) is shown in Fig. 4F.^75^ The time-resolved images clearly demonstrate that **8** can be used for time-gated fluorescence detection (and FLIM microscopy) to yield high contrast pictures of cellular environments, even when co-stained with a bright spectrally overlapping dye like Calcein Red-Orange. The photostability of **8** was tested in comparison to that of Calcein Red-Orange. Cells were separately stained with the two dyes and continuously irradiated with intense 545 nm light over a period of 10 min in a wide field microscope setup. The fluorescence intensity photobleaching curves of **8** and Calcein Red-Orange are shown in Fig. 5.

The results of the photobleaching experiments show that **8** is much more photostable than Calcein Red-Orange, and thus should be applicable in many types of fluorescence imaging. The initial increase of the fluorescence intensity within the first 30 seconds of irradiation of **8** in the bleaching experiments is a reproducible feature. The exact reason for the increase is not immediately clear but it is not associated with changes in the fluorescence spectrum (Fig. S28†). A similar feature has been observed in bleaching studies of modified rhodamine 101 analogues and ATTO 647N.^13^,^58^ Overall, these results make **8** very attractive for future fluorescent probe applications, which require red emission, high photostability, and long fluorescence lifetime. The time-gated experiments conducted on cells, using **8**, showed that its application is currently limited to lipophilic targets, as the fluorescence lifetime drops significantly in polar and aqueous environments (Table S1†). However, the introduction of water solubilizing groups may change this limitation, as has been shown *e.g.* for the Alexa dyes,^11^ and the development of this chemistry is in progress.

## Conclusion and perspectives

Three new triangulenium chromophore systems containing a saturated carbon bridge were prepared from a dithranol ring system. Introduction of one or two aza-bridges was achieved *via* nucleophilic replacement of xanthenium type oxygen bridges with primary amines. The procedure allows for formation of asymmetric derivatives with functional side chains (**9** and **10**) that may be used for preparation of bioconjugative derivatives. All three carbon-bridged dye systems (**6**, **7**, and **8**) display redshifted emission compared to the oxygen-bridged analogues, fluorescence quantum yields >50% and long fluorescence lifetimes (>16 ns). Crystal structures and computational results suggest that the saturated carbon bridge introduce ring tension and possible out-of-plane distortions in the triangulenium system, which may be responsible for reduced quantum yields and fluorescence lifetimes. The optical properties demonstrate that it is possible by strategic modifications to redshift the absorption and emission of the triangulenium dyes without significant loss of their unique long fluorescence lifetimes. However, larger structural distortions of triangulenium system are likely to deteriorate the attractive fluorescence properties. While the dioxa (**6**) and azaoxa (**7**) derivatives are sensitive to quenching or bleaching, the diaza derivative CDATA^+^ (**8**) has proven to possess very promising properties as fluorescent stain for time-gated microscopy and FLIM in the red part of the visible spectrum. Future investigations will focus on improving the performance of the CDATA^+^ system in aqueous environment, through the introduction of water solubilizing groups, and to investigate the applications of the dyes in time-resolved imaging and detection.

## Conflicts of interest

The authors declare the following competing financial interests: Bo W. Laursen is associated with the company KU-dyes, which produces and sells dyes (including triangulenium dyes).

## Supplementary Material

Supplementary information

Crystal structure data
