# Supplementary material for: Design, synthesis, and time-gated cell imaging of carbon-bridged triangulenium dyes with long fluorescence lifetime and red emission
Source: Chem Sci. 2018 Feb 23;9(12):3122–30. doi: 10.1039/c8sc00089a (PMC5932597; doi:10.1039/c8sc00089a)
Supplement: Supplementary file 1 [file SC-009-C8SC00089A-s001.pdf]

Electronic Supplementary Material (ESI) for Chemical Science.
This journal is © The Royal Society of Chemistry 2018

SUPPLEMENTARY INFORMATION FOR:

DESIGN, SYNTHESIS AND TIME GATED CELL IMAGING OF CARBON-BRIDGED TRIANGULENIUM DYES WITH LONG FLUORESCENCE LIFETIME AND RED EMISSION

M. Rosenberg,[a]* K. R. Rostgaard,[a] Z. Liao,[a] A. Ø. Madsen,[b] K. L. Martinez,[a] T. Vosch,[a] and B. W. Laursen [a]*

[a] Department of Chemistry, University of Copenhagen, Universitetsparken 5, DK-2100 Copenhagen Ø, Denmark,

[b] Department of Pharmacy, University of Copenhagen, Universitetsparken 2, DK-2100 Copenhagen Ø, Denmark.

E-mails: mrtnrosenberg@gmail.com and bwl@nano.ku.dk

TABLE OF CONTENTS

Experimental Details	2-5
Synthetic procedures	5-11
NMR spectra	11-33
Additional spectroscopic data	34-37
Computational results	38-50
Crystal structures	51-56
References	56

EXPERIMENTAL DETAILS

Spectroscopic Methods: Solvents used for spectroscopic measurements were of highest purity grade and used as received. The UV-vis absorption spectra were recorded on a Perkin-Elmer Lambda 1050 UV/vis/NIR spectrometer double beam spectrometer using the pure solvent as baseline. The spectra were recorded in 1 cm path length cuvettes and 1 nm steps. Molar absorption coefficients were determined according to Lambert-Beers law. The values were determined as the average value of measurements performed on three different stock solutions of each dye in the 10-50 μM ranges. Fluorescence spectra and lifetimes were measured using a FluoroTime 300 (PicoQuant, Berlin, Germany) system. The fluorescence decays were analyzed using the FluoFit software package. The decay data ($J_f(t)$) were fitted by iterative reconvolution with a sum of exponentials:

$$J_f(t) = \sum_i \alpha_i \exp(-t/\tau_i) \qquad (1)$$

In Eq. 1 α_i is the amplitude and τ_i is the fluorescence lifetime of the ith component, respectively. The samples were excited using pulsed solid-state lasers at 460, 510, or 560 nm. The photons were counted at the maximum emission intensity wavelengths of the samples. The instruments response function (IRF) was measured at the excitation wavelength using a dilute scattering suspension of Ludox. For all fluorescence measurements the angle between the excitation and emission polarizers was set to the 54.7^o (magic angle). All fluorescence quantum yields were determined by the relative method.[1] In CH_2Cl_2 solution the samples of **6**, **7**, **8**, and DAOTA were excited by pulsed solid state lasers at 460, 510, and 560 nm and the quantum yield were determined with respect to the values for fluorescein in 0.1 M $NaOH_{(aq)}$ (Φ = 0.89), rhodamine 6G in absolute ethanol (Φ = 0.91), and oxazine 170 in absolute ethanol (Φ = 0.58).[1, 2] The quantum yields of **8** measured in DMSO, water, and PBS solution (Table S1) were measured with excitation at 560 nm using a Xe-lamp excitation source using oxazine 170 in absolute ethanol as reference. The fluorescence measurements were measured in 1 cm path length cuvettes at 90° with respect to the excitation light. For all quantum yield measurements the absorbances were below 0.08 at the maximum of the lowest energy absorption band. After each fluorescence measurement an absorption spectrum were recorded in order to verify that no photo-bleaching of the sample had occurred during the fluorescence measurement. All fluorescence spectra were corrected for the wavelength-dependent sensitivity of the detection from 500-900 nm. The excitation and fluorescence anisotropy spectra of **6**, **7**, and **8** were measured on an Agilent Cary Eclipse spectrometer in glycerol solution at a temperature of 0 ^{o}C. The temperature was obtained by cooling of the cuvette holder with a build-in Peltier element. The temperature of the solution was determined with an external thermometer. The polarized excitation and fluorescence spectra were measured with manually adjustable polarizers. The fluorescence anisotropies were measured with excitation of **6**, **7**, and **8** at 490 nm, 525 nm, and 550 nm, respectively. The excitation anisotropies were measured at the fluorescence signal at 580 nm, 610 nm, and 650 nm for **6**, **7**, and **8**, respectively.

Time-Gated Imaging: The time-resolved imaging was performed using a home-built set-up for single molecule microscopy and spectroscopy.[3-5] Briefly, a 560 nm pulsed laser from PicoQuant operated at 10 MHz was used as excitation source (linearly polarized), and the power used for the imaging and recording of spectra was 0.4 W/cm^2 for measurements on cells stained with Calcein Red-Orange and co-stained cells, 3.4 W/cm^2 for measurements on cells stained with **8**, and 170 W/cm^2 for measurements on non-stained cells (based on assuming that the laser is focused into a diffraction limited spot). The light was focused on the sample by an oil objective (Olympus UPLFLN

100 X). The excitation light was separated from the emission by a long-pass filter (BLP01-561R-25, Semrock). The dimensions are 50 x 50 μm for all the time-resolved images shown in this work, and the resolution is 200 x 200 pixels. The integration time at each pixel was 4 ms. The signal from the Avalanche Photo Diode (APD, Perkin-Elmer CD3226) was recorded in FIFO mode by a time-correlated single photon counting card (SPC-830, Becker & Hickl). A home-written MATLab routine was used to create the time-resolved images from the FIFO data file.[5] The latter allows us to create images from any time interval in the decay curve.[5] No additional polarization elements were added in the detection part. The reported decay times were determined by summing up all the photons in the whole image and the decay curves were fitted by a tail-fitting procedure. The IRF of the APD is several hundreds of picoseconds and due to the intensity depend IRF response of the APD, caution should be taken in interpreting time constants below 1 ns. Emission spectra were recorded with a spectrograph and cooled CCD camera (Princeton Instruments SPEC-100:100B/LN_eXcelon CCD camera, SP 2356 spectrometer).

Cell Staining and Confocal Imaging: HEK293 cells (Sigma-Aldrich) were maintained in Dulbecco's modified Eagle medium (DMEM/F12 + Glutamax-1, Gibco) with 10 % fetal bovine serum (FBS, Gibco) at 37 °C, 5 % CO_2, and more than 95 % humidity. For live cell staining, cells were incubated 20 min at 37 °C with the following dyes as indicated for each experiment: 10 μM **7**, 10 μM **8**, 110 nM MitoTracker Green (Life Technologies), 3 μM Calcein AM (Life Technologies) or 5 μM Calcein Red-Orange AM (Life Technologies). For co-staining experiments, dyes were incubated together. After staining, cells were washed twice with DMEM/HEPES (Gibco) and imaged immediately by confocal microscopy or time-gated fluorescence detection. Confocal imaging was performed on an inverted confocal laser-scanning microscope (Leica TCS SP5) using a 63x magnification, water-immersion objective with numerical aperture of 1.2. The fluorophores were excited and emission collected by a photomultiplier tube at ex543:em563-700 (**7**), ex594:em614-750 (**8** and Calcein Red-Orange AM) and ex488:em508–540 (MitoTracker Green and Calcein AM), and transmission light was collected at ex633:trans623–643. Images were obtained with a pinhole of one Airy unit, and a pixel size of 68x68 nm^2. Photobleaching experiments were performed on HEK293 cells stained with either Calcein Red-Orange AM or **8** as described above. Collections of the cells were irradiated at 545 nm using a 100 W Hg-lamp excitation source in a home-built wide field fluorescence microscope setup. The emission spectra were recorded through a 590 nm long-pass filter using a spectrometer from Ocean Optics in combination with the SpectraSuite Software (Ocean Optics). The photobleaching decays constructed from the integrated fluorescence intensity between 600-850 nm measured each 100 millisecond for 10 minutes.

Computational Methods: All calculations were performed with the Gaussian 09 suite of programs,[6] and all structures were geometry optimized at the B3LYP/6-31+G(d) level, as this method has been used for geometry optimizations of similar compounds.[7-9] The optimizations of the compounds were performed by inclusion of the polarizable continuum model (PCM) to simulate CH_2Cl_2 as solvent. The results of frequency calculations performed on the same level verified the calculated structures as minimum energy structures. Vertical excitation energies were calculated using the TDDFT methodology at the TD-PCM-CAM-B3LYP/6-311+G(2d,p)//PCM-B3LYP/6-31+G(d) level, using linear response non-equilibrium solvation by PCM to simulate CH_2Cl_2 as solvent. The CAM-B3LYP functional in combination with the 6-311+G(2d,p) basis set was chosen, as it has shown to perform well for calculations of excitation energies of organic dyes.[10, 11]

Synthetic Methods and Materials: All chemicals and solvents were used as received, unless otherwise stated. The compound: 4,8-dimethyl-4,8-diaza-12-oxatriangulenium hexafluorophosphate (DAOTA) was prepared as previously described.[12] Tetrahydrofuran was purified and dried by distillation from a Na / benzophenone couple and benzene was dried over solid sodium for at least 24 h. Thin layer chromatography (TLC) was carried out on commercially available pre-coated plates (silica 60) with fluorescence indicator. All melting points are uncorrected. Chromatographic purifications were performed on silica gel (SiO_2) with a pore-size of 60Å and a particle size of 40-63 μm. High-resolution mass spectra were recorded on an ESP-MALDI-FT-ICR instrument equipped with a 7 Tesla magnet (the instrument was calibrated using sodium trifluoroacetate cluster ions prior to acquiring the spectra) or a MicrOTOF-QII-system using ESP (calibrated using formic acid). ^{1}H and ^{13}C NMR spectra were acquired on a 500 MHz instrument equipped with a (non-inverse) cryoprobe. ^{19}F NMR spectra were acquired on a 300 or 500 MHz instrument. All chemical shift values in ^{1}H and ^{13}C NMR spectra are referenced to the residual solvent peak ($CDCl_3$ δH = 7.26 ppm, δC = 77.16 ppm. DMSO-d_6 δH = 2.50 ppm, δC= 39.52 ppm, CD_2Cl_2 δH = 5.32, δC = 54.00, CD_3CN δH = 1.94 ppm, δC = 1.32 ppm). Chemical shift values in the ^{19}F NMR spectra are referenced to trifluoroacetic acid (δF= –76.55 ppm), which was present in a sealed lock-tube inside the NMR sample during the acquisition. Elemental analyses were done at the University of Copenhagen, Department of Chemistry, Elemental Analysis Laboratory, Universitetsparken 5, 2100 Copenhagen, Denmark.

Crystallography: Ellipsoid plots and crystallographic details are given in Figures S40-S43 and Table S7-S8. Crystals of **6** and **7** were grown from CH_2Cl_2/2-propanol mixtures through slow evaporation of the CH_2Cl_2, while crystals of **8** and DAOTA were grown from CH_3CN/2-propanol mixtures through slow partial evaporation of the solvents. Single crystals of each compound were selected by using a polarizing microscope. The diffraction data were collected on a Bruker D8 Venture diffractometer using micro-focused Mo K-alpha radiation. The temperature was maintained at 122 K by using a liquid nitrogen low temperature device from Cryo Industries of America. The data reduction was performed in the Bruker Apex suite of programs. The structures of **6**, **8**, and DAOTA were obtained by direct methods using SHELXS-97 and refined by full-matrix least-squares methods against F^2 using the SHELXL program from within the Olex2 interface. The positions and anisotropic displacement parameters were refined for all non-hydrogen atoms.

The quality of the diffraction data for **7** is not optimal. Despite having tested numerous crystals from different batches, the crystal quality appears to be inherently bad. The structure was obtained using intrinsic phasing in SHELXT. The low resolution and high internal R-value, combined with the large unit cell (Z'=4 in the space group P-1) has forced to use a simple model consisting of isotropic displacement parameters on all atoms apart from the PF_6 group. The data are sufficient to confirm the molecular structure and give the overall molecular geometry. The low data quality is reflected in the high R-factors.

The crystals show pseudo-symmetry, and can be approximated as belonging to the monoclinic space group P21/n (Z´=1, V=1913 A^3). However, as revealed by additional peaks in the residual electron density maps, and by analysis of synthetic precession images, the structure is better described as a structure with four molecules and four PF_6 units in the asymmetric unit in the space group P-1 (Z = 4, V=3828 A^3). The PF_6 units fit perfectly to the symmetry of the smaller cell, whereas the triangulenium units show deviations in the positions. To keep the data to parameter ratio reasonable, the triangulenium units were refined using isotropic displacement parameters, and the

hydrogen atoms were placed and constrained based on geometrical criteria. The crystallographic data in the form of cif files have been deposited with the Cambridge Crystallographic Data Centre (CCDC) under the deposition numbers CCDC 1557677-1557680.

SYNTHETIC PROCEDURES

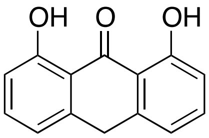

1,8-dihydroxyantracen-9(10*H*)-one (2):[13] In a 2 L 3-necked flask **1** (17 g, 71 mmol) was suspended in glacial acetic acid (1700 mL). A solution of $SnCl_2 \bullet 2\ H_2O$ (54.8 g 243 mmol) in $HCl_{(aq)}$ (37 %, 150 mL) was added in one portion. The mixture was heated to 60-65 °C and maintained at these conditions for 3 h. The hot solution was allowed to reach ambient temperatures and then poured into water (2000 mL). The yellow precipitate was filtered off, washed with water (1000 mL), and then with a saturated $NaHCO_{3(aq)}$ solution (200 mL). The orange solid was dissolved in CH_2Cl_2 through the filter and the organic phase was washed consecutive times with saturated $NaHCO_{3(aq)}$ until no gas evolved. The organic phase was then washed with water (2 x 150 mL). The organic phase was dried over Na_2SO_4 and the solvent was removed by evaporation in vacuum. The orange powder was dried in vacuum (< 1 mbar). Yield: 14.6 g (91 %). The orange product can be recrystallized from toluene to yield planar orange crystals. ^{1}H NMR (500 MHz, $CDCl_3$) δ 12.29 (s, 2H), 7.49 (t, *J* = 7.9 Hz, 2H), 6.96-6.86 (m, 4H), 4.36 (s, 2H). ^{13}C NMR (126 MHz, $CDCl_3$) δ 194.3, 163.1, 142.1, 136.4, 118.8, 116.0, 115.7, 33.0. Melting point. 179-180 ^{o}C (litt.[14]: 176-181 ^{o}C). MS (GC-MS): Calcd for $C_{14}H_{10}O_3^+$ [M^+]: 226.1. Found 226.1. Elem. Anal. Calcd for $C_{14}H_{10}O_3$: C, 74.33; H, 4.46. Found: C, 74.23, H, 4.09. R_f (toluene) = 0.66.

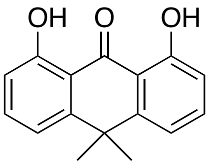

1,8-dihydroxy-10,10-dimethylanthracen-9(10*H*)-one (3): Acetone (500 mL) was heated to refluxing conditions under N_2 atmosphere for 2 h. The acetone was cooled to ambient temperatures and then **2** (10.2 g, 45.1 mmol), methyl iodide (5.7 mL, 92 mmol), and dry K_2CO_3 (13 g, 94 mmol) were added. The mixture was heated to refluxing conditions under N_2 atmosphere for 20 h. The reaction mixture was allowed to reach ambient temperatures and then filtered. The solid was washed with acetone (2 x 50 mL) and discarded. The combined acetone solutions were poured into an equal volume of $HCl_{(aq)}$ solution (1 M). The mixture was stirred for 30 min and then extracted with CH_2Cl_2 (2 x 150 mL) in order to extract the product. The solvents of the organic phase were removed by evaporation in vacuum. The brown sticky material was dissolved in CH_2Cl_2 and dried over Na_2SO_4. The solution was filtered through a pad of silica (R_f (CH_2Cl_2) = 0.92). The solvent was removed by evaporation in vacuum and the yellow material was recrystallized twice from heptane to yield yellow needle crystals*. Yield: 3.7 g (32 %)**. ^{1}H NMR (500 MHz, DMSO-*d*$_6$) δ 12.45 (bs, 2H), 7.67 (t, *J* = 8.1 Hz, 2H), 7.36 (dd, *J* = 7.9, 1.0 Hz, 2H), 6.91 (dd, *J* = 8.4, 0.9 Hz, 2H), 1.66 (s, 6H). ^{13}C NMR (126 MHz, DMSO-*d*$_6$) δ 192.7, 162.0, 152.6, 137.3, 118.1, 115.0, 113.4,

38.6, 33.5. HRMS (ESP): Calcd for $C_{16}H_{14}O_3Na^+$ [M+Na$^+$]: 277.0835. Found: 277.0842. Melting point: 113-115 °C (103-105 °C)*. Elem. Anal. Calcd for $C_{16}H_{14}O_3$: C, 75.58; H, 5.55. Found: C, 75.66; H, 5.43. R_f (Ethyl acetate:heptane, 1:10) = 0.41. *Note 1: The compound was crystallized in two different forms with different melting points. The lowest melting crystal form yielded orange crystals upon recrystallization. **Note 2: The yield was improved to 50-65 % when **2** was recrystallized from toluene before use.

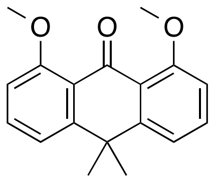

1,8-dimethoxy-10,10-dimethylanthracen-9(10*H*)-one (4): A dry 250 mL 3-necked flask equipped with a condenser, septum, and a stopper was flushed with N_2 gas. Sodium hydride (60 % suspension in mineral oil, 1.4 g, 35 mmol) was added, and the flask was cooled to 0 °C on an ice bath. Compound **3** (3.50 g, 13.8 mmol) was dissolved in dry THF (20 mL) and was slowly added via a syringe through the septum into the flask. When no bubbles evolved from the mixture, dimethyl sulfate (3.6 mL, 38 mmol) was added to the reaction mixture through the septum via a syringe. The mixture was heated to refluxing conditions while under N_2 atmosphere. After 20 h, the reaction mixture was allowed to reach ambient temperatures and 2-propanol (20 mL) was added, and when no gas evolved water (50 mL) was added. The mixture was stirred for 30 min. The organic solvents were removed by evaporation in vacuum. Additional water (200 mL) was added to the precipitate. The solid material was filtered off and dried by suction. The solid material was dissolved in CH_2Cl_2 (50 mL) and dried over $MgSO_4$. The crude product was isolated by evaporation of the solvent in vacuum and the material was recrystallized from ethyl acetate yielding bright yellow, square crystals. Yield: 2.74 g (70 %). ^{1}H NMR (500 MHz, DMSO-d_6) δ 7.49 (t, J = 8.1 Hz, 2H), 7.29 (dd, J = 7.9, 0.9 Hz, 2H), 7.04 (dd, J = 8.4, 0.8 Hz, 2H), 3.81 (s, 6H), 1.58 (s, 6H). ^{13}C NMR (126 MHz, DMSO-d_6) δ 183.8, 157.7, 150.4, 132.8, 123.7, 116.4, 110.4, 55.7, 38.6, 31.7. Melting point: 165-167 °C. HRMS (MALDI-TOF, dithranol matrix): Calcd for $C_{18}H_{18}O_3H^+$ [M+H$^+$]: 283.1334. Found: 283.1328. Elem. Anal. Calcd for $C_{18}H_{18}O_3$: C, 76.57; H, 6.43. Found: C, 76.26; H, 6.24. R_f (CH_3OH:CH_2Cl_2, 1:99) = 0.56.

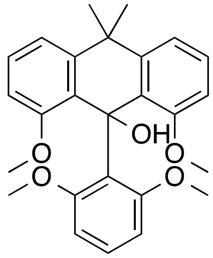

9-(2,6-dimethoxyphenyl)-1,8-dimethoxy-10,10-dimethyl-9,10-dihydroanthracen-9-ol (5): In a 100 mL dry flask fitted with a septum, which was evacuated under inert atmosphere, dry benzene (7.5 mL), diethyl ether (7.5 mL), 1,3-dimethoxybenzene (3 mL, 23 mmol), TMEDA (3.6 mL, 24 mmol) were added and cooled to 0 °C on an ice bath. *n*-Butyl lithium (1.6 M, 15 mL, 24 mmol) was added upon gentle stirring. The ice bath was removed and a white precipitate formed. The suspension was stirred at ambient temperatures for 3 h at ambient temperatures. Compound **4** (2.03 g, 7.2 mmol) dissolved in dry benzene (50 mL) was added via a syringe through the septum. The

mixture was stirred at ambient temperatures for 1 h. The reaction was quenched by addition of 2-propanol (20 mL) followed by addition of water (50 mL). The organic solvents were removed by evaporation in the vacuum. The white solid was filtered off and washed with water (2 x 50 mL). The white solid was suspended in heptane (75 mL) and stirred. The suspension was cooled to –25 °C for 1 h. The cold white suspension was filtered and the white solid was dried by suction. The white solid was recrystallized from absolute ethanol (–25 °C). The white crystalline powder yielded 2 g (66 %). ^{1}H NMR (500 MHz, CD_2Cl_2) δ 7.43 (s, 1H), 7.22 (t, J = 8.0 Hz, 2H), 7.16 (dd, J = 8.1, 1.3 Hz, 2H), 7.04 (t, J = 8.3 Hz, 1H), 6.71–6.67 (m, 3H), 6.36 (dd, J = 8.2, 1.2 Hz, 1H), 4.10 (s, 3H), 3.46 (s, 6H), 2.87 (s, 3H), 1.83 (s, 3H), 1.60 (s, 3H). ^{13}C NMR (126 MHz, CD_2Cl_2) δ 160.8(+), 157.7(+), 156.8(+), 144.3(+), 131.2(+), 127.7(–), 126.8(–), 119.5(–), 110.8(–), 110.0(–), 105.5(–), 100.6(+), 73.4(+), 57.7(–), 57.2(–), 56.2(–), 38.3(–), 37.8(–), 32.2(–). Melting point: 263 °C (decomp.). HRMS (MALDI-TOF, dithranol matrix): Calcd for $C_{26}H_{27}O_4^+$ [M^+–H_2O]: 403.1904. Found: 403.1905. Elem. Anal. Calcd for $C_{26}H_{28}O_5$: C, 74.26; H, 6.71. Found: C, 73.98; H, 6.73. R_f (CH_3OH:CH_2Cl_2, 1:99) = 0.52.

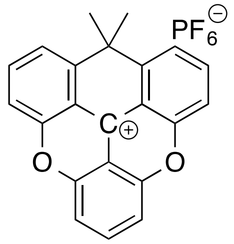

12,12-dimethylbenzo[8,1]isochromeno[3,4,5,6-*klmn*]xanthenium hexafluorophosphate, [CDOTA$^+$] (6): Pyridine hydrochloride (17 g, 147 mmol) was melted and heated to 190-200 °C. When the compound crystallized onto the inside of the flask, a heat gun was used to melt it back into the flask (this was done to remove excess pyridine). The latter was performed three times, and then compound **5** (817 mg, 1.94 mmol) was added in three portions upon vigorous stirring. Gas evolved each time. When the third addition was complete the mixture was kept at 180-190 °C for 5 min. The heat was removed and the mixture was allowed to reach ambient temperatures. The sticky mixture was suspended in water (50 mL), and the slurry was poured into an acidified $KPF_{6(aq)}$ solution (0.2 M, 150 mL, acidified to pH 1-2 using 2 M $HCl_{(aq)}$ solution) upon stirring. The orange precipitate was filtered off and dried by suction. The orange material was dissolved in CH_2Cl_2, dried over $MgSO_4$, filtered, and the solvent was removed by evaporation in vacuum. The sticky orange-brown material was dissolved in a minor volume CH_2Cl_2 and then poured into stirred diethyl ether (400 mL). The resulting orange-red precipitate was allowed to form and then filtered off. The material was recrystallized from ethanol/acetonitrile mixture yielding shinny red crystals. Yield: 510 mg (58 %). ^{1}H NMR (500 MHz, CD_3CN) δ 8.58 (d, J = 8.5 Hz, 1H), 8.35 (t, J = 8.2 Hz, 2H), 8.09 (d, J = 7.8 Hz, 2H), 7.87 (t, J = 8.1 Hz, 4H), 1.92 (s, 6H). ^{13}C NMR (126 MHz, CD_3CN) δ 156.9, 154.5, 151.8, 151.0, 144.3, 142.8, 126.2, 117.2, 113.1, 112.5, 110.7, 42.4, 34.5. ^{19}F (282 MHz, DMSO-d_6) δ –70.97 (d, J = 706.3 Hz). HRMS (MALDI-TOF, dithranol matrix): Calcd for $C_{22}H_{15}O_2^+$ [M^+]: 311.1067. Found: 311.1067. Elem. Anal. Calcd for $C_{22}H_{15}F_6O_2P$: C, 57.91; H, 3.31. Found: C, 58.05; H, 3.02. R_f (CH_2Cl_2:CH_3OH, 96:4) = 0.31.

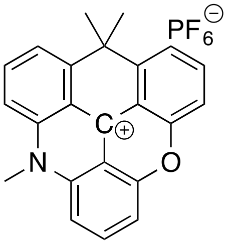

8,12,12-trimethyl-8,12-dihydro-3a^{2}H-benzo[8,1]isochromeno[3,4,5,6-*klmn*]acridinium hexafluorophosphate, [CAOTA$^+$] (7): Compound **6** (125 mg, 0.27 mmol) and benzoic acid (75 mg, 0.61 mmol) were dissolved in NMP (2 mL) in a sealable tube equipped with a small magnetic stir bar. Cold methylamine in absolute ethanol (33 wt%, 0.07 mL, 0.56 mmol) was added using a micropipette and the tube was quickly and tightly sealed. The mixture was heated to 70 ^oC for three days. The heat was removed and mixture was slowly cooled to ambient temperatures and the tube was carefully opened. The reaction mixture was poured into an acidified $KPF_{6(aq)}$ solution (0.2 M, 150 mL, acidified to pH 1-2 using 2 M $HCl_{(aq)}$ solution) upon stirring. The violet product was extracted with CH_2Cl_2 (2 x 50 mL) and the solvent was removed by evaporation in vacuum. The violet solid was washed with diethyl ether (2 x 25 mL). The solid was dissolved in a minor volume CH_2Cl_2 and an equal volume triethylamine was added upon stirring. The resulting slurry mixture was stirred for 30 min at ambient temperatures and then poured into diethyl ether (400 mL) upon stirring. The precipitate was collected by filtration and washed with diethyl ether (2 x 50 mL). The precipitate was dissolved in DMSO and an equal volume of $KOH_{(aq)}$ solution (2 M) was added. This resulting slightly blue solution was extracted with diethyl ether. The diethyl ether was removed in vacuum and the resulting sticky product was dissolved in a minor volume CH_2Cl_2 and then stirred with a two-fold volume of acidified $KPF_{6(aq)}$ solution (0.2 M, acidified to pH 1-2 using 1 M $HCl_{(aq)}$). The resulting purple solution organic solution was separated and washed twice with acidified $KPF_{6(aq)}$ solution (0.2 M, acidified to pH 1-2 using 1 M $HCl_{(aq)}$). The organic solvent was removed in vacuum. The purple solid was dissolved in a minor volume CH_2Cl_2 and a twofold volume of 2-propanol was carefully layered on top to crystallize the product as violet crystals by slow evaporation of CH_2Cl_2. Yield: 100 mg (79 %). ^1H NMR (500 MHz, CD_3CN) δ 8.40 (t, J = 8.5 Hz, 1H), 8.35-8.30 (m, 1H), 8.15 (d, J = 9.0 Hz, 1H), 8.07 (d, J = 7.5 Hz, 1H), 8.03 (t, J = 8.1 Hz, 1H), 7.93 (d, J = 9.0 Hz, 1H), 7.88 (d, J = 7.7 Hz, 1H), 7.62 (d, J = 8.1 Hz, 1H), 7.58 (d, J = 8.4 Hz, 1H), 4.38 (s, 3H), 1.89 (s, 6H). ^{13}C NMR (126 MHz, CD_3CN) δ 154.5(+), 153.9(+), 149.9(+), 149.6(+), 144.2(+), 143.0(+), 141.8(+), 140.7(–), 139.7(–), 138.7(–), 125.2(–), 124.8(–), 116.22(–), 116.2(–), 115.4(+), 113.5(+), 113.4(+), 110.6(–), 109.4(–), 41.5(+), 38.1(–), 35.6(–). ^{19}F NMR (282 MHz, DMSO-d_6) δ –70.96 (d, J = 706.6 Hz). HRMS (MALDI-TOF, dithranol matrix): Calcd for $C_{23}H_{18}NO^+$ [M^+]: 324.1383. Found: 324.1383. Elem. Anal. Calcd for $C_{23}H_{18}F_6NOP$: C, 58.86; H, 3.87; N, 2.98. Found: C, 58.77; H, 3.70; N, 3.06 %. R_f (CH_2Cl_2:CH_3OH, 95:5) = 0.45.

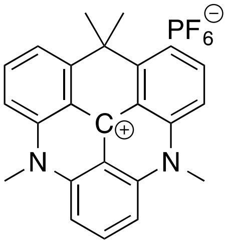

4,8,12,12-tetramethyl-8,12-dihydrobenzo[8,1]isoquinolino[3,4,5,6-*klmn*]acridinium hexafluorophosphate, [CDATA$^+$] (8): Compound **6** (355 mg, 0.778 mmol) and benzoic acid (6 g, 49 mmol) were dissolved in NMP (6 mL) in a 100 mL round-bottomed flask fitted with a condenser equipped with a balloon on top. Cold methylamine in absolute ethanol (33 wt%, 6 ml, 48 mmol) was added and the mixture was heated to refluxing conditions for 5 days. The reaction mixture was allowed to reach ambient temperatures and then poured into an acidified $KPF_{6(aq)}$ solution (0.2 M, 200 mL, acidified to pH 1-2 using a 2 M $HCl_{(aq)}$ solution). The blue precipitate was allowed to form, filtered off, washed with water (100 mL), and dried by suction. The blue material was then washed with diethyl ether (200 mL). The solid material was dissolved in CH_2Cl_2 (100 mL) through the filter and this solution was dried over $MgSO_4$, filtered, and poured into stirred diethyl ether (500 mL). The blue precipitate was allowed to form and then filtered off. The material was purified twice by column chromatography (CH_2Cl_2:CH_3OH, 95:5) followed by recrystallization from ethanol/CH_3CN yielding shinny violet needle crystals. Yield: 90 mg (24 %). ^{1}H NMR (500 MHz, CD_3CN) δ 8.28 (t, J = 8.6 Hz, 1H), 8.08 (t, J = 8.2 Hz, 2H), 7.91–7.85 (m, 1H), 7.50 (d, J = 8.6 Hz, 2H), 4.11 (s, 6H), 1.87 (s, 6H). ^{13}C NMR (126 MHz, CD_3CN) δ 149.1(+), 143.3(+), 142.5(+), 142.0(+), 139.4(–), 137.3(–), 123.4(–), 116.4(+), 115.19(+), 115.1(–), 106.0(–), 41.1(+), 37.4(–), 36.5(–). ^{19}F NMR (282 MHz, DMSO-d_6) δ –70.96 (d, J = 706.3 Hz). HRMS (MALDI-TOF, dithranol matrix): Calcd for $C_{23}H_{18}NO^+$ [M^+]: 337.1699. Found: 337.1700. Elem. Anal. Calcd for $C_{23}H_{21}F_6N_2P$: C, 59.75; H, 4.39; N, 5.81. Found: C, 59.67; H, 4.64; N, 5.92 %. R_f (CH_2Cl_2:CH_3OH, 95:5) = 0.47.

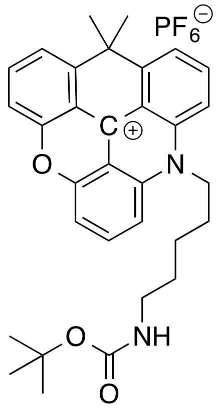

8-(5-((*tert*-butoxycarbonyl)amino)pentyl)-12,12-dimethyl-8,12-dihydrobenzo[8,1]isochromeno[3,4,5,6-*klmn*]acridinium hexafluorophosphate (9): Compound 6 (486 mg, 1.07 mmol) was suspended in NMP (7 mL) and *N*-Boc-cadaverine (0.66 mL, 3.2 mmol) was added. The flask was fitted with a condenser and the reaction mixture was heated on an oil bath set to 90 ^{o}C. After 3 days, the heat was removed and the mixture was allowed to reach ambient temperatures and then poured into water (250 mL) upon stirring. The slurry was filtered and the dark precipitate was washed consecutive times with water. The sticky precipitate was dissolved in CH_2Cl_2 (150 mL) and washed with water (2 x 25 mL). The organic phase was dried over Na_2SO_4. The solvent of the organic phase was reduced to a minimum in vacuum and this solution was poured into heptane (200 mL) upon stirring. The precipitate was filtered off, re-dissolved in a minimum volume CH_2Cl_2 and poured into heptane (150 mL) upon stirring. The precipitate was filtered off and purified by column chromatography (acetone:CH_2Cl_2, 20:80). Yield: 80 mg (12 %). ^{1}H NMR (500 MHz, CD_3CN) δ 8.40-8.35 (m, 1H), 8.33-8.28 (m, 1H), 8.15 (d, J = 9.0 Hz, 1H), 8.06 (d, J = 7.5 Hz, 1H), 8.02 (t, J = 8.1 Hz, 1H), 7.94 (d, J = 9.0 Hz, 1H), 7.87 (dd, J = 7.9, 0.9 Hz, 1H), 7.60 (d, J = 8.0 Hz, 1H), 7.57 (dd, J = 8.3, 0.9

Hz, 1H), 5.32 (s, 1H), 4.86 (t, *J* = 8.4 Hz, 2H), 3.09 (q, *J* = 6.5 Hz, 2H), 2.11-2.02 (m, 2H), 1.89 (s, 6H), 1.72-1.54 (m, 4H), 1.40 (s, 9H). ^{13}C NMR (126 MHz, CD_3CN) δ 157.1(+), 154.4(+), 153.9(+), 150.0(+), 149.6(+), 144.2(+), 142.2(+), 141.0(+), 140.8(–), 139.8(–), 138.7(–), 125.2(–), 124.8(–), 116.2(–), 116.1(–), 115.6(+), 113.7(+), 113.3(+), 110.5(–), 109.5(–), 79.1(+), 50.5(+), 41.5(+), 40.4(+), 35.6(–), 30.5(+), 28.7(–), 27.2(+), 24.2(+).^{19}F NMR (470 MHz, CD_3CN) δ –70.96 (d, *J* = 706.9 Hz). Elem. Anal. Calcd for $C_{32}H_{35}F_6N_2O_3P$: C, 60.00; H, 5.51; N, 4.37. Found: C, 59.97; H, 5.58; N, 4.20 %. HRMS (MALDI-TOF, dithranol matrix): Calcd for $C_{32}H_{35}N_2O_3^+$ [M$^+$]: 495.2642. Found: 495.2653. R_f(acetone:CH_2Cl_2, 20:80) = 0.36.

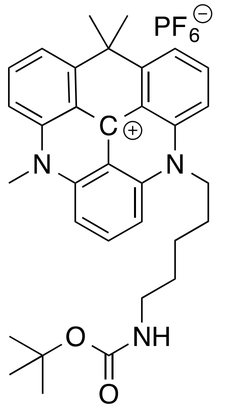

4-(5-((*tert*-butoxycarbonyl)amino)pentyl)-8,12,12-trimethyl-8,12-dihydrobenzo[8,1]isoquinolino[3,4,5,6-*klmn*]acridinium hexafluorophosphate (10): Method 1: Compound 7 (180 mg, 0.384 mmol) was dissolved in NMP (7 mL). *N*-Boc-cadaverine (2.5 mL, 12 mmol) was added and the mixture was heated to 90 °C on an oil bath for 7 days. The blue reaction mixture was allowed to reach ambient temperatures and then poured into diethyl ether (400 mL) upon vigorous stirring. After 1 min, heptane (100 mL) was added to the stirred mixture. The mixture was decanted through a Büchner funnel. The blue material was a mixture of viscous oil and a solid. The material was purified by column chromatography (acetone:CH_2Cl_2, 20:80, dry loading on Celite). The material was dissolved in a minor volume CH_2Cl_2, filtered, and the solvent was removed by evaporation in vacuum. The resulting blue material was washed with diethyl ether and dried. Yield: 134 mg (53 %).

Method 2: Compound **9** (55 mg, 0.086 mmol) and benzoic acid (600 mg, 4.91 mmol) were dissolved in NMP (5 ml). Methylamine (0.6 mL, 33 wt% in absolute ethanol, 5 mmol) was added and the flask was fitted with a condenser equipped with a balloon on top, and the mixture was heated to gentle refluxing conditions on an oil bath set to 90 °C. After 5 days, the heat was removed and the mixture was allowed to reach ambient temperatures. The mixture was poured into $KPF_{6(aq)}$ solution (0.2 M, 150 mL) upon vigorous stirring. The mixture was stirred for 15 min and then filtered. The solid was dissolved in CH_2Cl_2 through the filter and the organic phase was dried over Na_2SO_4, filtered, and the solvent was removed by evaporation in vacuum. The sticky blue material was purified by column chromatography (acetone:CH_2Cl_2, 20:80, dry loading on Celite). The material was dissolved in minor volume CH_2Cl_2, filtered, and the solvent was removed by evaporation in vacuum. The resulting blue material was washed with diethyl ether and dried. Yield: 26 mg (46 %). ^{1}H NMR (500 MHz, CD_3CN) δ 8.27 (t, *J* = 8.6 Hz, 1H), 8.11-8.04 (m, 2H), 7.91-7.84 (m, 4H), 7.52 (d, *J* = 8.7 Hz, 1H), 7.49 (d, *J* = 8.6 Hz, 1H), 5.33 (s, 1H), 4.58 (s, 2H), 4.10 (s, 3H), 3.11 (q, *J* = 6.3 Hz, 2H), 2.04-1.97 (m, 2H), 1.86 (s, 6H), 1.68-1.57 (m, 4H), 1.41 (s, 9H). ^{13}C NMR

(126 MHz, CD_3CN) δ 157.1(+), 149.2(+), 149.1(+), 143.4(+), 142.6(+), 141.9(+), 141.6(+), 141.1(+), 139.5(–), 137.4(–), 137.3(–), 123.5(–), 123.4(–), 116.6(+), 115.3(+), 115.1(+), 115.1(–), 114.9(–), 106.0(–), 105.8(–), 79.1(+), 49.7(+), 41.1(+), 40.5(+), 37.5(–), 36.5(–), 30.5(+), 28.7(–), 26.1(+), 24.22(+). ^{19}F NMR (470 MHz, CD_3CN) δ –70.96 (d, J = 705.8 Hz). HRMS (MALDI-TOF, dithranol matrix): Calcd for $C_{33}H_{38}N_3O_2^+$ [M^+]: 508.2959. Found: 508.2964. R_f (acetone:CH_2Cl_2, 20:80) = 0.32.

NMR SPECTRA

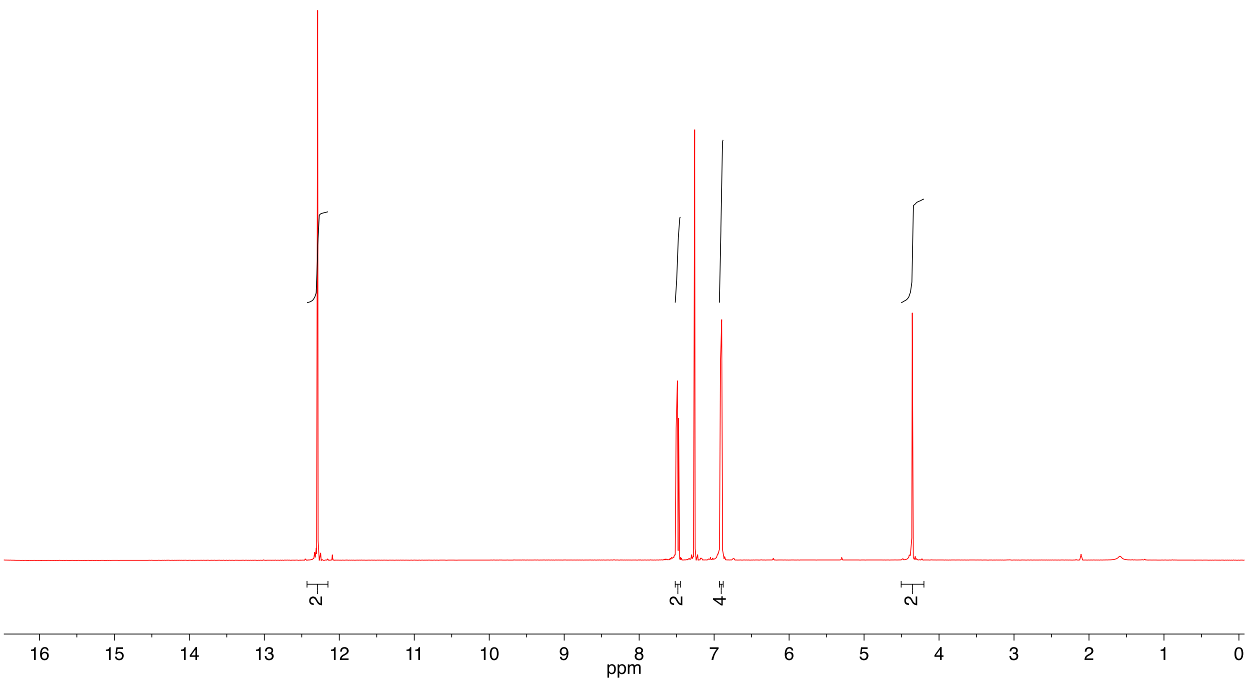

Figure S1. ^1H NMR spectrum of **2** recorded in $CDCl_3$ (500 MHz).

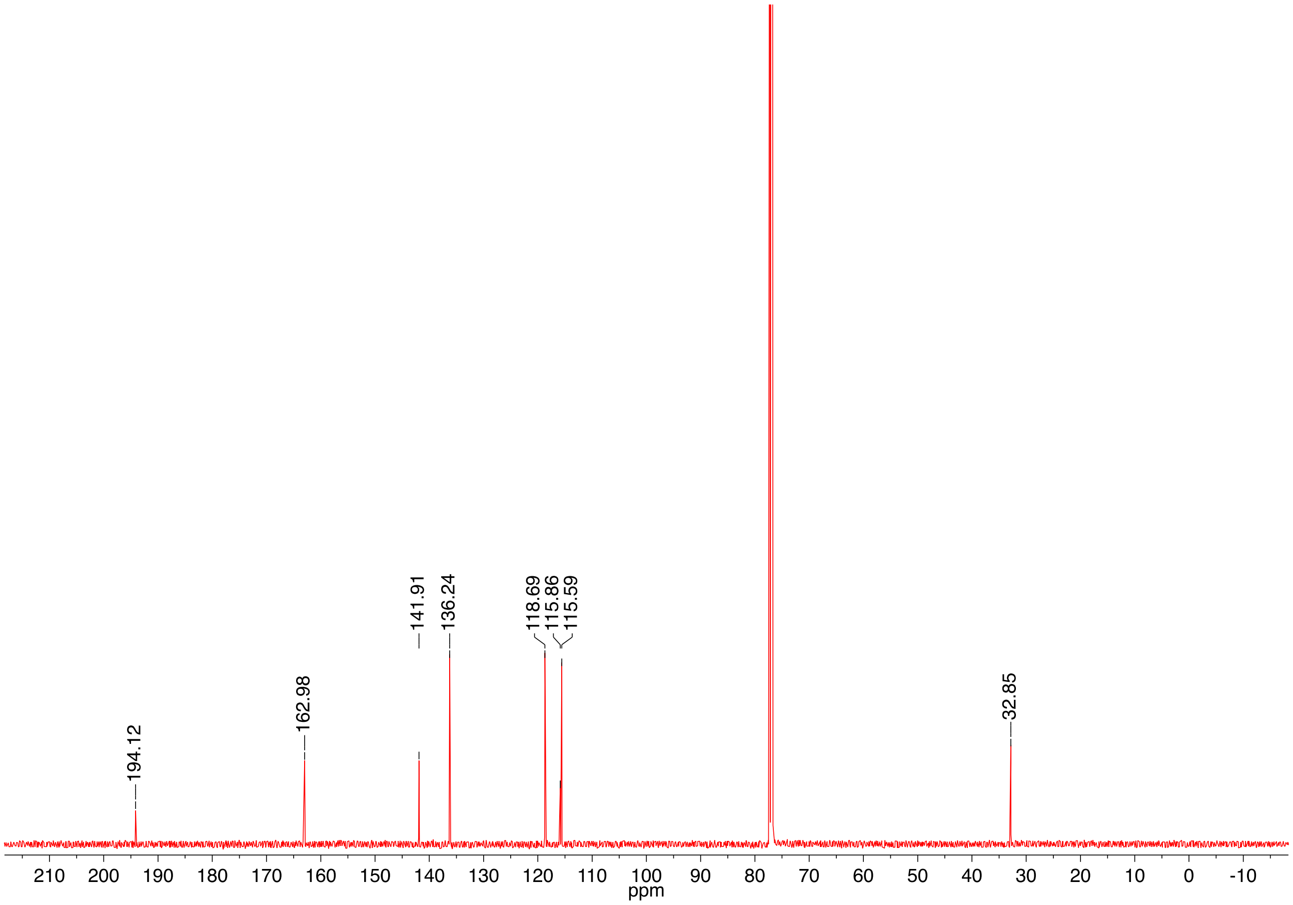

Figure S2. ^{13}C NMR spectrum of **2** recorded in $CDCl_3$ (126 MHz).

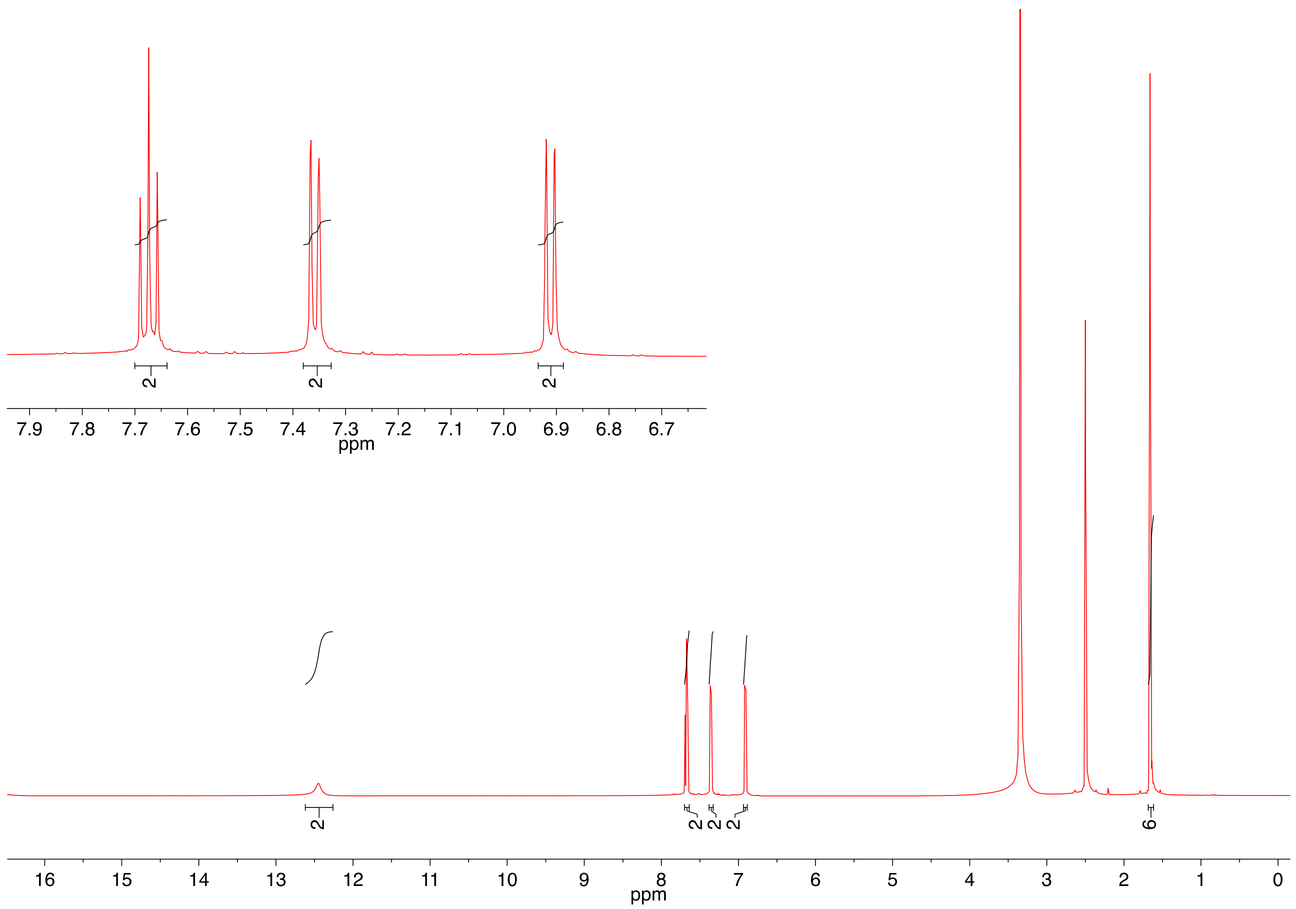

Figure S3. ^{1}H NMR spectrum of **3** recorded in DMSO-d_6 (500 MHz).

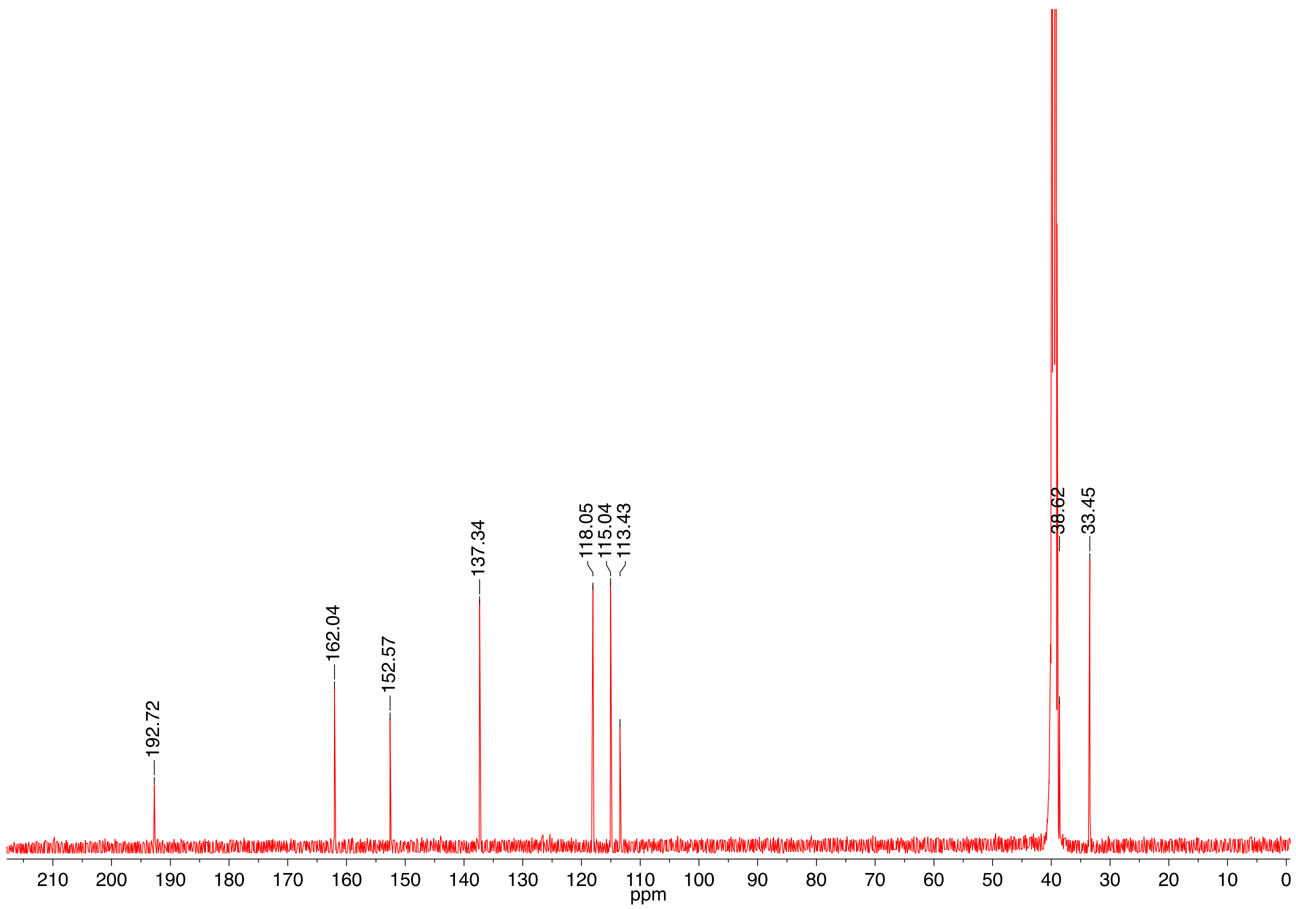

Figure S4. ^{13}C NMR spectrum of **3** recorded in DMSO-d_6 (126 MHz).

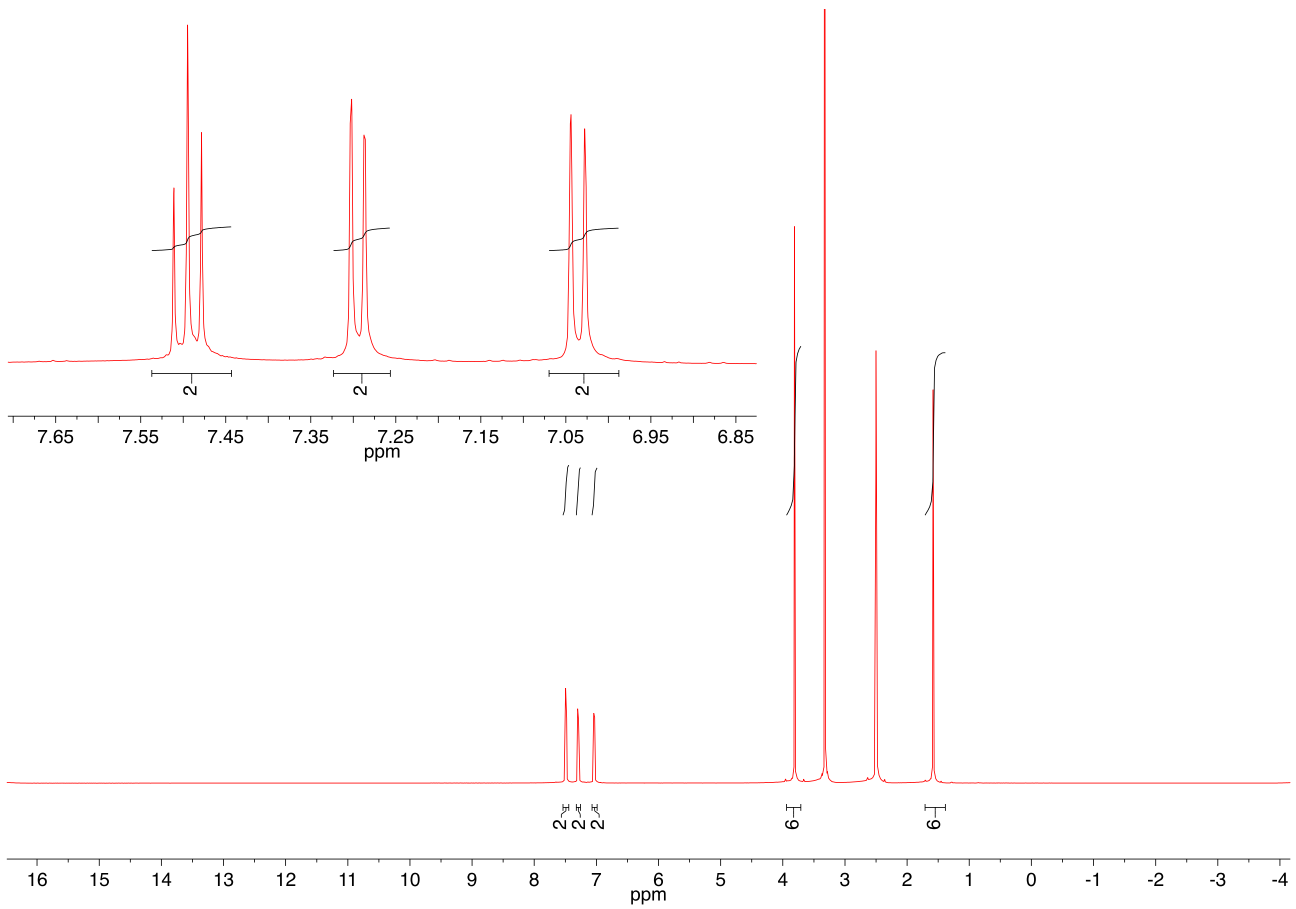

Figure S5. ^{1}H NMR spectrum of **4** recorded in DMSO-d_6 (500 MHz).

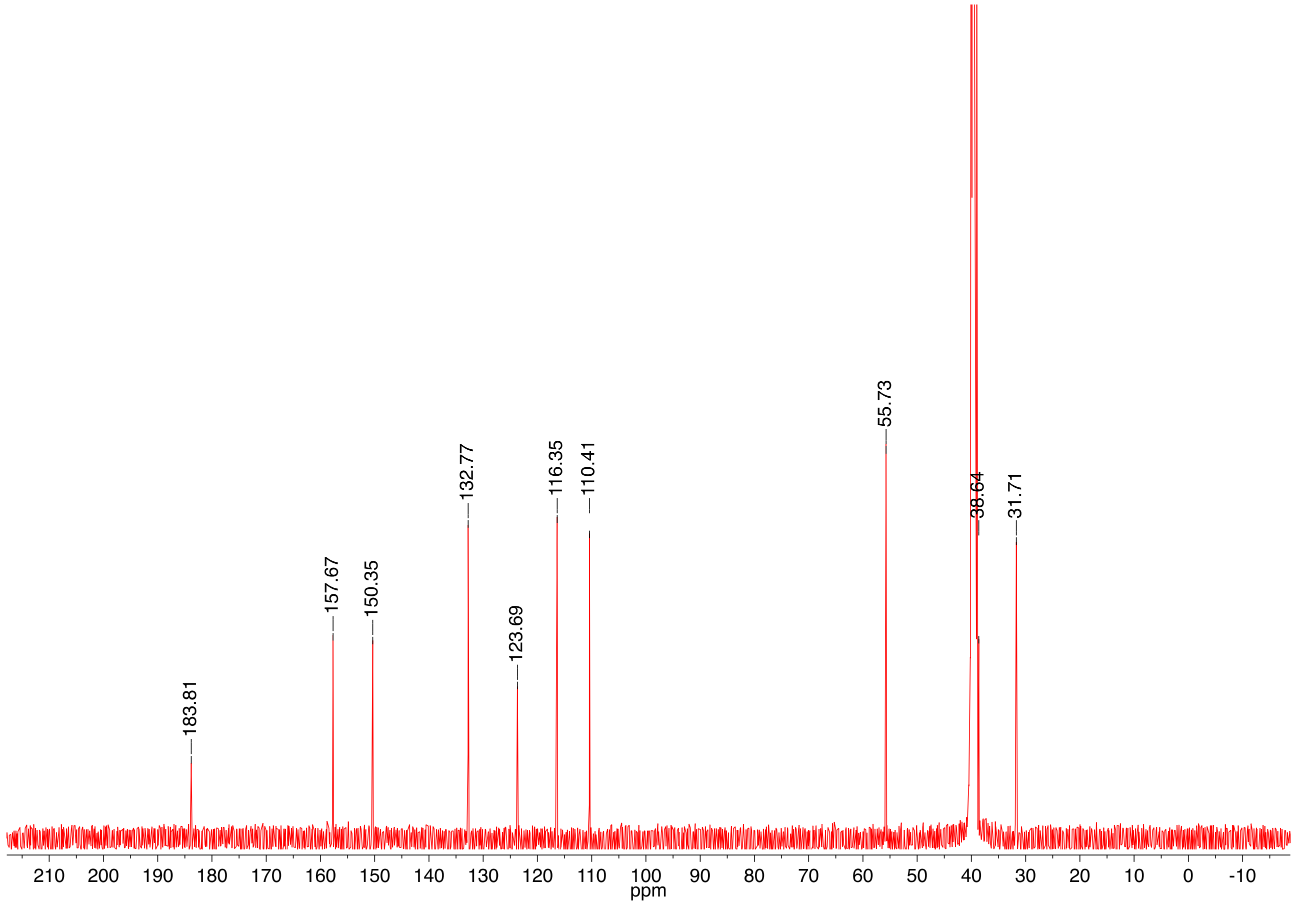

Figure S6. ^{13}C NMR spectrum of **4** recorded in DMSO-d_6 (126 MHz).

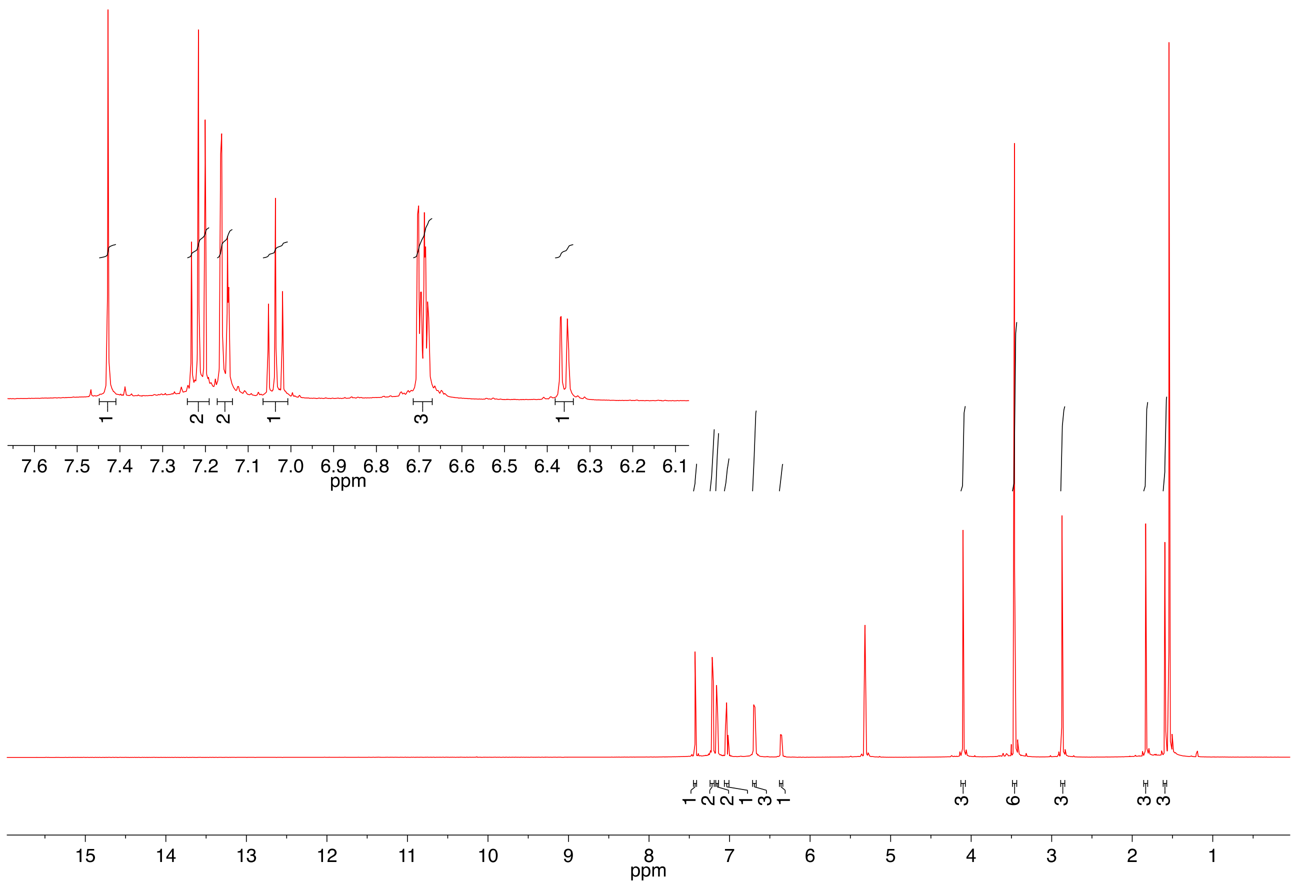

Figure S7. ^{1}H NMR spectrum of **5** recorded in CD_2Cl_2 (500 MHz).

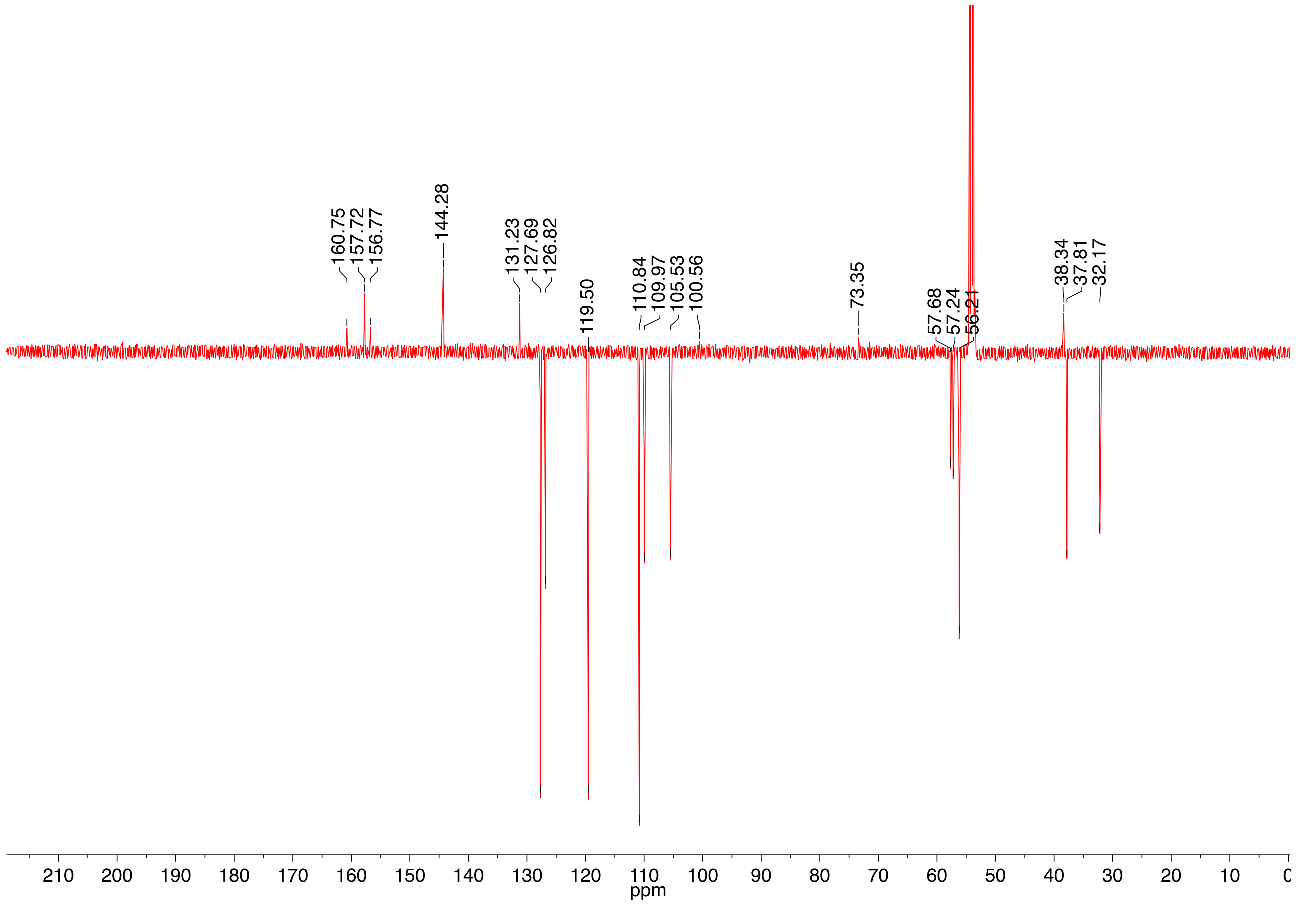

Figure S8. ^{13}C APT NMR spectrum of **5** recorded in CD_2Cl_2 (126 MHz).

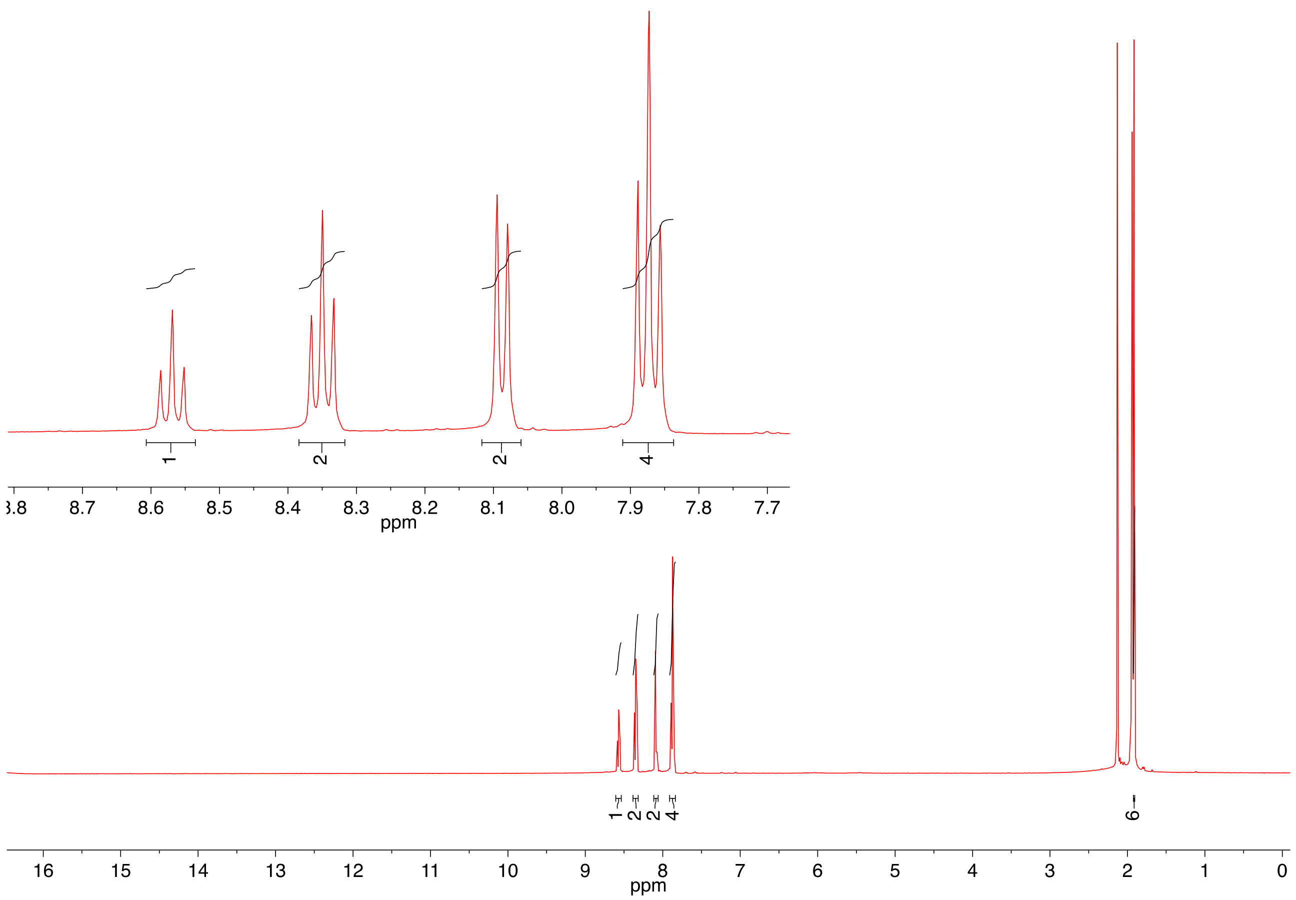

Figure S9. ^{1}H NMR spectrum of **6** recorded in CD_3CN (500 MHz).

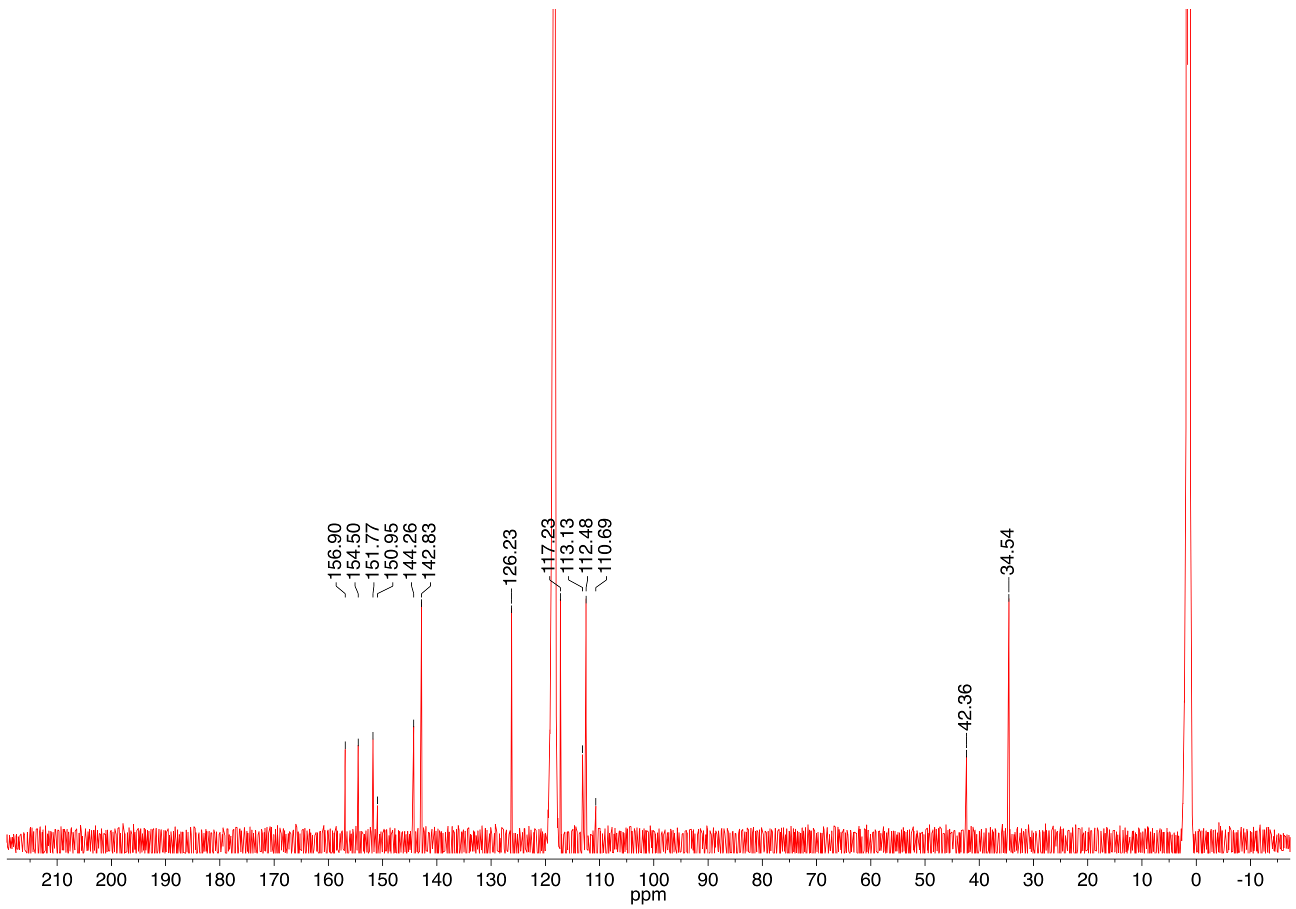

Figure S10. ^{13}C NMR spectrum of **6** recorded in CD_3CN (126 MHz).

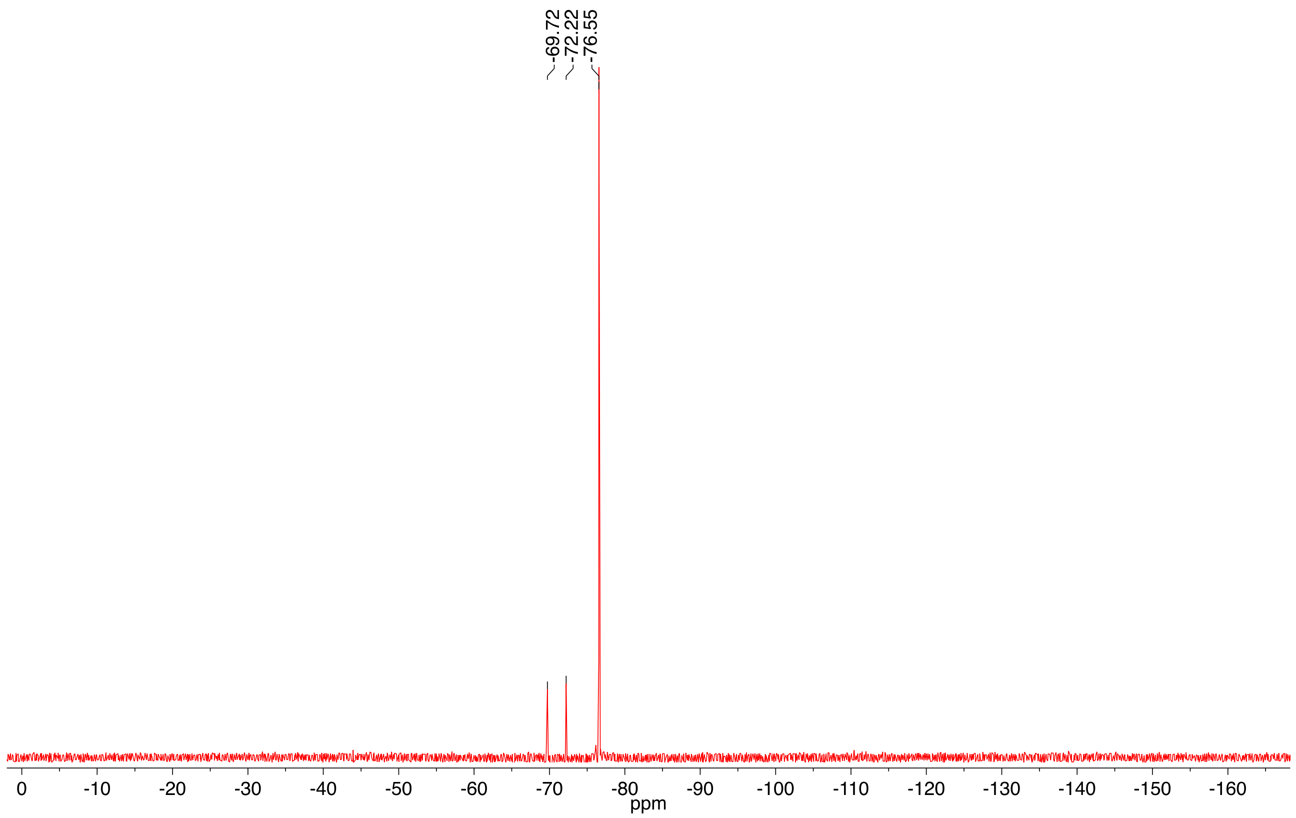

Figure S11. ^{19}F NMR spectrum of **6** recorded in DMSO-d_6 (282 MHz, CF_3COOH at –76.55 ppm as reference).

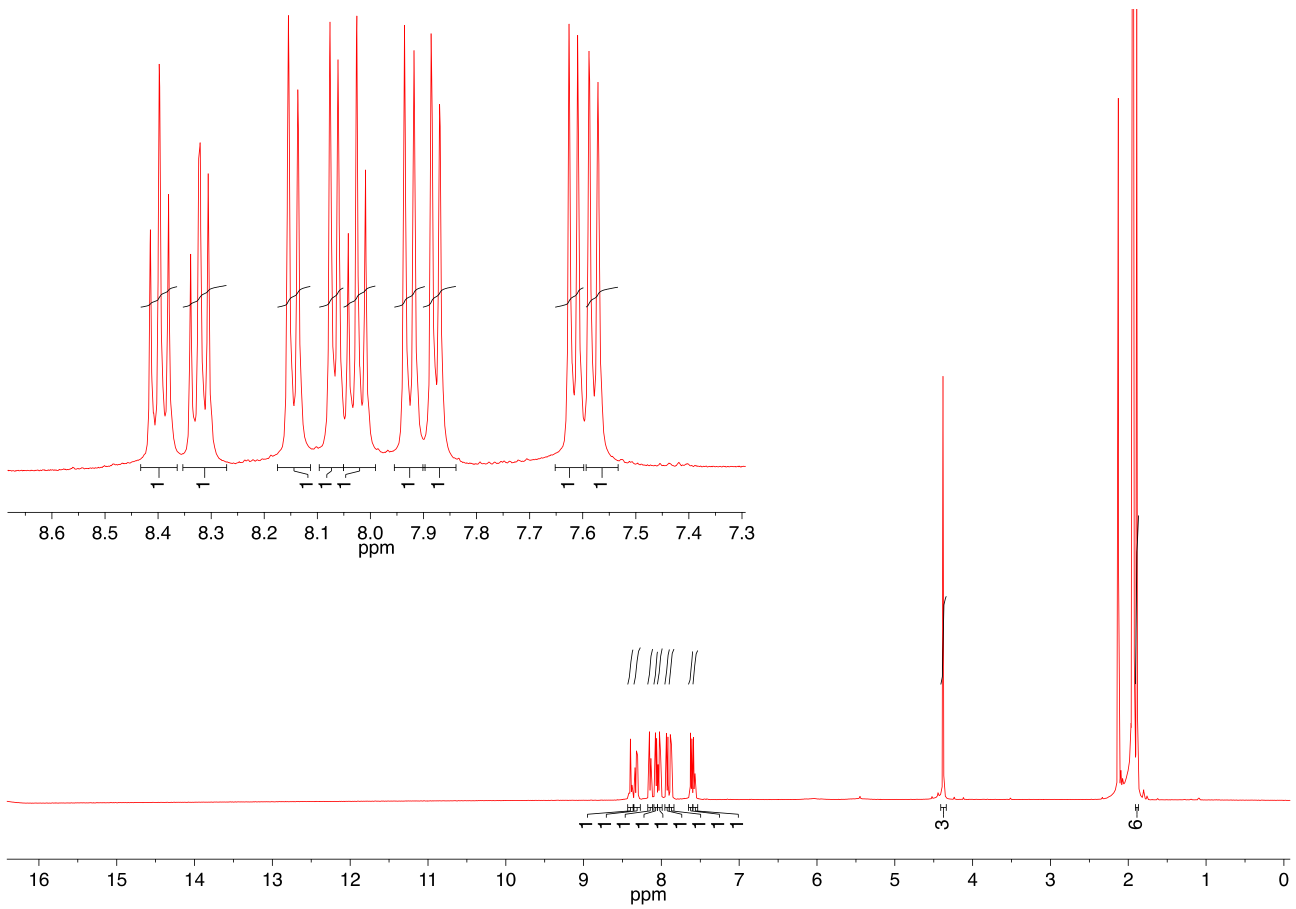

Figure S12. ^{1}H NMR spectrum of **7** recorded in CD_3CN (500 MHz).

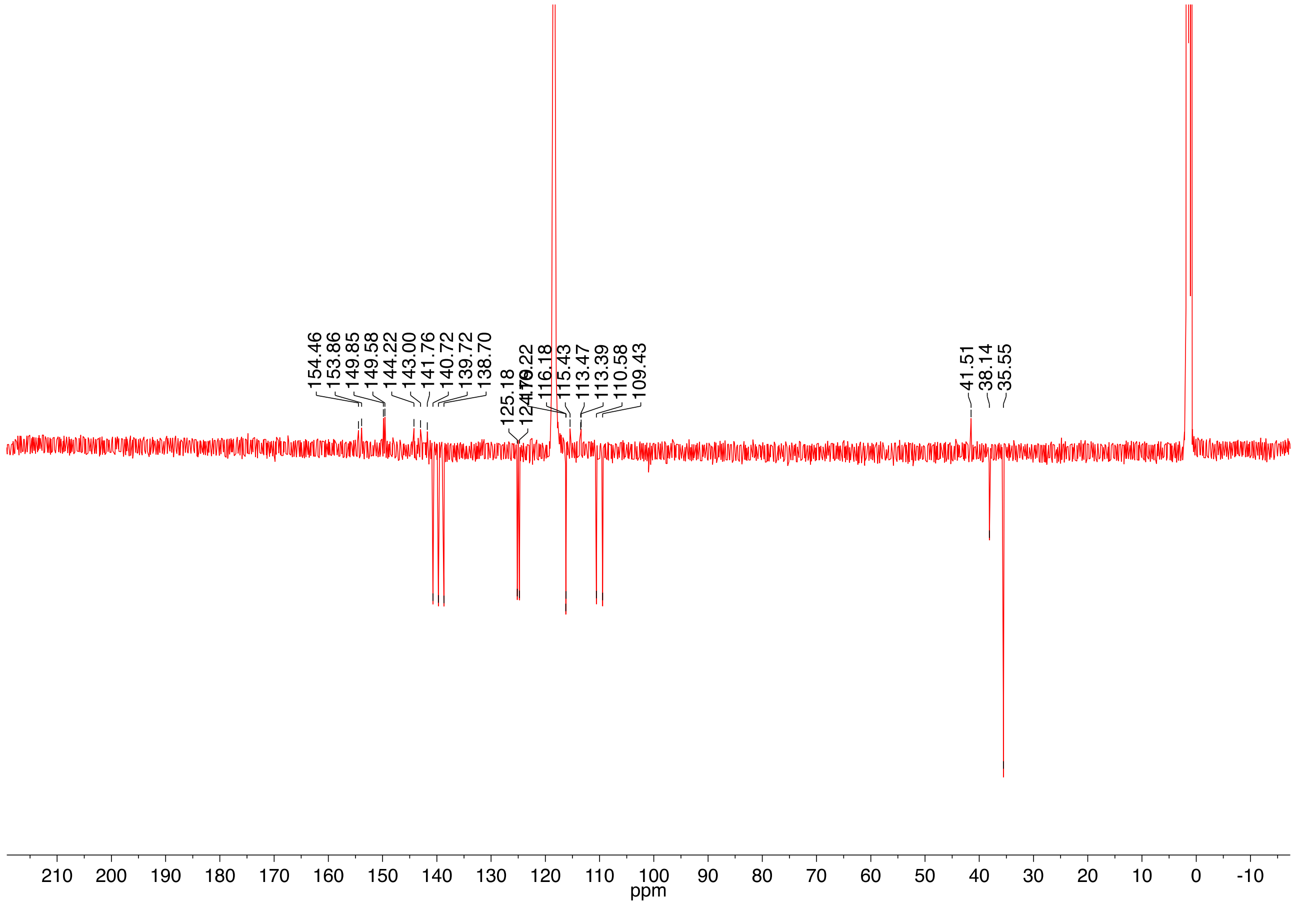

Figure S13. ^{13}C APT NMR spectrum of **7** recorded in CD_3CN (126 MHz).

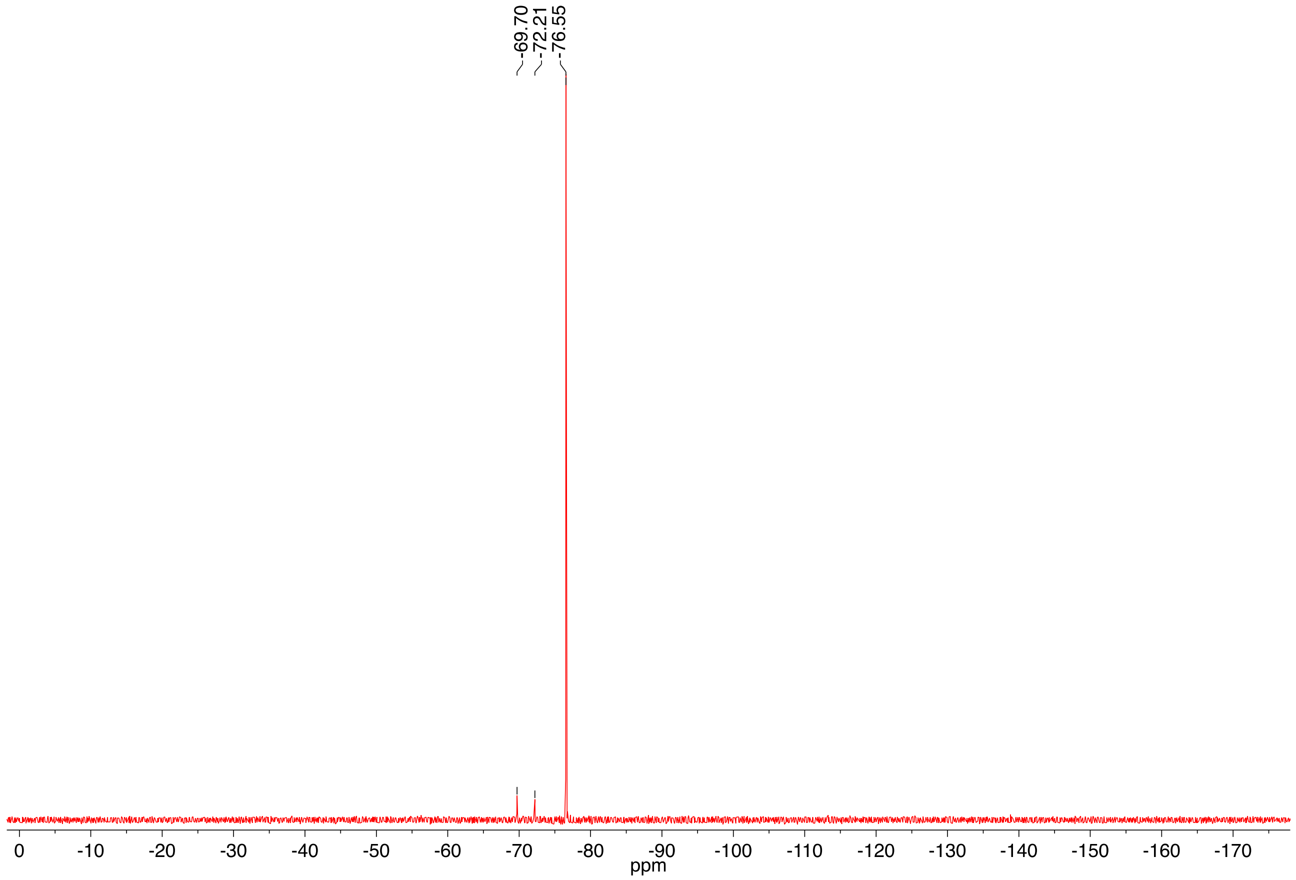

Figure S14. ^{19}F NMR spectrum of **7** recorded in DMSO-d_6 (282 MHz, CF_3COOH at –76.55 ppm as reference).

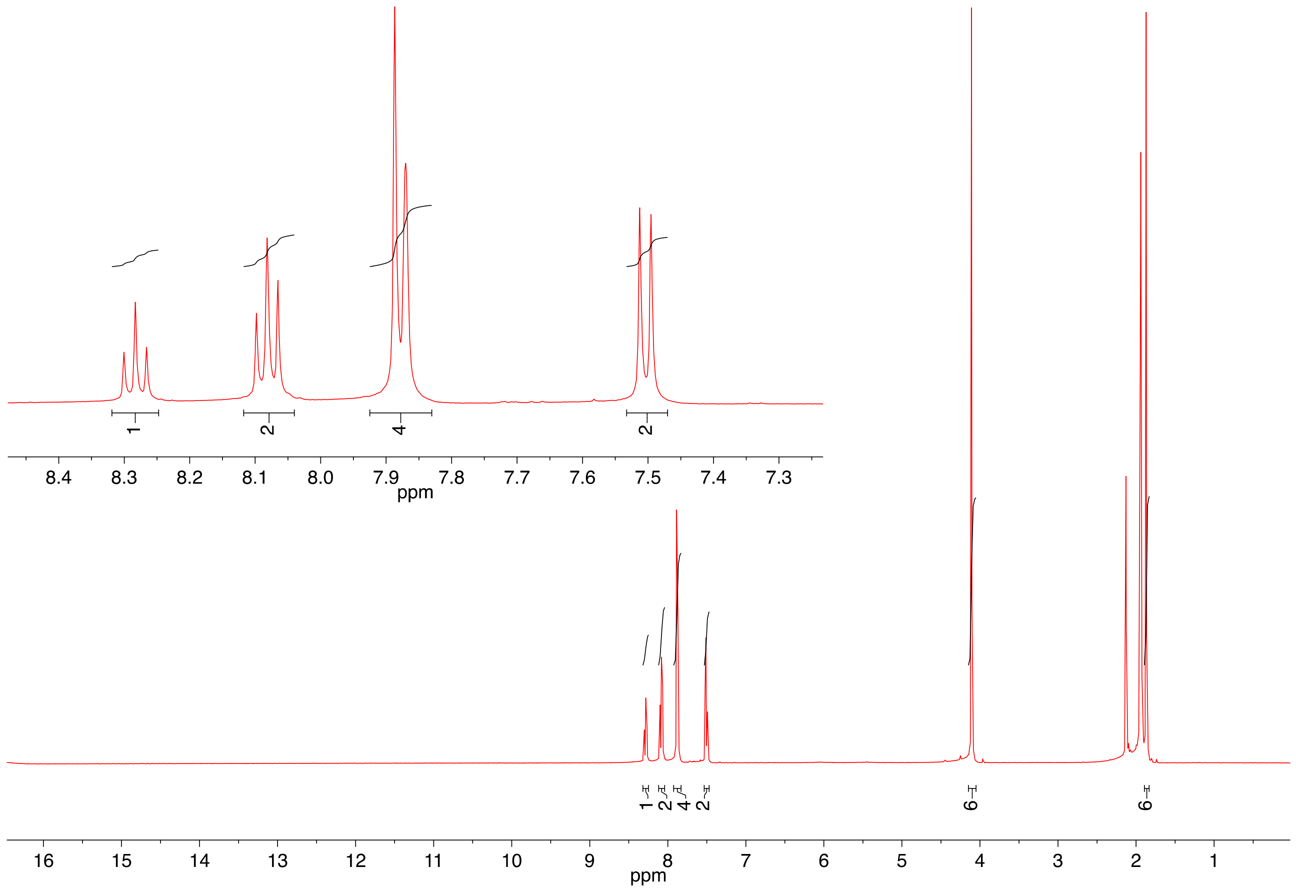

Figure S15. ^{1}H NMR spectrum of **8** recorded in CD_3CN (500 MHz).

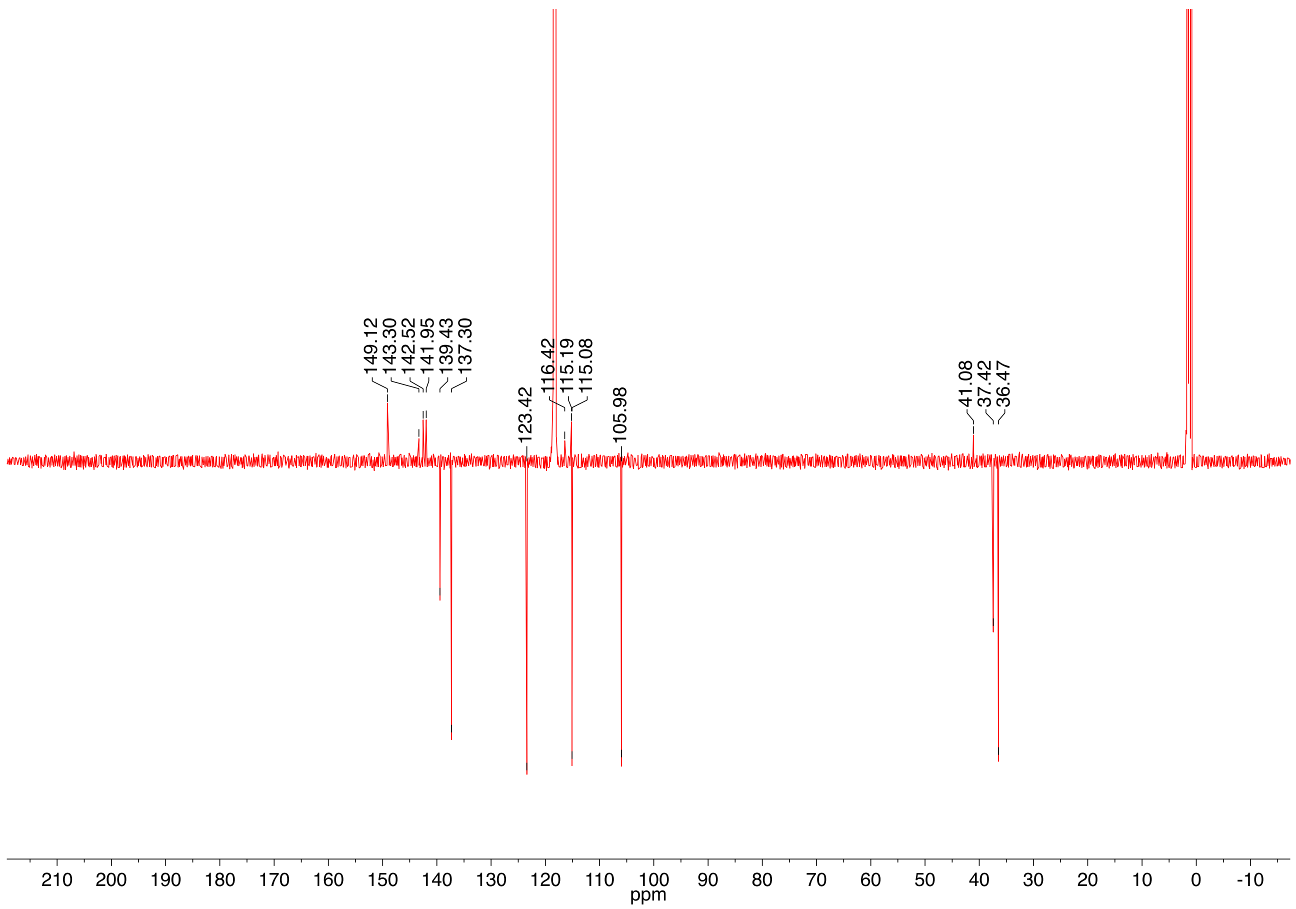

Figure S16. ^{13}C APT NMR spectrum of **8** recorded in CD_3CN (126 MHz).

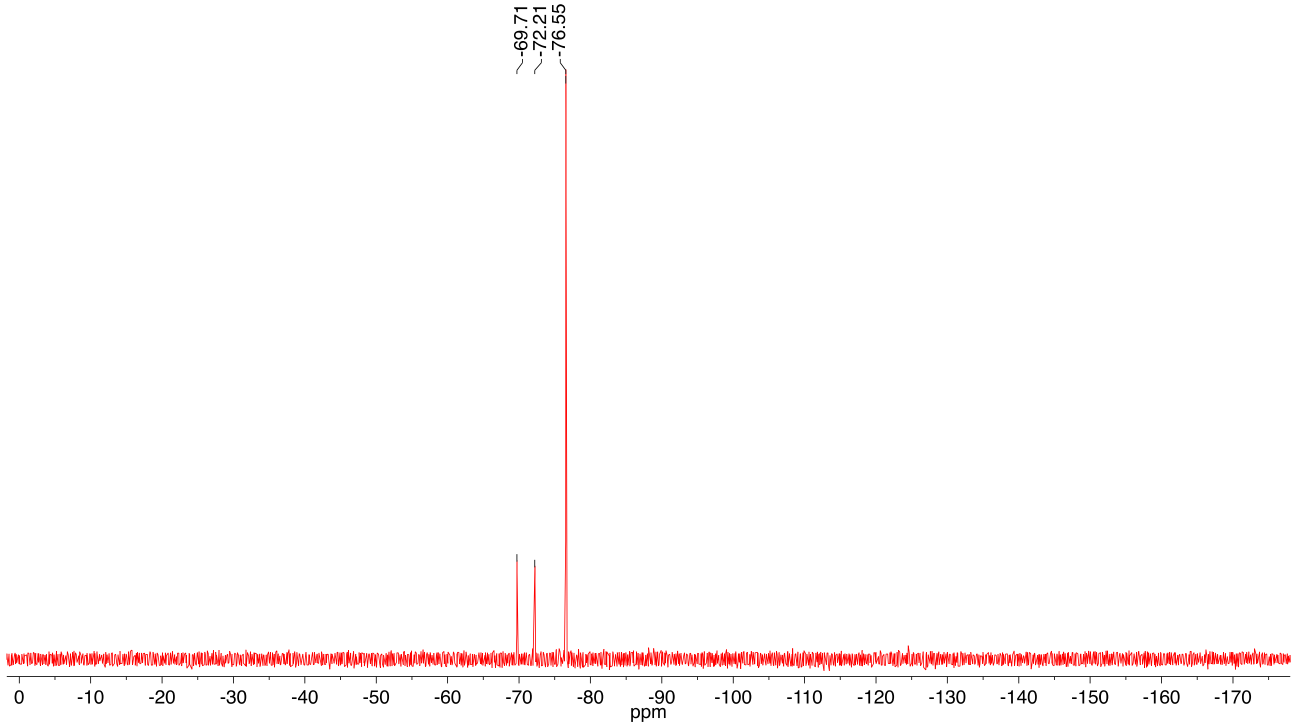

Figure S17. ^{19}F NMR spectrum of **8** recorded in DMSO-d_6 (282 MHz, CF_3COOH at –76.55 ppm as reference).

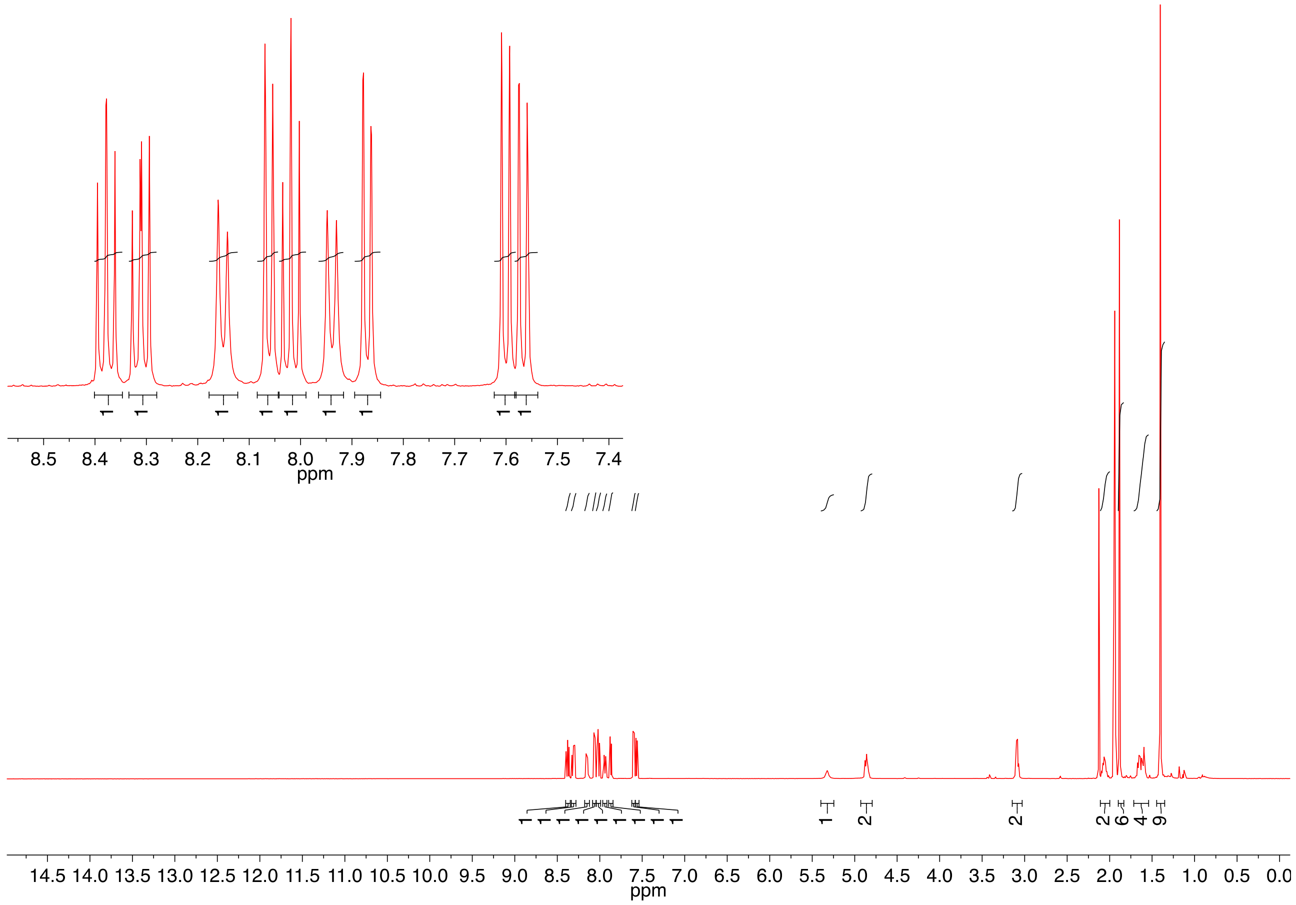

Figure S18. ^{1}H NMR spectrum of **9** recorded in CD_3CN (500 MHz).

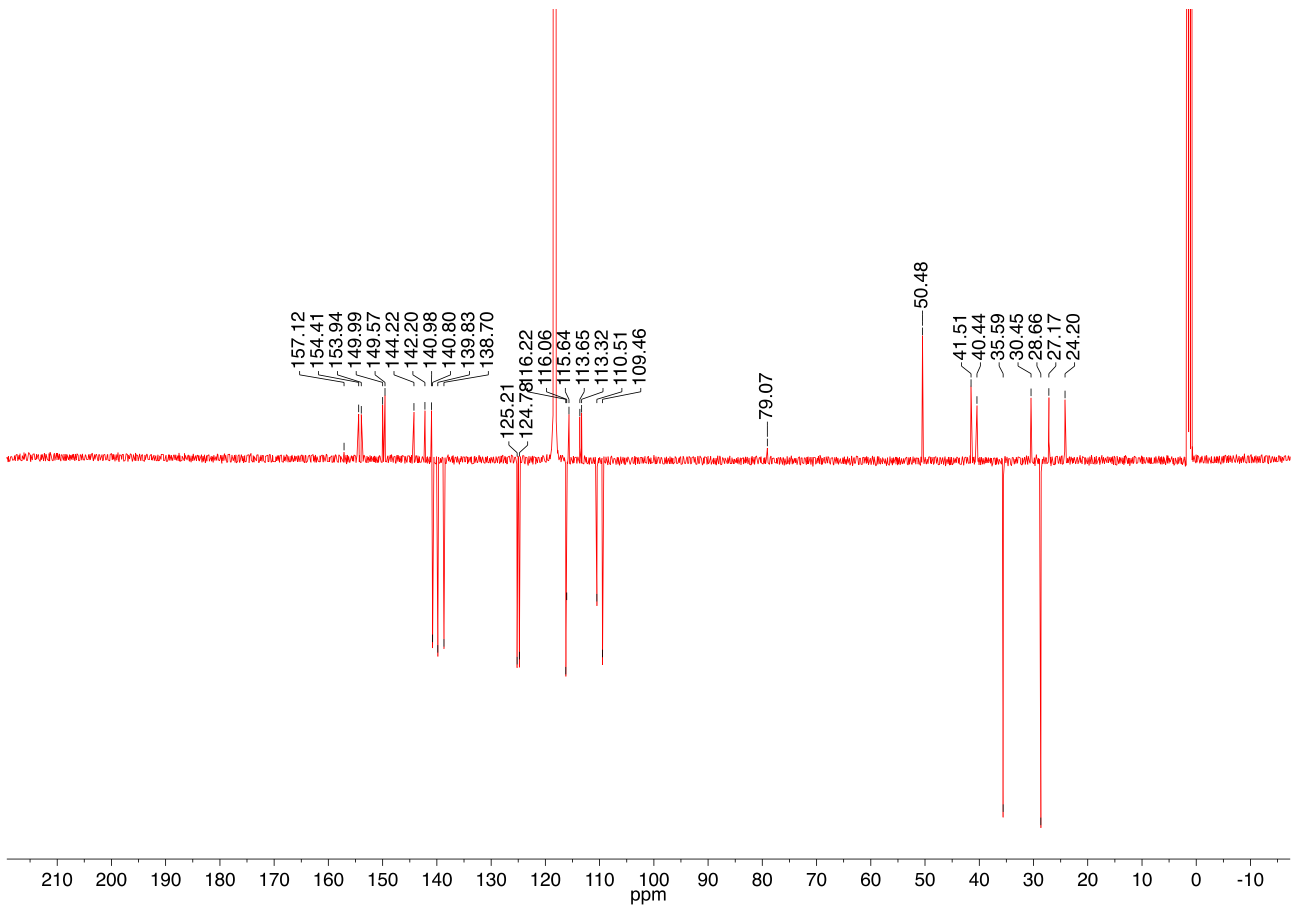

Figure S19. ^{13}C APT NMR spectrum of **9** recorded in CD_3CN (126 MHz).

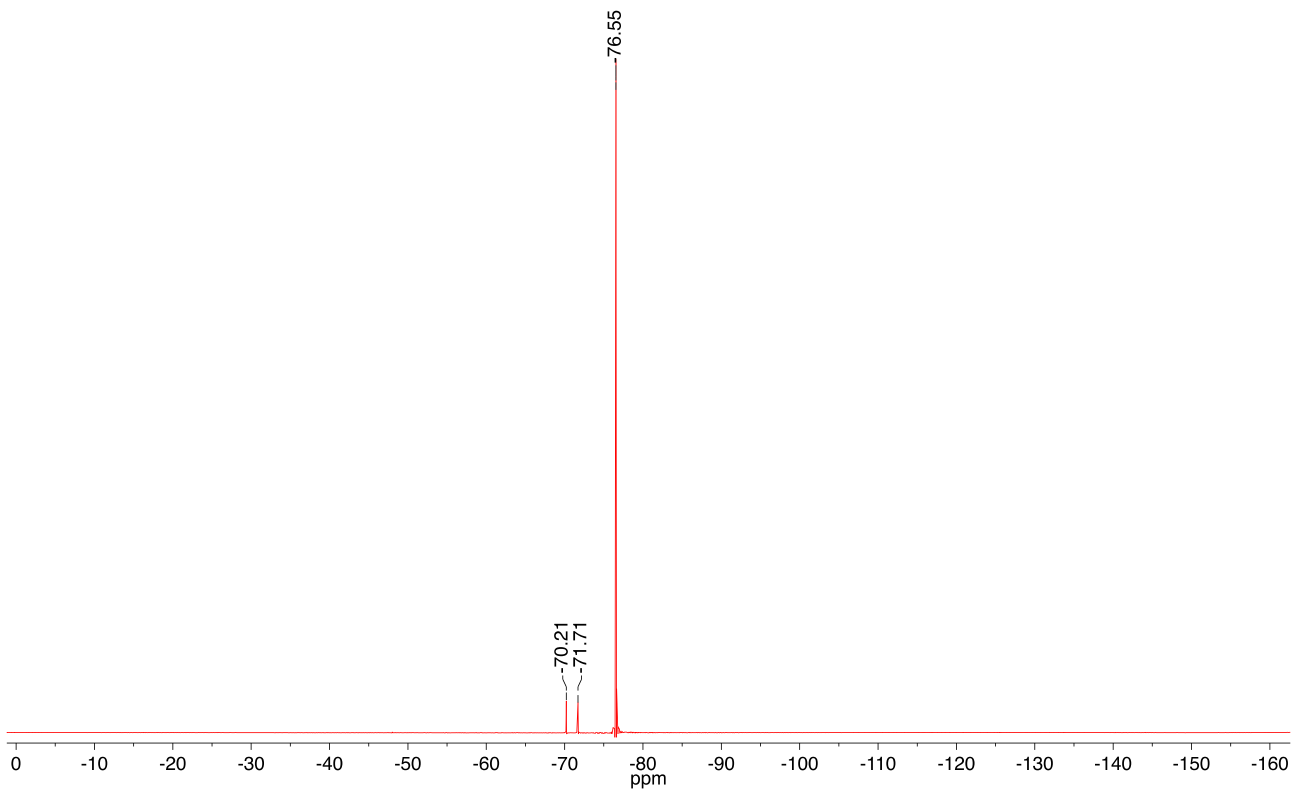

Figure S20. ^{19}F NMR spectrum of **9** recorded in CD_3CN (480 MHz, CF_3COOH at –76.55 ppm as reference).

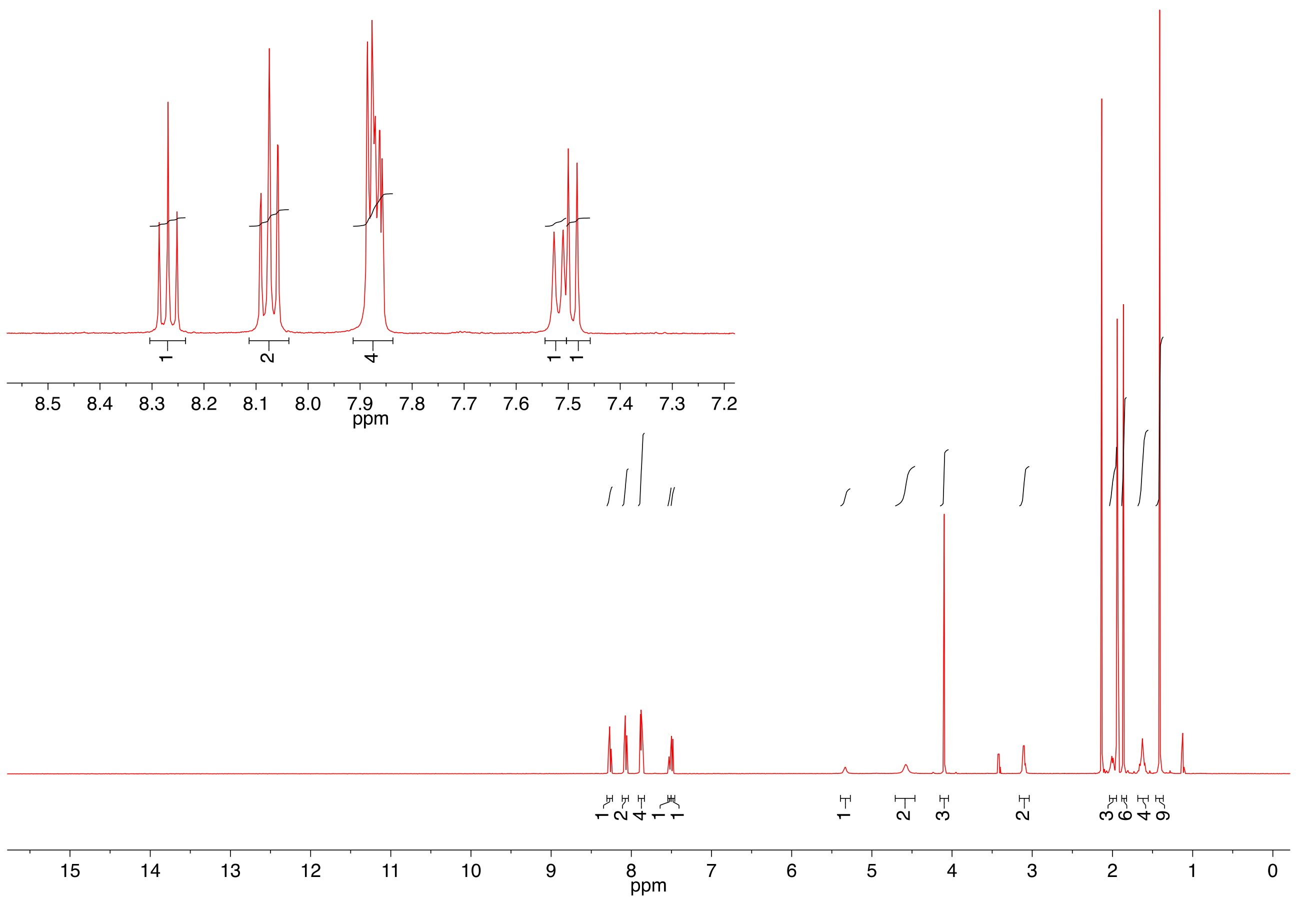

Figure S21. ^{1}H NMR spectrum of **10** recorded in CD_3CN. (500 MHz, the signals at 3.42 ppm and 1.12 ppm arise from residual diethyl ether).

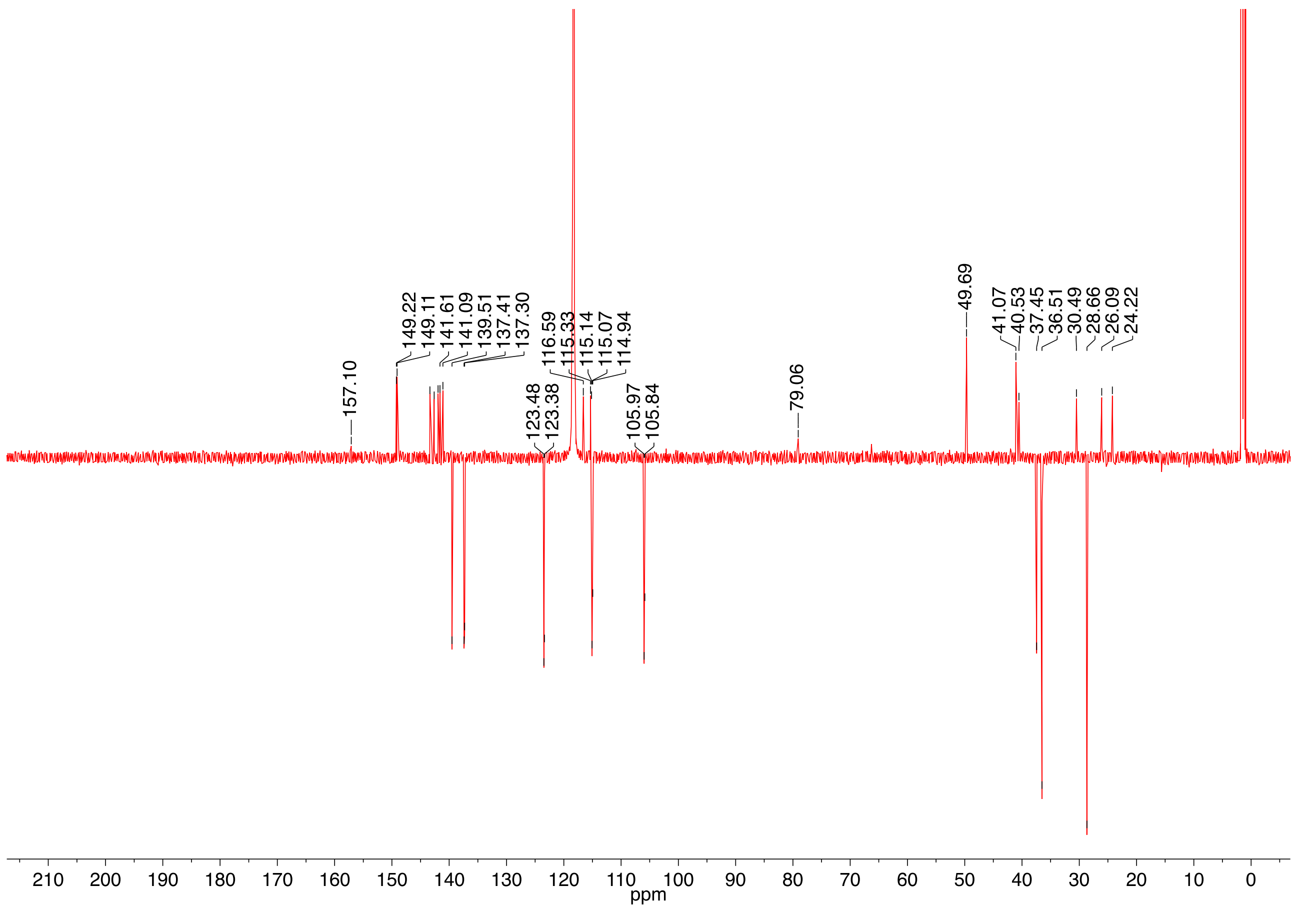

Figure S22. ^{13}C APT NMR spectrum of **10** in CD_3CN. (126 MHz, the signals at 15.63(–) ppm and 66.32(+) ppm arise from residual diethyl ether).

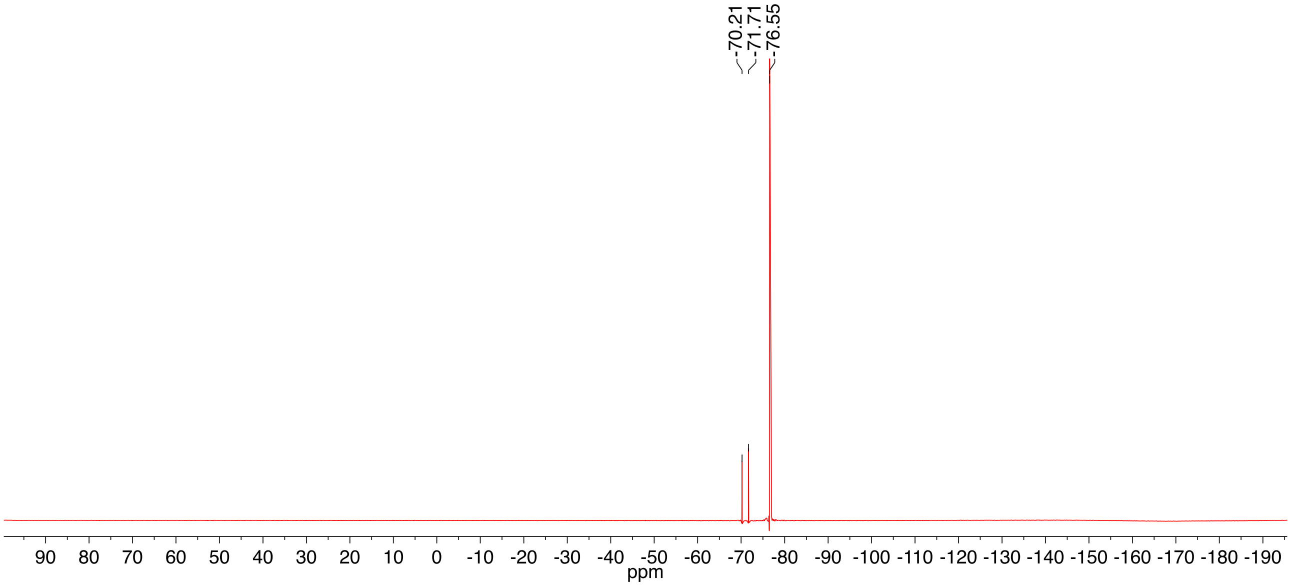

Figure S23. ^{19}F NMR spectrum of **10** in CD_3CN (480 MHz, CF_3COOH at –76.55 ppm as reference).

ANISOTROPY SPECTRA

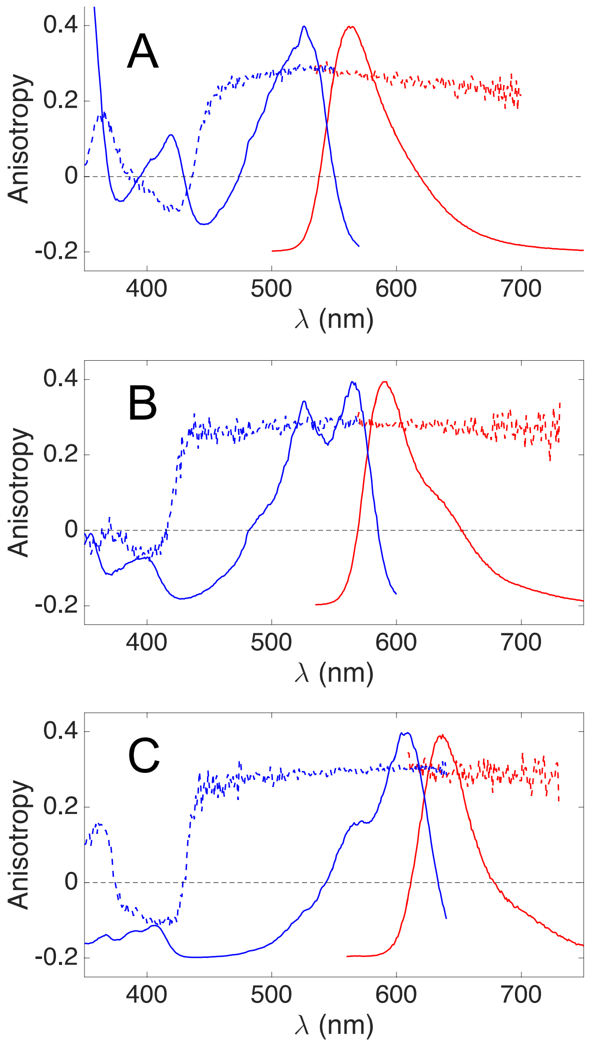

Figure S24. Steady-state fluorescence and excitation anisotropy spectra for **6** (A), **7** (B), and **8** (C) measured in glycerol at 0 °C. The fluorescence anisotropies were measured with excitation of **6**, **7**, and **8** at 490 nm, 525 nm, and 550 nm, respectively. The excitation anisotropies were measured at the fluorescence signal at 580 nm, 610 nm, and 650 nm for **6**, **7**, and **8**, respectively. It is noticed that the maximum anisotropy does not reach the theoretical limits of 0.4 (or -0.2), which is a consequence of partial loss of anisotropy due to molecular rotation in the still liquid glycerol at 0 °C.

SPECTROSCOPIC DATA FOR 8 AND CALCEIN RED ORANGE

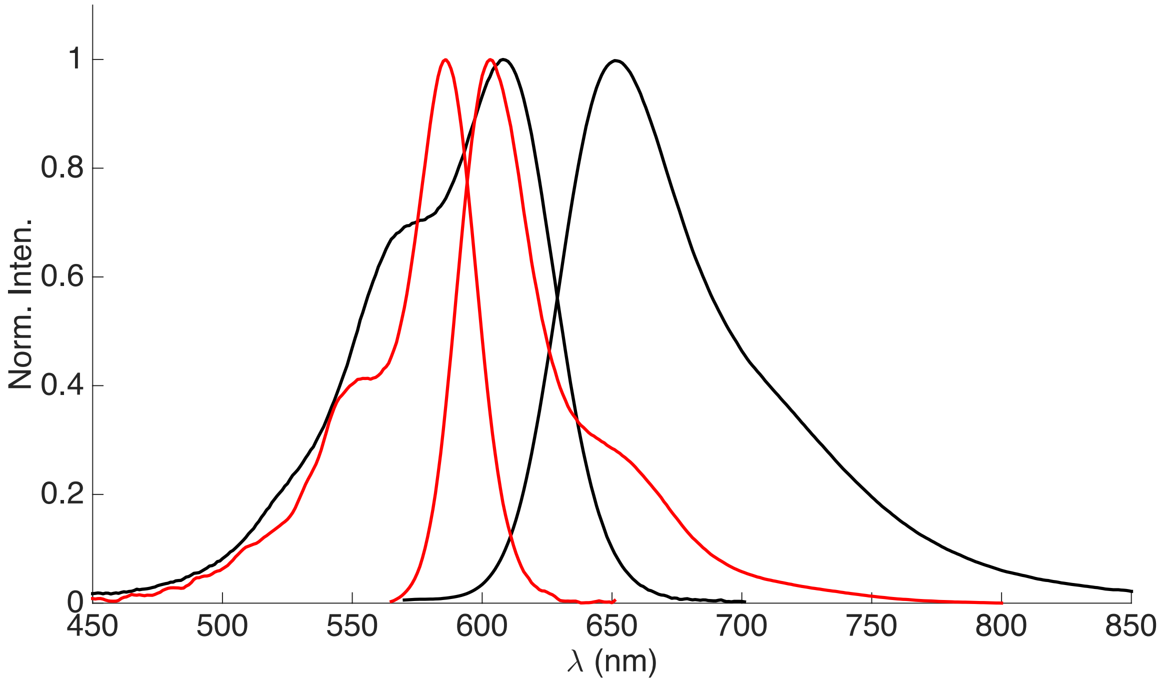

Figure S25. Normalized absorption and fluorescence spectra of **8** (black) and Calcein Red-Orange (red) measured in DMSO solution.

Table S1. Optical properties of 8 measured in: DMSO, 1 vol% DMSO:H_2O, and 1 vol% DMSO:PBS solutions and Calcein Red-Orange measured in DMSO solution.

	8 (DMSO)	8 (1 vol% DMSO:H_2O)	8 (1 vol% DMSO:PBS)	Calcein Red-Orange (DMSO)
λ_{abs} (nm)[a]	609	599	599	586
λ_f (nm)[b]	651	638	638	603
Φ_f[c]	0.17	0.10	0.09	-
τ_f (ns)[d]	4.9	3.4 (92 %)<br>7.0 (8 %)	3.4 (83 %)<br>7.1 (17 %)	3.7
τ_r (ns)[e]	29.1	34.0	37.8	-

[a]Wavelength at the maximum intensity of the $S_0 \rightarrow S_1$ transition. [b]Wavelength at the maximum intensity of the fluorescence spectrum. [c]Fluorescence quantum yield. The quantum yield of oxazine 170 in absolute ethanol solution (Φ = 0.58) was used as reference.[2] [d]Fluorescence lifetime. The numbers in parentheses are the fractional contribution of the fluorescence lifetime component: $\tau_i\alpha_i / \Sigma \tau_j\alpha_j$. [e]Radiative lifetime calculated from the major contributing fluorescence lifetime component and the quantum yields.

TIME-GATED IMAGING

Table S2. Time-resolved fluorescence data measured for CDATA (8) and Calcein Red-Orange in HEK293 cells.

Compound	τ_1 (ns)[b]	f_1 (%)[c]	τ_2 (ns)[b]	f_2 (%)[c]	τ_3 (ns)[b]	f_3 (%)[c]	χ^2 [d]
**8**	**1.4**	**8**	**4.6**	**33**	**11.6**	**59**	**0.99**
**Calcein Red-Orange**	**1.8**	**22**	**4.2**	**78**	**-**	**-**	**1.08**
**8 + Calcein Red-Orange**	**1.4**	**23**	**4.1**	**50**	**12.1**	**27**	**1.05**
**Autofluorescence**[a]	**0.5**	**66**	**4.8**	**34**	**-**	**-**	**1.18**

[a]Measured in non-stained cells. [b]Fluorescence lifetime determined from tail-fitting procedure. [c]Fractional contribution f_i of the fluorescence lifetime component: $f_i = \tau_i\alpha_i/\Sigma\,\tau_j\alpha_j$. [d]$\chi^2$ fitting value.

CONFOCAL MICROSCOPY

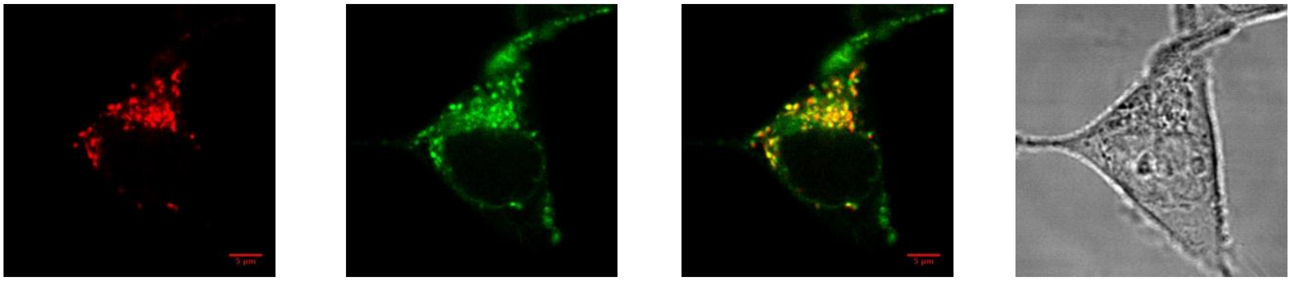

A B C D

Figure S26. Confocal microscope picture of HEK293 cells stained with **7** and co-stained with MitoTracker Green. A: Fluorescence signal from **7**. B: Fluorescence signal from MitoTracker Green. C: Overlay of pictures A and B. D: Transmission image.

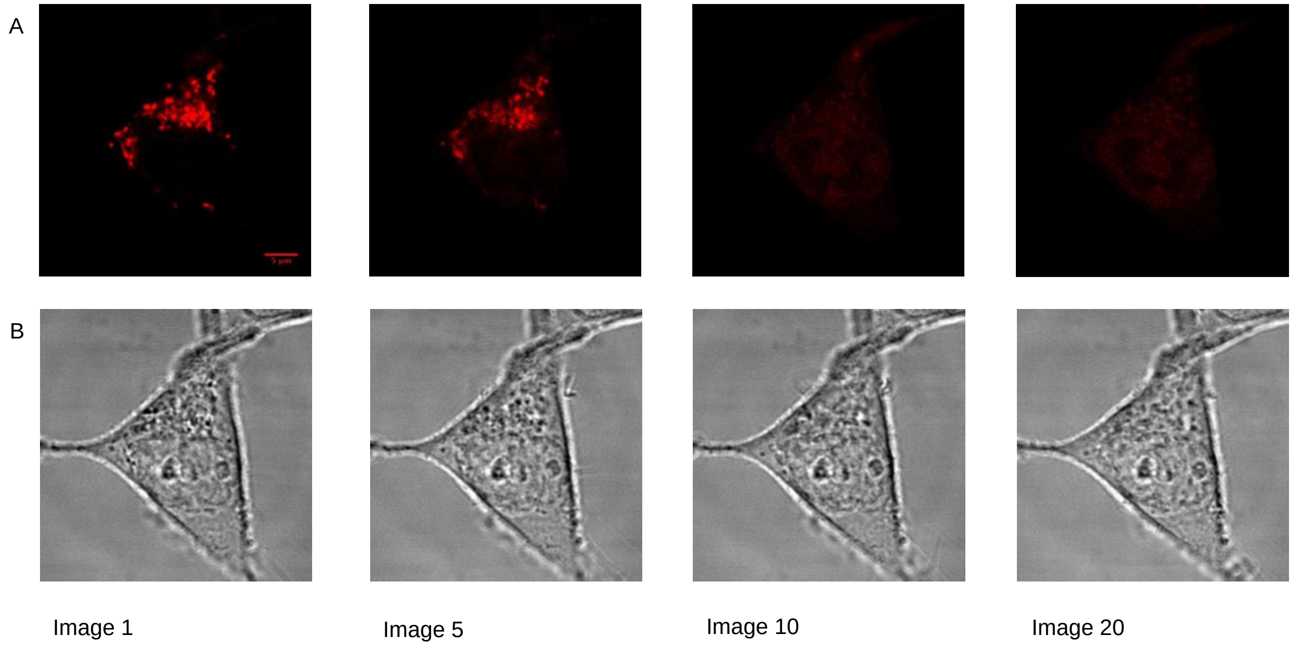

Figure S27. A: Photobleaching of **7** in HEK293 cells upon recording of images in a confocal microscope. The transmission images are shown in B.

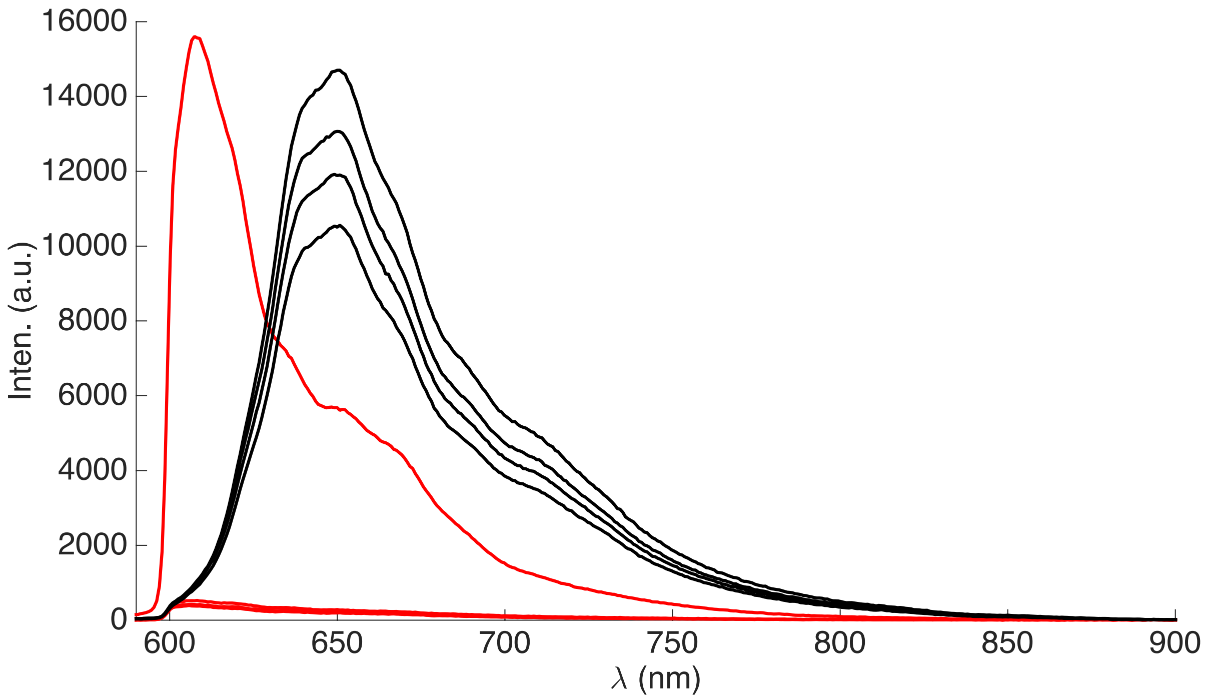

Figure S28. Fluorescence spectra associated with the photobleaching of **8** and Calcein Red-Orange in HEK293 cells upon irradiation with a 100 W Hg lamp. The spectra were measured upon continuous irradiation (545 nm) of **8** (black) and Calcein Red-Orange (red) after 5 sec, 1 min, 2 min, 5 min, and 10 min of irradiation time.

COMPUTATIONAL RESULTS

In order to further investigate how **6**, **7**, and **8** relate to the DAOTA chromophore, vertical excitation energy calculations were performed at the TD-PCM-CAM-B3LYP/6-311+G(2d,p)//PCM-B3LYP/6-31+G(d) level with the PCM simulating CH_2Cl_2. The calculations were performed on the cationic part of **6**, **7**, and **8**, *i.e.* $\mathbf{6}^+$, $\mathbf{7}^+$, and $\mathbf{8}^+$, and compared to calculated results to the cationic part of DAOTA *i.e.* $DAOTA^+$. The calculations yield excitation energies (Tables S3-S6), which on average are 4700 cm^{-1} higher than the experimental values (Table 1). A similar offset has been found in results of TDDFT calculations performed on similar compounds,[7] and the calculated energies are in agreement with the experimental energies when this offset is taken into account.

4,8-DIMETHYL-4,8-DIAZA-12-OXATRIANGULENIUM ($DAOTA^+$)

Nuclear coordinates for optimized structure:

N 1.1782940000 0.0731360000 2.4354600000

N 1.1782940000 0.0731360000 -2.4354600000

C 1.2016600000 0.0073010000 0.0000000000

C -0.1978810000 0.0340210000 0.0000000000

C -0.9093080000 0.0235830000 -1.2200190000

C 1.9021320000 -0.0181770000 -1.2498660000

C -0.2111870000 0.0246360000 -2.4597080000

C -0.2111870000 0.0246360000 2.4597080000

C -2.3226110000 -0.0065820000 1.1933010000

C 1.9021320000 -0.0181770000 1.2498660000

C -0.9692050000 -0.0282520000 3.6448640000

C -0.9093080000 0.0235830000 1.2200190000

C -3.0624550000 -0.0384920000 -2.3625320000

O -2.9933610000 -0.0123360000 0.0000000000

C 3.2988640000 -0.1347540000 -1.2281440000

C -3.0624550000 -0.0384920000 2.3625320000

C 3.2988640000 -0.1347540000 1.2281440000

C -2.3612480000 -0.0549790000 -3.5754700000

C -0.9692050000 -0.0282520000 -3.6448640000

C 1.9177700000 0.2067600000 3.6992610000

C -2.3612480000 -0.0549790000 3.5754700000

C -2.3226110000 -0.0065820000 -1.1933010000

C 1.9177700000 0.2067600000 -3.6992610000

H 2.8036450000 0.8188380000 -3.5350800000

H 1.2996490000 0.7219980000 -4.4304620000

H 2.2124800000 -0.7725180000 -4.0909500000

C 3.9573260000 -0.1984310000 0.0000000000

H -4.1458270000 -0.0630240000 -2.3279320000

H -0.4988210000 -0.0729290000 -4.6173320000

H 3.8793700000 -0.2101020000 2.1369980000

H -0.4988210000 -0.0729290000 4.6173320000

H 2.8036450000 0.8188380000 3.5350800000

H 1.2996490000 0.7219980000 4.4304620000

H 2.2124800000 -0.7725180000 4.0909500000

H -4.1458270000 -0.0630240000 2.3279320000

H 3.8793700000 -0.2101020000 -2.1369980000

H -2.9236610000 -0.0997340000 4.5032720000

H 5.0374280000 -0.3125180000 0.0000000000

H -2.9236610000 -0.0997340000 -4.5032720000

Point group: C_s

E(PCM-B3LYP/6-31+G(d)) = –993.921099204 Hartree

E_{ZPV}(PCM-B3LYP/6-31+G(d)) = 0.312331 Hartree

E(PCM-CAM-B3LYP/6-311+G(2d,p)//PCM-B3LYP/6-31+G(d)) = –993.624634506 Hartree

Table S3. Excited state calculations (TD-PCM-CAM-B3LYP/6-311+G(2d,p)//PCM-B3LYP/6-31+G(d)) for DAOTA$^+$.

Transition[a]	ΔE (eV)[b]	λ (nm)[c]	f[d]	Character[e]
S_0 (A') → S_1 (A'')	2.85	436	0.2454	HOMO(π) → LUMO(π*), 98 %
S_0 (A') → S_2 (A')	3.28	378	0.1078	HOMO–1(π) → LUMO(π*), 98 %
S_0 (A') → S_3 (A'')	4.01	309	0.0147	HOMO(π) → LUMO+1(π*), 81 %
S_0 (A') → S_4 (A')	4.19	296	0.0070	HOMO(π) → LUMO+2(π*), 39 %<br>HOMO–2(π) → LUMO(π*), 38 %<br>HOMO–1(π) → LUMO+1(π*), 17 %

[a]Spatial symmetries of the states, S_n (n = 0-4) are given in parentheses posterior the states. [b]Electronic excitation energy. [c]Electronic excitation wavelength. [d]Oscillator strength. [e]The Kohn-Sham orbitals mainly involved in the transitions are listed together with their contribution to the excitation. Contributions less than 10 % are not listed.

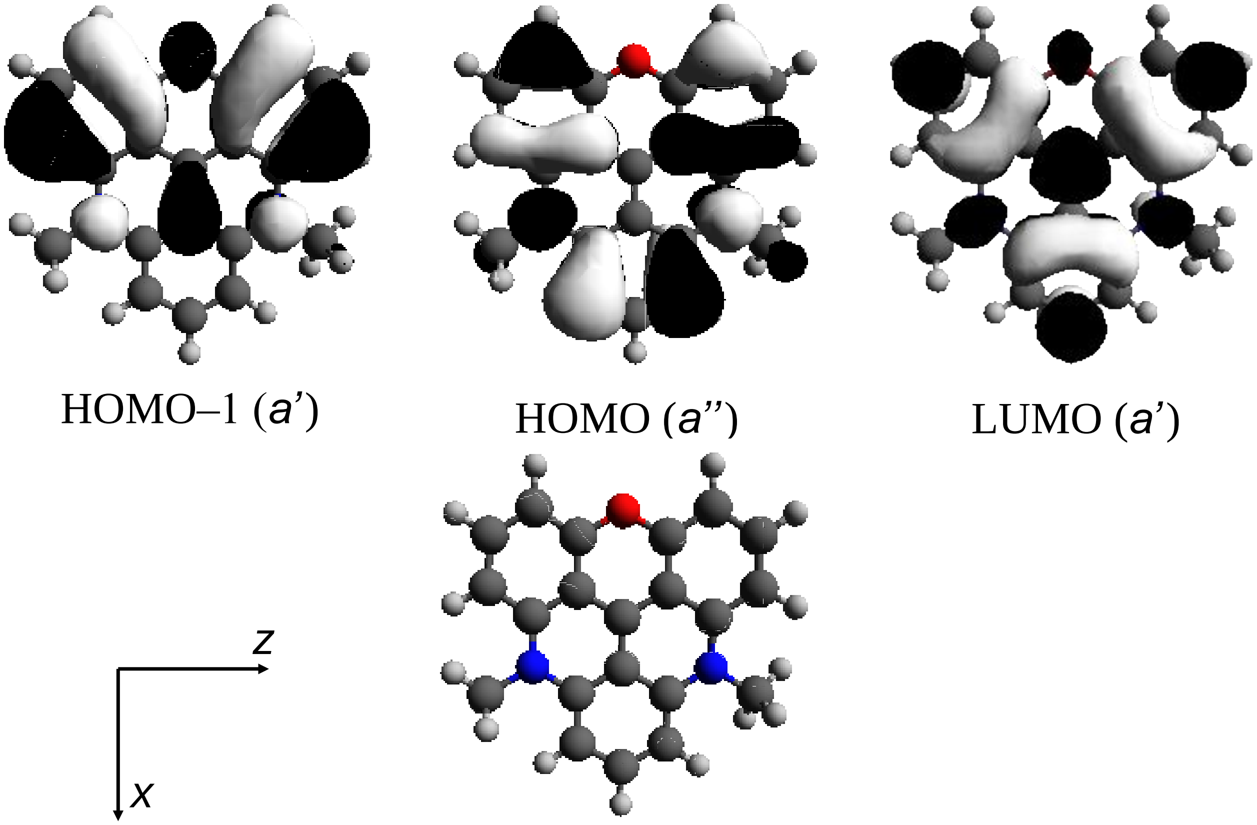

Figure S29. Calculated orbitals (PCM-TD-CAM-B3LYP/6-311+G(2d,p)//PCM-B3LYP/6-31+G(d)) for DAOTA$^+$: HOMO–1, HOMO, and LUMO. Spatial symmetries of the orbitals are given in parentheses. Grey atoms: carbon; white atoms: hydrogen; blue atoms: nitrogen; red atom: oxygen.

12,12-DIMETHYLBENZO[8,1]ISOCHROMENO[3,4,5,6-*KLMN*]XANTHENIUM (CDOTA$^+$) (6$^+$)

Nuclear coordinates for optimized structure:

C 0.0000000000 3.6778260000 -0.1818780000

C 0.0000000000 2.4299480000 0.4315650000

C 0.0000000000 1.2381400000 -0.3355090000

C 0.0000000000 1.2852610000 -1.7539690000

C 0.0000000000 2.5431310000 -2.3496020000

C 0.0000000000 3.7156040000 -1.5732990000

C 0.0000000000 0.0000000000 0.3542780000

C 0.0000000000 0.0000000000 -2.5946990000

C 0.0000000000 -1.2852610000 -1.7539690000

C 0.0000000000 -1.2381400000 -0.3355090000

C 0.0000000000 -2.4299480000 0.4315650000

C 0.0000000000 -3.6778260000 -0.1818780000

C 0.0000000000 -3.7156040000 -1.5732990000

C 0.0000000000 -2.5431310000 -2.3496020000

H 0.0000000000 4.5809780000 0.4184190000

H 0.0000000000 2.6430610000 -3.4296510000

H 0.0000000000 4.6787930000 -2.0747890000

H 0.0000000000 -4.5809780000 0.4184190000

H 0.0000000000 -4.6787930000 -2.0747890000

H 0.0000000000 -2.6430610000 -3.4296510000

C 0.0000000000 0.0000000000 1.7594550000

C 0.0000000000 -1.2213160000 2.4762100000

C 0.0000000000 1.2213160000 2.4762100000

C 0.0000000000 1.2302510000 3.8635570000

C 0.0000000000 -1.2302510000 3.8635570000

H 0.0000000000 2.1697200000 4.4046740000

H 0.0000000000 -2.1697200000 4.4046740000

C 0.0000000000 0.0000000000 4.5340790000

H 0.0000000000 0.0000000000 5.6196560000

C -1.2701190000 0.0000000000 -3.4948250000

H -1.2837940000 -0.8835010000 -4.1391960000

H -2.1813250000 0.0000000000 -2.8884650000

H -1.2837940000 0.8835010000 -4.1391960000

C 1.2701190000 0.0000000000 -3.4948250000

H 2.1813250000 0.0000000000 -2.8884650000

H 1.2837940000 -0.8835010000 -4.1391960000

H 1.2837940000 0.8835010000 -4.1391960000

O 0.0000000000 2.4005880000 1.7981930000

O 0.0000000000 -2.4005880000 1.7981930000

Point group: C_{2v}

E(PCM-B3LYP/6-31+G(d)) = –997.720705364 Hartree

E_{ZPV}(PCM-B3LYP/6-31+G(d)) = 0.309502 Hartree

E(PCM-CAM-B3LYP/6-311+G(2d,p)//PCM-B3LYP/6-31+G(d)) = –997.427297560 Hartree

Table S4. Excited state calculations (TD-PCM-CAM-B3LYP/6-311+G(2d,p)//PCM-B3LYP/6-31+G(d)) for CDOTA$^+$ (6^+).

**Transition**[a]	***ΔE* (eV)**[b]	***λ* (nm)**[c]	***f***[d]	**Character**[e]
**S_0 (A_1) → S_1 (B_2)**	**2.91 eV**	**427**	**0.1805**	**HOMO(π) → LUMO(π*), 98 %**
**S_0 (A_1) → S_2 (A_1)**	**3.48 eV**	**357**	**0.1439**	**HOMO–1(π) → LUMO(π*), 98 %**
**S_0 (A_1) → S_3 (B_2)**	**3.91 eV**	**317**	**0.2112**	**HOMO–2(π) → LUMO(π*), 94 %**
**S_0 (A_1) → S_4 (A_1)**	**4.06 eV**	**305**	**0.2950**	**HOMO–3(π) → LUMO(π*), 91 %**

[a]Spatial symmetries of the states, S_n (n = 0-4) are given in parentheses posterior the states. [b]Electronic excitation energy. [c]Electronic excitation wavelength. [d]Oscillator strength. [e]The Kohn-Sham orbitals mainly involved in the transitions are listed together with their contribution to the excitation. Contributions less than 10 % are not listed.

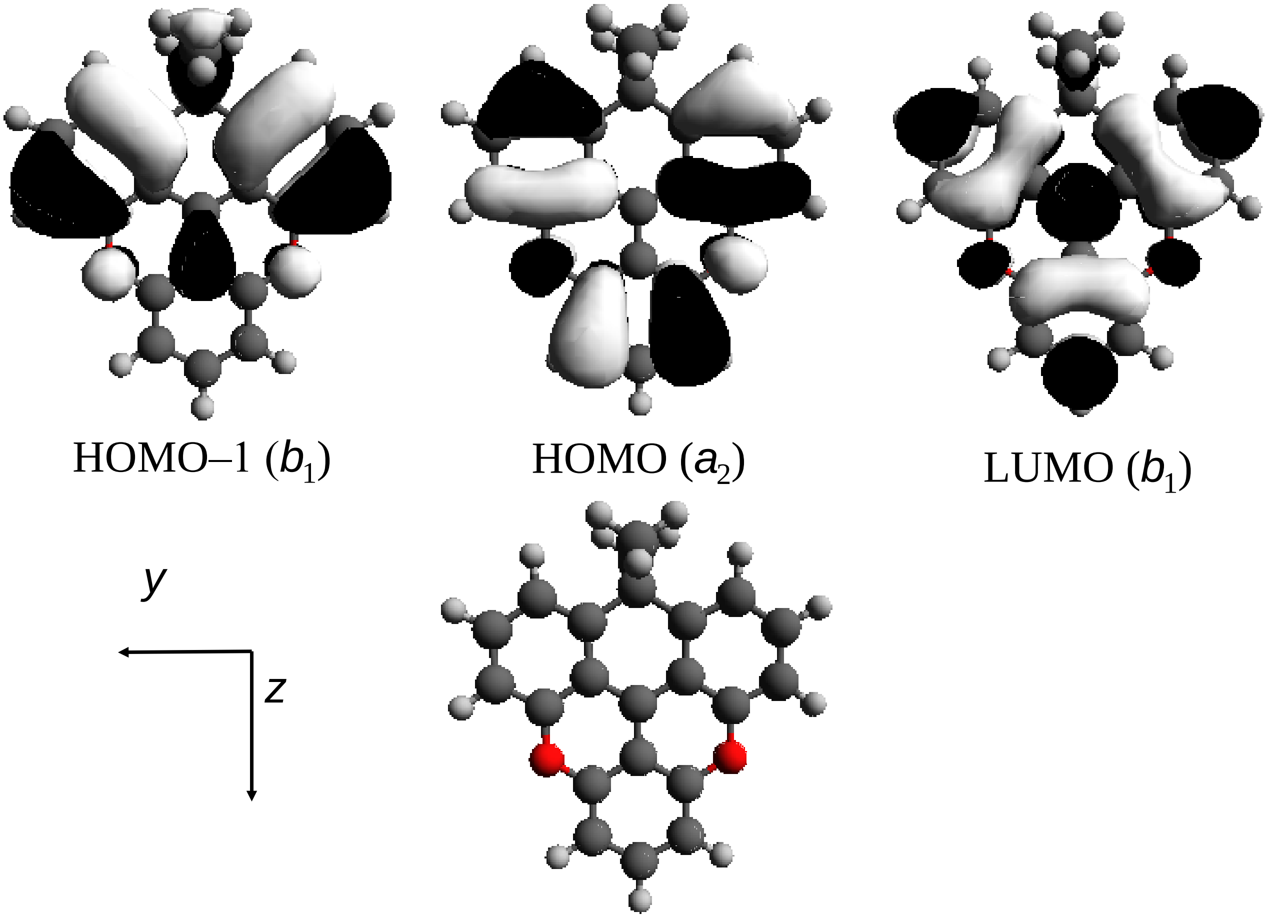

Figure S30 Calculated orbitals (PCM-TD-CAM-B3LYP/6-311+G(2d,p)//PCM-B3LYP/6-31+G(d)) for CDOTA$^+$ (**6**$^+$): HOMO–1, HOMO, and LUMO. Spatial symmetries of the orbitals are given in parentheses. Grey atoms: carbon; white atoms: hydrogen; red atoms: oxygen.

8,12,12-TRIMETHYL-8,12-DIHYDRO-3*A*^{2}*H*-BENZO[8,1]ISOCHROMENO[3,4,5,6-*KLMN*]ACRIDINIUM (CAOTA$^+$) (7$^+$)

Nuclear coordinates for optimized structure:

C 1.7738790000 -3.4338430000 0.0394840000

C 0.7298440000 -2.5151880000 0.0192310000

C 0.9662050000 -1.1227810000 0.0203810000

C 2.2929950000 -0.6319760000 0.0259760000

C 3.3322870000 -1.5614430000 0.0479190000

C 3.0748960000 -2.9404260000 0.0570680000

C -0.1613120000 -0.2388050000 0.0140830000

C 2.5810080000 0.8709990000 -0.0020980000

C 1.3121630000 1.7359780000 -0.0292320000

C 0.0045170000 1.1665800000 -0.0000300000

C -1.1647220000 1.9962580000 0.0031110000

C -1.0015040000 3.3943860000 -0.0769110000

C 0.2741160000 3.9289130000 -0.1174180000

C 1.4204760000 3.1172670000 -0.0796740000

H 1.5601050000 -4.4972620000 0.0394500000

H 4.3646110000 -1.2289650000 0.0553060000

H 3.9080560000 -3.6367850000 0.0739730000

H -1.8512560000 4.0599950000 -0.1344740000

H 0.3898910000 5.0066870000 -0.1850760000

H 2.3929420000 3.5960790000 -0.1024620000

C -1.4554090000 -0.7873190000 -0.0007840000

C -2.6120980000 0.0548880000 0.0027860000

C -1.6241100000 -2.1999740000 -0.0277340000

C -2.8768870000 -2.7809700000 -0.0778590000

C -3.8830690000 -0.5464190000 -0.0727480000

H -2.9782760000 -3.8601130000 -0.1020580000

H -4.7857030000 0.0456070000 -0.1282650000

C -3.9921410000 -1.9331590000 -0.1113140000

H -4.9826350000 -2.3734140000 -0.1750670000

C 3.3990790000 1.2430840000 1.2692910000

H 3.6615110000 2.3049740000 1.2632700000

H 2.8239440000 1.0351410000 2.1775990000

H 4.3289860000 0.6686730000 1.3119360000

C 3.4224410000 1.1834020000 -1.2741540000

H 2.8641430000 0.9314580000 -2.1817610000

H 3.6855300000 2.2440950000 -1.3152990000

H 4.3532950000 0.6091850000 -1.2725660000

O -0.5391470000 -3.0211540000 -0.0078270000

N -2.4262880000 1.4231490000 0.0803830000

C -3.6028310000 2.2946560000 0.2473140000

H -3.3325630000 3.1560800000 0.8544510000

H -3.9857560000 2.6276600000 -0.7225650000

H -4.3785810000 1.7534510000 0.7834000000

Point group: C_1

E(PCM-B3LYP/6-31+G(d)) = –1017.18309944 Hartree

E_{ZPV}(PCM-B3LYP/6-31+G(d)) = 0.351528 Hartree

E(PCM-CAM-B3LYP/6-311+G(2d,p)//PCM-B3LYP/6-31+G(d)) = –1016.86513498 Hartree

Table S5. Excited state calculations (TD-PCM-CAM-B3LYP/6-311+G(2d,p)//PCM-B3LYP/6-31+G(d)) for CAOTA$^+$ (7$^+$).

Transition	ΔE (eV)^a	λ (nm)^b	f^c	Character^d
$S_0 \rightarrow S_1$	2.76	449	0.2488	HOMO(π) → LUMO(π*), 98 %
$S_0 \rightarrow S_2$	3.61	343	0.1168	HOMO–1(π) → LUMO(π*), 96 %
$S_0 \rightarrow S_3$	4.00	310	0.0514	HOMO–2(π) → LUMO(π*), 52 %<br>HOMO(π) → LUMO+1(π*), 30 %
$S_0 \rightarrow S_4$	4.16	298	0.1335	HOMO–3(π) → LUMO(π*), 62 %<br>HOMO–2(π) → LUMO(π*), 24 %

^{a}Electronic excitation energy. ^{b}Electronic excitation wavelength. ^{c}Oscillator strength. ^{d}The Kohn-Sham orbitals mainly involved in the transitions are listed together with their contribution to the excitation. Contributions less than 10 % are not listed.

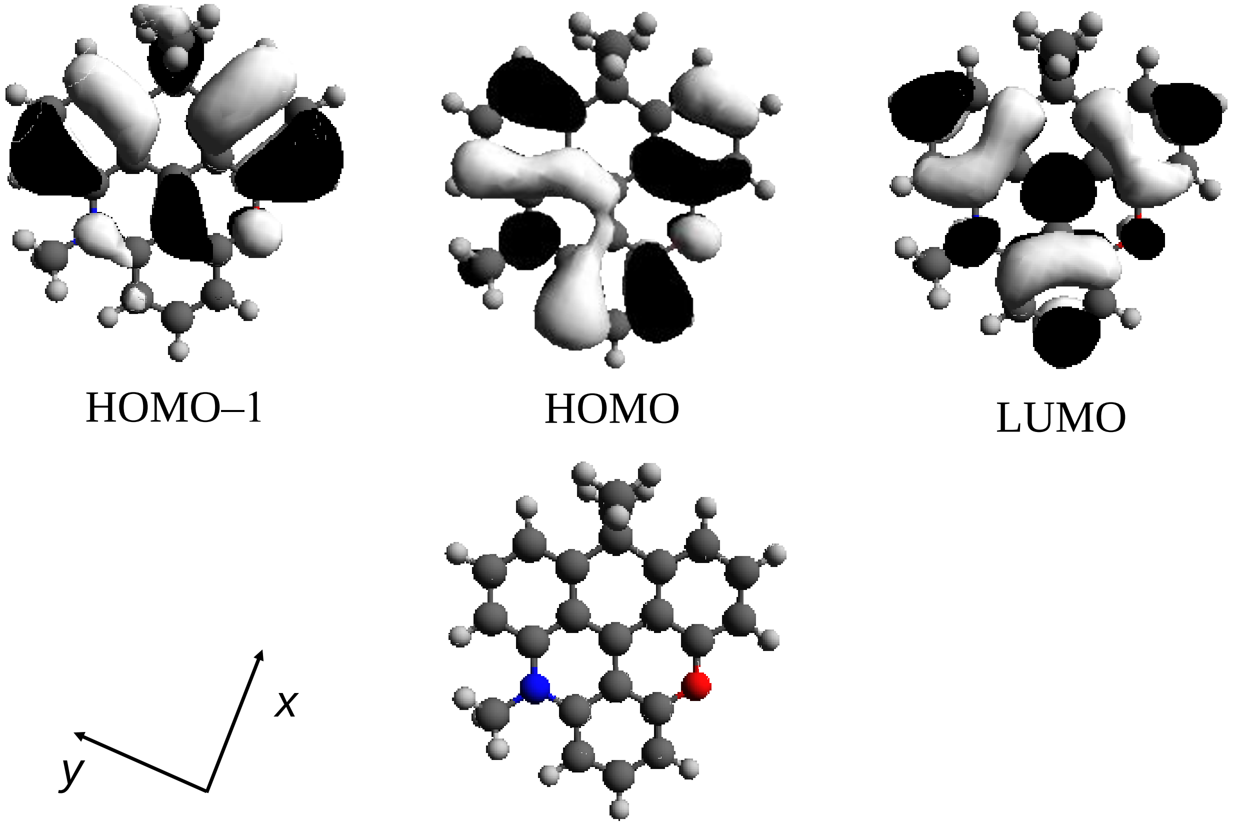

Figure S31 Calculated orbitals (PCM-TD-CAM-B3LYP/6-311+G(2d,p)//PCM-B3LYP/6-31+G(d)) for $CAOTA^+$ ($\mathbf{7}^+$): HOMO–1, HOMO, and LUMO. Grey atoms: carbon; white atoms: hydrogen; red atom: oxygen; blue atom: nitrogen.

4,8,12,12-TETRAMETHYL-8,12-DIHYDROBENZO[8,1]ISOQUINOLINO[3,4,5,6-*KLMN*]ACRIDINIUM ($\mathbf{CDATA^+}$) ($\mathbf{8^+}$)

Nuclear coordinates for optimized structure:

C 0.0000000000 3.6918650000 -0.4981610000

C 0.0845660000 2.4614930000 0.1832710000

C 0.0243070000 1.2450990000 -0.5639420000

C -0.0262480000 1.2786500000 -1.9840710000

C -0.0781570000 2.5131760000 -2.6202740000

C -0.0862740000 3.7028620000 -1.8799310000

C 0.0000000000 0.0000000000 0.1397330000

C 0.0000000000 0.0000000000 -2.8237250000

C 0.0262480000 -1.2786500000 -1.9840710000

C -0.0243070000 -1.2450990000 -0.5639420000

C -0.0845660000 -2.4614930000 0.1832710000

C 0.0000000000 -3.6918650000 -0.4981610000

C 0.0862740000 -3.7028620000 -1.8799310000

C 0.0781570000 -2.5131760000 -2.6202740000

H -0.0288580000 4.6303510000 0.0380130000

H -0.1212360000 2.5745900000 -3.7019540000

H -0.1610730000 4.6547750000 -2.3976540000

H 0.0288580000 -4.6303510000 0.0380130000

H 0.1610730000 -4.6547750000 -2.3976540000

H 0.1212360000 -2.5745900000 -3.7019540000

C 0.0000000000 0.0000000000 1.5467000000

C -0.0988550000 -1.2374370000 2.2718710000

C 0.0988550000 1.2374370000 2.2718710000

C 0.0705820000 1.2194760000 3.6734810000

C -0.0705820000 -1.2194760000 3.6734810000

H 0.0876430000 2.1322650000 4.2516490000

H -0.0876430000 -2.1322650000 4.2516490000

C 0.0000000000 0.0000000000 4.3412540000

H 0.0000000000 0.0000000000 5.4273990000

C -1.2706730000 -0.0396550000 -3.7230370000

H -1.2617710000 -0.9242490000 -4.3662570000

H -2.1793140000 -0.0677640000 -3.1125620000

H -1.3177380000 0.8414470000 -4.3691480000

C 1.2706730000 0.0396550000 -3.7230370000

H 2.1793140000 0.0677640000 -3.1125620000

H 1.3177380000 -0.8414470000 -4.3691480000

H 1.2617710000 0.9242490000 -4.3662570000

N 0.2287680000 2.4178460000 1.5628650000

N -0.2287680000 -2.4178460000 1.5628650000

C 0.5357840000 3.6549980000 2.2978480000

H -0.3762630000 4.1479500000 2.6502860000

H 1.1752770000 3.4180200000 3.1466880000

H 1.0923850000 4.3312450000 1.6529760000

C -0.5357840000 -3.6549980000 2.2978480000

H -1.0923850000 -4.3312450000 1.6529760000

H 0.3762630000 -4.1479500000 2.6502860000

H -1.1752770000 -3.4180200000 3.1466880000

Point group: C_2

E(PCM-B3LYP/6-31+G(d)) = –1036.64055365 Hartree

E_{ZPV}(PCM-B3LYP/6-31+G(d)) = 0.392762 Hartree

E(PCM-CAM-B3LYP/6-311+G(2d,p)// PCM-B3LYP/6-31+G(d)) = –1036.29775833 Hartree

Table S6. Excited state calculations (TD-PCM-CAM-B3LYP/6-311+G(2d,p)//PCM-B3LYP/6-31+G(d)) for CDATA$^+$ (8$^+$).

Transition	ΔE (eV)	λ (nm)	f	Character
S_0 (A) → S_1 (B)	2.62	473	0.2806	HOMO(π) → LUMO(π*), 98 %
S_0 (A) → S_2 (A)	3.60	344	0.1187	HOMO–1(π) → LUMO(π*), 96 %
S_0 (A) → S_3 (B)	3.95	314	0.0040	HOMO(π) → LUMO+1(π*), 74 %<br>HOMO–3(π) → LUMO(π*), 13 %
S_0 (A) → S_4 (A)	4.13	300	0.0136	HOMO–2(π) → LUMO(π*), 61 %<br>HOMO(π) → LUMO+2(π*), 29 %

[a]Spatial symmetries of the states, S_n (n = 0-4) are given in parentheses posterior the states. [b]Electronic excitation energy. [c]Electronic excitation wavelength. [d]Oscillator strength. [e]The Kohn-Sham orbitals mainly involved in the transitions are listed together with their contribution to the excitation. Contributions less than 10 % are not listed.

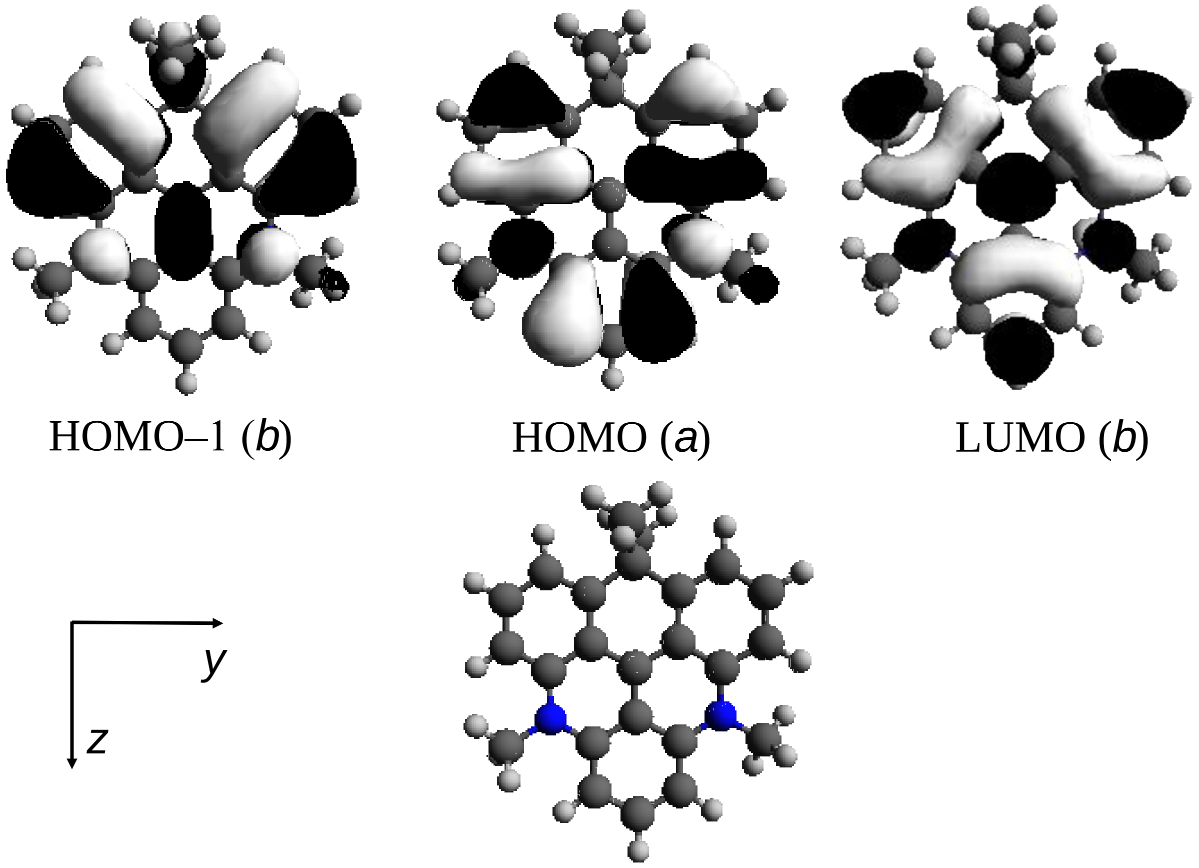

Figure S32. Calculated orbitals (PCM-TD-CAM-B3LYP/6-311+G(2d,p)//PCM-B3LYP/6-31+G(d)) for CDATA$^+$ (**8**$^+$): HOMO–1, HOMO, and LUMO. Spatial symmetries of the orbitals are given in parentheses. Grey atoms: carbon; white atoms: hydrogen; blue atoms: nitrogen.

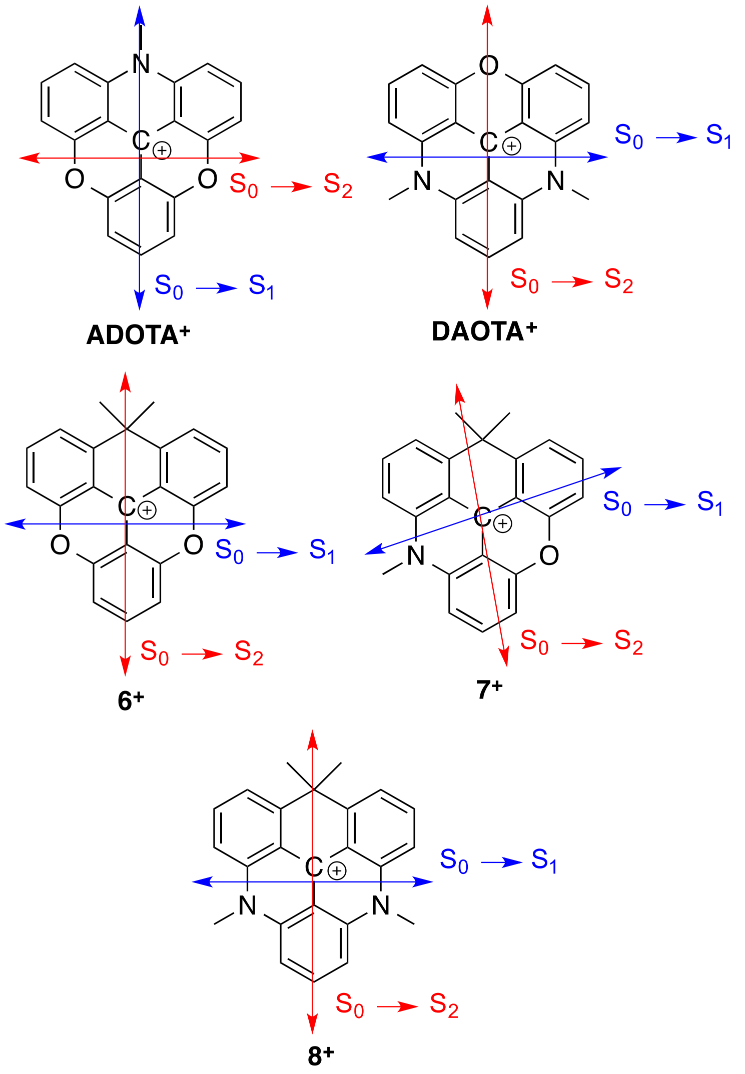

Figure S33. Illustration of the orientations of the transition dipoles for the $S_0 \rightarrow S_1$ (blue) and $S_0 \rightarrow S_2$ (red) transitions for ADOTA$^+$ and DAOTA$^+$. The orientations of CDOTA$^+$ (**6**$^+$**)**, CAOTA$^+$ (**7**$^+$**)**, and CDATA$^+$ (**8**$^+$) were calculated at the TD-PCM-CAMB3LYP/6-311+G(2d,p)//PCM-B3LYP/6-31+G(d) level.

CRYSTAL STRUCTURES

Table S7. Details of crystallographic experiments and models.

Name	**6**	**7**	**8**	DAOTA
Empirical formula	C22 H15 O2, F6 P	C23 H18 N O, F6 P	C24 H21 N2, F6 P	C21 H15 N2 O, F6 P
Formula weight	456.31		482.40	456.32
Crystal system	Triclinic	Triclinic	Orthorhombic	Monoclinic
Space group	P-1	P-1	Pccn	C2/c
a/Å	8.4805(5)	14.162(3)	14.5591(8)	6.861(3)
b/Å	8.5013(4)	15.264(4)	21.6499(15)	10.695(4)
c/Å	14.7698(8)	20.039(5)	13.0273(9)	23.818(10)
α/°	74.017(2)	87.939(5)	90	90
β/°	89.013(2)	85.555(8)	90	96.101(14)
γ/°	64.503(2)	62.365(8)	90	90
Volume/$Å^3$	917.65(9)	3826.0(16)	4106.3(5)	1737.8(12)
Z	2	8	8	4
Rho_calc mg/mm^3	1.651	1.712	1.561	1.744
η /mm-1	0.228	0.298	0.204	0.240
F(000)	464	1931.0	1984	928
Crystal size / mm3	0.012	0.0135	0.00015	0.004
Theta range	2.68-31.37	2.27-23.26	2.30-25.68	3.44-25.03
Reflections collected	23324	22235	29608	16454
Independent reflec-tions	5562	10648	3894	1546
R_{int}	0.0528	0.0817	0.1122	0.1077
Data / restraints / parameters	5562/ 0/340	10648/0/666	3894/0/382	/0/145
Temperature / K	123	123	123	123
R / wR(F^2)	0.0436 / 0.1064	0.2375 / 0.6463	0.0872/0.1015	0.10/0.23
Gof	0.994	2.23	1.041	1.228
Peak/hole/e$Å^{-3}$	0.467/-0.344	1.19/-0.9	0.295/-0.246	0.47/-0.42
Color of crystal	red	violet	violet	red

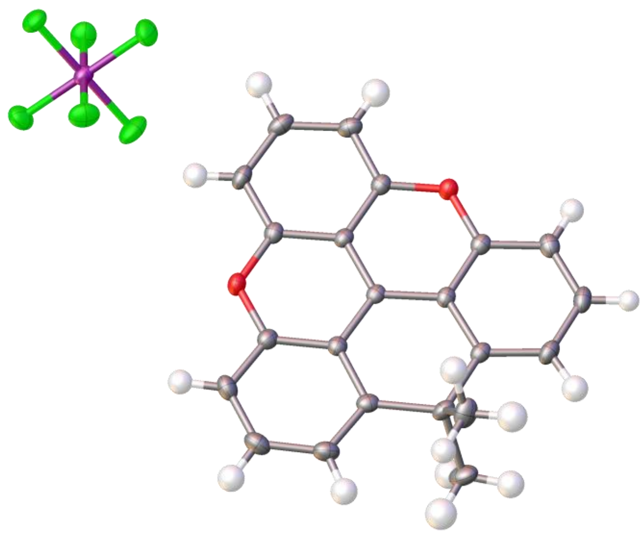

Figure S34. Crystal structure of **6**. The structure is shown with ellipsoids drawn at the 50 % level. Red atoms: oxygen; grey atoms: carbon; white atoms: hydrogen; purple atom: phosphorus; green atoms: fluorine.

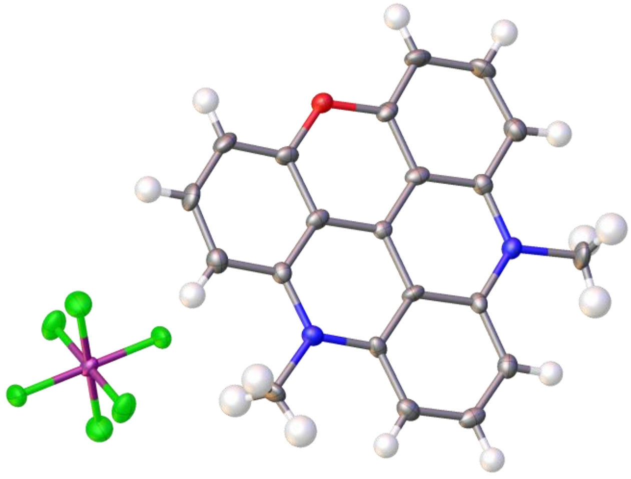

Figure S35 Crystal structure of **DAOTA**. The structure is shown with ellipsoids drawn at the 50 % level. Blue atoms: nitrogen; red atoms: oxygen; grey atoms: carbon; white atoms: hydrogen; purple atom: phosphorus; green atoms: fluorine.

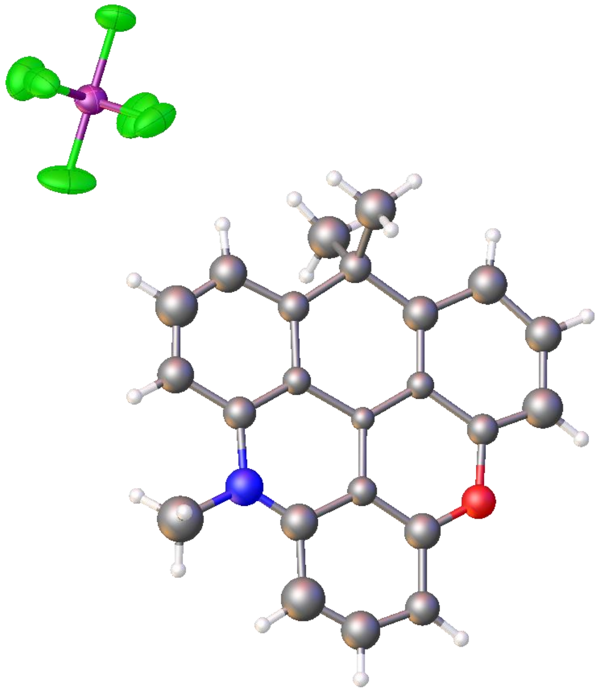

Figure S36 Crystal structure of **7**. The structure is shown with ellipsoids drawn at the 50 % level. Only a single molecule and PF_6 is shown (there are four of each in the asymmetric unit). Blue atoms: nitrogen; red atoms: oxygen; grey atoms: carbon; white atoms: hydrogen; purple atom: phosphorus; green atoms: fluorine.

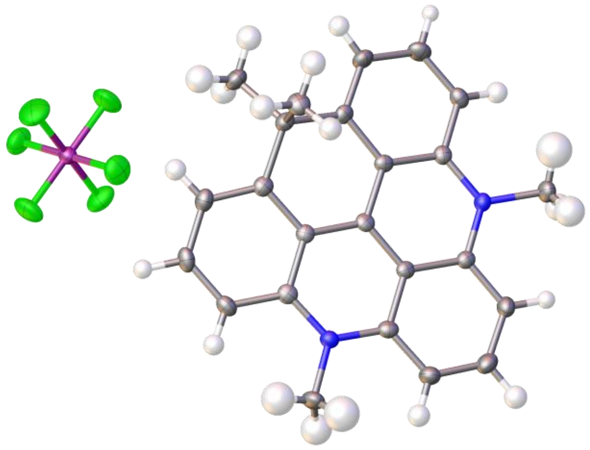

Figure S37. Crystal structure of **8**. The structure is shown with ellipsoids drawn at the 50 % level. Blue atoms: nitrogen; red atoms: oxygen; grey atoms: carbon; white atoms: hydrogen; purple atom: phosphorus; green atoms: fluorine.

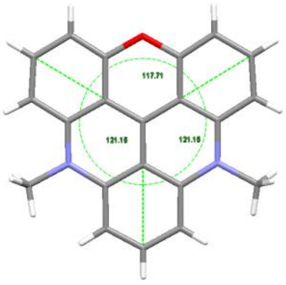

DAOTA⁺

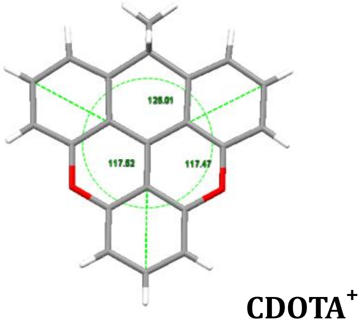

CDOTA⁺

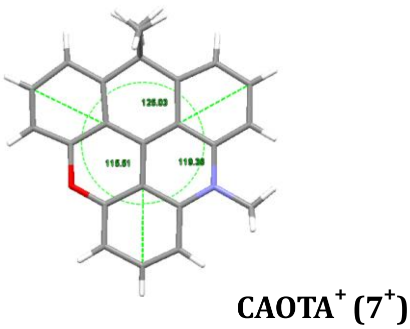

CAOTA⁺ (7⁺)

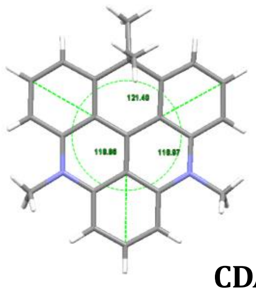

CDATA⁺ (8⁺)

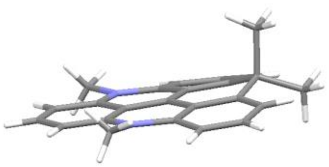

CDATA⁺ (8⁺)
side view

Figure S38. Single crystal structures viewed normal to the plane of the triangulenium system. Angles between the para positions of the triphenylene rings and the central carbon atom are indicated in green. For DAOTA$^+$, CDOTA$^+$ (**6**$^+$), CAOTA$^+$ (**7**$^+$) the triangulenium ring systems are found to be planar, for CDATA$^+$ (**8**$^+$) the bridging *sp*^{3} carbon of the isopropyl bridge is found to be 51 pm away from the plane defined by the 19 *sp*^{2} carbon atoms in the triangulenium system.

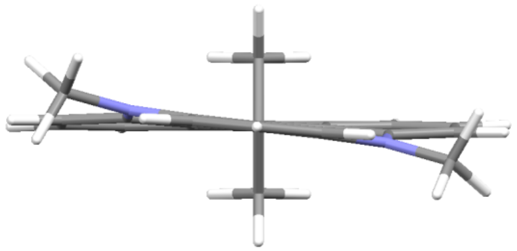

Figure S39. Side view of the **CDATA$^+$ (8$^+$)** calculated structure, the bridging nitrogen atoms are found to be ±23 pm away from the plane defined by the 19 *sp*^{2} carbon atoms in the triangulenium system.

Table S8. Selected bond lengths and angles found in the crystallographic experiments (XR) and from the calculated structures (DFT Calc.).

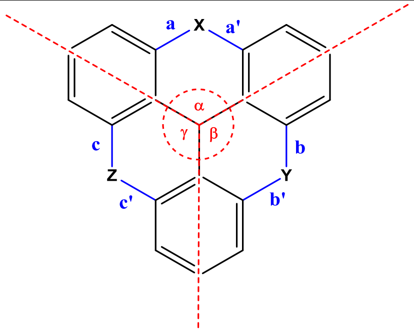

Compound	Bridge atoms<br>X, Y, Z	DATA source	Angle α (°)	Angle β (°)	Angle γ (°)	Bond length a ; a' (pm)	Bond length b ; b' (pm)	Bond length c ; c' (pm)
**DAOTA**	O, N, N	XR	117.7	121.1	121.1	139<br>139	140<br>140	140<br>140
		DFT Calc.	117.6	121.0	121.0	137<br>137	139<br>139	139<br>139
**CDOTA (6)**	C, O, O	XR	125.0	117.5	117.5	153<br>153	137<br>137	137<br>138
		DFT Calc.	125.1	117.4	117.4	154<br>154	137<br>138	137<br>138
**CAOTA (7)**	C, N, O	XR	125.0	119.4	115.5	151<br>149	141<br>132	143<br>129
		DFT Calc.	123.9	119.7	116.3	153<br>154	139<br>138	137<br>136
**CDATA (8)**	C, N, N	XR	121.4	119.0	119.0	152<br>153	138<br>138	138<br>138
		DFT Calc.	122.8	118.6	118.6	153<br>153	139<br>138	139<br>138

CIF files:

The crystallographic data in the form of CIF files have been deposited with the Cambridge Crystallographic Data Centre (CCDC) under the deposition numbers CCDC 1557677-1557680.

REFERENCES

1. C. Würth, M. Grabolle, J. Pauli, M. Spieles and U. Resch-Genger, *Nat. Protocols*, 2013, **8**, 1535-1550.
2. K. Rurack and M. Spieles, *Anal. Chem.*, 2011, **83**, 1232-1242.
3. E. N. Hooley, V. Paolucci, Z. Liao, M. R. Carro Temboury and T. Vosch, *Adv. Opt. Mater.*, 2015, **3**, 986–988.
4. Z. Liao, E. N. Hooley, L. Chen, S. Stappert, K. Müllen and T. Vosch, *J. Am. Chem. Soc.*, 2013, **135**, 19180-19185.
5. Z. Liao, M. Tropiano, S. Faulkner, T. Vosch and T. J. Sorensen, *RSC Adv.*, 2015, **5**, 70282-70286.
6. M. J. Frisch, G. W. Trucks, H. B. Schlegel, G. E. Scuseria, M. A. Robb, J. R. Cheeseman, G. Scalmani, V. Barone, B. Mennucci, G. A. Petersson, H. Nakatsuji, M. Caricato, X. Li, H. P. Hratchian, A. F. Izmaylov, J. Bloino, G. Zheng, J. L. Sonnenberg, M. Hada, M. Ehara, K. Toyota, R. Fukuda, J. Hasegawa, M. Ishida, T. Nakajima, Y. Honda, O. Kitao, H. Nakai, T. Vreven, J. A. Montgomery Jr., J. E. Peralta, F. Ogliaro, M. J. Bearpark, J. Heyd, E. N. Brothers, K. N. Kudin, V. N. Staroverov, R. Kobayashi, J. Normand, K. Raghavachari, A. P. Rendell, J. C. Burant, S. S. Iyengar, J. Tomasi, M. Cossi, N. Rega, N. J. Millam, M. Klene, J. E. Knox, J. B. Cross, V. Bakken, C. Adamo, J. Jaramillo, R. Gomperts, R. E. Stratmann, O. Yazyev, A. J. Austin, R. Cammi, C. Pomelli, J. W. Ochterski, R. L. Martin, K. Morokuma, V. G. Zakrzewski, G. A. Voth, P. Salvador, J. J. Dannenberg, S. Dapprich, A. D. Daniels, Ö. Farkas, J. B. Foresman, J. V. Ortiz, J. Cioslowski and D. J. Fox, *Journal*, 2009.
7. F. Westerlund, J. Elm, J. Lykkebo, N. Carlsson, E. Thyrhaug, B. Akerman, T. J. Sorensen, K. V. Mikkelsen and B. W. Laursen, *Photochem. Photobiol. Sci.*, 2011, **10**, 1963-1973.
8. J. Elm, J. Lykkebo, T. J. Sørensen, B. W. Laursen and K. V. Mikkelsen, *J. Phys. Chem. A*, 2011, **115**, 12025-12033.
9. J. Elm, J. Lykkebo, T. J. Sørensen, B. W. Laursen and K. V. Mikkelsen, *J. Phys. Chem. A*, 2012, **116**, 8744-8752.
10. D. Jacquemin, E. A. Perpète, I. Ciofini and C. Adamo, *Acc. Chem. Res.*, 2008, **42**, 326-334.
11. D. Jacquemin, V. Wathelet, E. A. Perpète and C. Adamo, *J. Chem. Theory Comput.*, 2009, **5**, 2420-2435.
12. S. A. Bogh, M. Simmermacher, M. Westberg, M. Bregnhøj, M. Rosenberg, L. De Vico, M. Veiga, B. W. Laursen, P. R. Ogilby, S. P. A. Sauer and T. Just Sørensen, *ACS Omega*, 2017, **2**, 193-203.
13. H. Prinz, W. Wiegrebe and K. Müller, *J. Org. Chem.* , 1996, **61**, 2853-2856.
14. P. J. Lindstrom and W. G. Mallard, *NIST Chemistry WebBook, Standard Reference Databse Number 69*, National Institute of Standards and Technology, Gaithersburg MD, 2016.
